# Molecular Identification of Secreted Effector Genes Involved in African *Fusarium oxysporum* f.sp. *elaeidis* Strains Pathogenesis During Screening Nigerian Susceptible and Tolerant Oil Palm (*Elaeis guineensis* Jacq.) Genotypes

**DOI:** 10.3389/fcimb.2020.552394

**Published:** 2020-10-06

**Authors:** Nnamdi Ifechukwude Chidi, Adedotun Adeyinka Adekunle, Temitope Oluwaseun Samuel, Emmanuel Ifechukwude Eziashi

**Affiliations:** ^1^Plant Pathology Division, Nigerian Institute for Oil Palm Research (NIFOR), Benin, Nigeria; ^2^Department of Botany, University of Lagos, Akoka, Nigeria; ^3^Plant Pathology Division, Shea Tree Crop Department, Nigerian Institute for Oil Palm Research (NIFOR), Benin, Nigeria

**Keywords:** *Fusarium oxysporum* f.sp. *elaeidis*, Fusarium wilt, pathogenesis, oil palm, virulence, incidence, severity, genetic diversity

## Abstract

Fusarium wilt is caused by *Fusarium oxysporum* f. sp. *elaeidis*, and constitutes a severe threat to the oil palm industry in Africa. This study is aimed at surveying, identifying the secreted effector genes responsible for virulence during pathogenesis, and investigating the level of genetic diversity and cluster resolutions of alleles accountable for virulence in pathogenic strains of *F. oxysporum* f.sp. *elaeidis* from African countries. Fifty-eight fungal strains were isolated from acute and chronic Fusarium wilt diseased oil palms in Nigeria, Ghana and Cameroon. Morphological and sequencing analysis of the Internal Transcribed Spacer (ITS) region grouped all strains into nine dominant strains with a majority (41.37%) belonging to *F. oxysporum*, followed by *F. solani* (20.68%), *F. equiseti* (20.68%), *F. verticilliodes* (5.17%), *F. proliferatum* (3.44%), *F. chlamydosporum* (3.44%), *F. nelsonii* (1.72%), *Fomes fomentarius*, and *Penicillium simplicissimum* (1.72%). Disease incidence and severity showed varying levels of virulence with some Fusarium strains causing severe symptoms while others exhibited slight symptoms. ISSR evaluation disclosed a considerable level of genetic diversity among pathogenic *F. oxysporum* f.sp. *elaeidis* strains. Molecular characterization using defense gene primers revealed that the oil palm genotypes screened did not amplify defense genes. During pathogenesis, *Fusarium* strains produced GMC oxidoreductases, hypothetical proteins, *FOIG 16629, FOXG 14258*, and Pyranose dehydrogenase 3-like proteins using virulent effector gene primers. Polymerase Chain Reaction analysis using specific gene primers revealed that *PRK02106, beta* and *BetA* effector genes were secreted explicitly by *F. oxysporum* f.sp. *elaeidis* (4) and *F. oxysporum* f.sp. *elaeidis* (CRT) strains while screening tolerant oil palm genotypes. During screening susceptible oil palm genotypes, *F. oxysporum* f.sp. *elaeidis* (4) and *F. oxysporum* f.sp. *elaeidis* (CRT) strains produced *FGGY_L-XK1, PRK10939, FGGY_N1, XylB1, XylB2, FGGY_L-XK2, XylB3, FGGY_N2*, and *XylB4* effector genes. Identifying these effector genes will provide the platform to study the basis of pathogenesis which will help breeders to modify breeding techniques for the improvement of oil palm genotypes in order to reduce oil palm loss in plantations and enhance food security.

## Introduction

Oil palm, which is also known as *Elaeis guineensis* Jacq., is a plant that is enduring and can last for an indefinite time. It is of African origin and grown by people for food and other uses, especially on a large farming scale. Oil palm is the ultimate oil-yielding plant in the world. Nigeria is the fourth significant palm oil producer in the world and first in Africa. More than 30% of vegetable oil is extracted from this plant, thereby making it the leader in world fat supply (United States Department of Agriculture, 2015). In as much as humans utilize oil palm produce for food, the extracted oil can also serve as a body insulator and an energy source to populations that live in developing countries (Goh et al., 2016).

The plant is in danger to so many diseases. The most destructive of these diseases in Africa is the Fusarium wilt disease whose causal agent is *Fusarium oxysporum* f.sp. *elaeidis* (Paterson et al., 2013; Noumouha et al., 2014). Fusarium wilt disease causes damage to oil palm seedlings in the nursery as well as to juvenile and mature palms in plantations. In the nursery seedlings, the symptoms commence with stunted growth, followed by yellowing of leaves, dryness and death. *F. oxysporum* strains can live in the soil and can be transmitted by both wind and rain (Leslie and Summerell, 2006). Africa's oil palm production is hugely declined due to the effects of Fusarium wilt disease (Ntsomboh et al., 2015). The damaging implications of this disease are as high as 70%, and the plantations where the condition had existed shows preference to the sudden emergence of the disease (Rival, 2017).

Plants are well-organized and are competent in protecting themselves against agents of disease through the commencement of very highly advanced and complicated defense systems (Bari and Jones, 2009). These fortifying or protective reactions encompass the release of defense-related proteins (Bowles, 1990). The effort of *Fusarium* strains in overcoming resistance is very insignificant if oil palm resistance genes are polygenic, but significant if it is based only on a few genes (Diener and Ausubel, 2005; Cooper and Rusli, 2014). The constitution of the primary cell wall makes it impenetrable to the entry of disease-causing agents. Traditionally, the enzymatic collapse of the cell wall has been linked with plant pathogenesis (Di-Pietro et al., 2003). The responsibility of pathogenesis-related (PR) proteins involved in stress response and metabolic regulations have been identified in some plants during the beginning of pathogen infection.

The diversity of *F. oxysporum* in the environment is enormous because of the extensive isolations from its natural range. This diversity can lead to variations found in the gene sequences during pathogen and host interactions (Di Pietro and Roncero, 1998). Since it is possible to confirm the pathogenesis of a disease-causing agent by the presence of a precise gene or a group of genes secreted as virulence effectors, it will be essential in this study to identify the virulent effector genes produced at the point of infection during pathogenesis using the approach of identification of the secreted effector proteins from pathogenic *F. oxysporum* f.sp. *elaeidis* strains. Furthermore, we will look at the genetic diversity, population structure among the pathogenic strains and the cluster resolutions of the virulent alleles.

## Materials and Methods

### Disease Survey, Sample Gathering and Disease Prevalence

The disease survey was conducted from 2012 to 2015 in three randomly selected African countries, namely Nigeria, Ghana, and Cameroon, in zones where the disease is widespread and severe. In Nigeria, samples were randomly collected from the Nigerian Institute for Oil Palm Research (NIFOR) central station, Oil Palm Producing Company in Ajagbodudu, and NIFOR substation in ABAK. In Ghana, samples were randomly collected from oil palm plantations at the Oil Palm Research Institute (OPRI), Kusi, Benso Oil Palm Plantation, and Ghana Oil Palm Development Company (GOPDC). Cameroon, depicted with plantations of an area of 16.86 hectares, random samples were collected from the Institute of Agricultural Research for Development (IRAD), Ekona (South West Region), and Cameroon Development Company (CDC) of the same South-West region. The disease incidence and severity were computed as described by Ntsomboh et al. (2015). Visual ratings of the symptoms were used to assess incidence and severity. The incidence of the disease was calculated as follows: Disease incidence = Nm /Nt x100; where Nm = Number of seedlings infected in the trial plot; Nt = total Number of seedlings (healthy + sick) in the trial plot. It was assessed by using the key; H = Healthy; LS = Leaf symptom only; L&R = Leaf and Root symptoms, and R= Root symptom only. Disease severity = summation of (a × b) /n ×100; where ∑(a × b) = sum of products of the number of the infected seedlings (a) corresponding to the degree of infection (b); n = Number of infected seedlings. The severity was determined using a scale of 0–5 (Table 1). The Geographic Information System (GIS) maps of the sample location points were designed.

**Table 1:** Disease severity scale.

**Scale**	**Assessment**
0	totally green
1	Very slight brown discoloration on the cut bole of the oil palm seedling
2	slight brown discoloration on the cut bole of the oil palm seedling
3	Brown discoloration on the cut bole of the oil palm seedling
4	Dark brown discoloration
5	Very dark brown discoloration

### Asexual Resources Investigated

The asexual resources investigated were 200 oil palm trees displaying chronic and acute symptoms of *Fusarium* wilt disease as described by Oritsejafor (1982); Tengoua (1994); Ntsomboh et al. (2012, 2015); Chidi et al. (2018). The external symptoms of the chronic and acute state of the disease include stunted growth, yellowing of the leaves, withering and rupture of the fronds. The death of the palms precedes these symptoms within 6 months (acute type) or a few years (chronic type). The internal symptoms include blackening, necrosis of the cortex and clogging of the vascular system.

### Isolation of *F. oxysporum* f.sp. *elaeidis* and Secondary Pathogens

*F. oxysporum* f.sp. *elaeidis* strains and secondary pathogens were isolated from the infected strands of plant tissues, petioles and soil samples of adult oil palms displaying chronic and acute symptoms of *Fusarium* wilt disease as described by Ntsomboh et al. (2015). The strands of plant tissues and petioles of infected oil palms were chopped into reduced portions with the aid of a sterile knife to expose the infected inner parts as described by Tengoua (1993). They were aseptically taken out and plated on previously prepared mycelium medium (MM) and Komada medium that has been cooled and supplemented with streptomycin antibiotic as described by Komada (1975); Tengoua (1993); Ntsomboh et al. (2015). Root samples were plated out, but not after the sliced portions had been superficially sanitized with 1% bleach combination. For the soil samples, the serial dilution method of Oritsejafor (1982) was employed. Incubation was conducted at room temperature of 26–29°C. The developing fungi colonies were aseptically subcultured into gelled prepared potato dextrose agar (PDA, Difco Laboratories, Detroit, USA) plates until pure cultures were gotten.

### Morphological Identification of *Fusarium* Strains and Secondary Pathogens

The induced fungal colonies were viewed under the light microscope. Then the images of the organisms were snapped using a Motic Camera, with model number 230 (1.3 Megapixel). The *Fusarium* strains and secondary pathogens were identified by comparing their morphology and color with fungi descriptions in Booth (1975); Leslie and Summerell (2006).

### Molecular Identification of *Fusarium* Strains and Secondary Pathogens

#### Extraction of DNA Using the Modified CTAB Method

*Fusarium* strains and other associated fungi were grown overnight in a *Fusarium* liquid medium and transferred to Eppendorf tubes. The tubes were spun at 14,000 rpm for 2 min. The supernatants were discarded, and then 600 μl of 2X CTAB buffer was added to the pellets, which were later incubated at 65°C for 30 min. The samples were taken out and left to cool down to room temperature, after which chloroform was added. The samples were stirred together by gentle inversion of the tubes several times. After that, the samples were spun at 14,000 rpm for 15 min and then transferred into new Eppendorf tubes. An equivalent volume of cold Isopropanol was added to precipitate the DNA. The samples were put in the freezer for 1 h and later spun at 14,000 rpm for 10 min. The supernatants were discarded, and pellets were washed with 70% ethanol, which was later air-dried for 30 min on the bench. The pellets were re-suspended in 100 μl of sterile distilled water.

#### DNA Electrophoresis

Agarose gel electrophoresis was used to clarify the quality and integrity of the DNA by size fractionation on 1.0% agarose gels. Agarose gels were set up by dissolving and boiling 1.0 g agarose in 100 ml 0.5 X TBE buffer solutions. The gels were left to cool down to about 45°C. Then 10 μl of 5 mg/ml detergent free ethidium bromide was added, mixed before pouring into an electrophoresis compartment set with the combs inserted. After the gels had solidified, 3 μl of the DNA, plus 5 μl sterile distilled water, and 2 μl of 6X loading dye were mixed. These mixtures were later loaded in the wells created. Electrophoresis was done at 80 V for 2 h. The separated DNA bands were visualized and photographed under UV light source.

#### PCR Analysis Using ITS1 and ITS4 Primers

PCR analysis was run using two oligonucleotide primers for fungi ITS1 (5′-TCTGTAGGTGAACCTGCGG-3′) and ITS4 (5′-TCCTCCGCTTATTGATATGC-3′) to amplify the Internal Transcribed Spacer region. The PCR mix consists of 1 μl of 10X buffer, 0.4 μl of 50 mM MgCl2, 0.5 μl of 2.5 mMdNTPs, 0.5 μl 5 mM ITS1, 0.5 μl 5 mM ITS4, 0.05 μl of 5 units/μlTaq, with 2 μl of template DNA, and then 5.05 μl of distilled water to make-up 10 μl reaction mix. The PCR profile used had an initial denaturation temperature of 94°C for 3 min, followed by 30 cycles of 94°C for 60 s, 60°C for 60 s, 72°C for 120 s and the final extension temperature of 72°C for 5 min and 10°C hold.

#### Purification of PCR Products

The amplicons were further purified before sequencing using 2 M sodium acetate wash techniques. To about 10 μl of the PCR products, 1 μl 2 M NaAct pH 5.2 was supplemented, followed by 20 μl absolute ethanol, kept at −20°C for 1 hr. The amplicons were spun at 10,000 rpm for 10 min and then washed with 70% ethanol before air drying. Re-suspension was done in 5 μl sterile distilled water and keep at 4°C for sequencing.

#### PCR for Sequencing

The PCR mix used contained 0.5 μl of BigDye Terminator Mix, 1 μl of 5X sequencing buffer, 1 μl of ITS primer with 6.5 μl distilled water, and 1 μl of the PCR product, leading to a total of 10 μl. The PCR profile for sequencing was a Rapid profile. The initial Rapid thermal ramp was set at 96°C for 1 min, followed by 25 cycles of the Rapid thermal ramp at 96°C for 10 s; Rapid thermal ramp at 50°C for 5 s; and Rapid thermal ramp at 60°C for 4 min, then followed by a Rapid thermal ramp at 4°C hold.

#### Purification of PCR Sequencing Products

The PCR sequence products were also purified before running the sequencing procedure using 2 M sodium acetate wash techniques. To 10 μl of the PCR product, 1 μl 2 M NaAct, with a pH of 5.2 was included, then 20 μl absolute ethanol, kept at −20°C for 1 hr was added. The entire mix was spun at 10,000 rpm for 10 min; washed with 70% ethanol, and then air-dried. The products were later re-suspended in 5 μl sterile distilled water and kept at 4°C for sequencing.

#### Preparation of Samples for Gene Sequencing

The Cocktail mix was a combination of 9 μl of Hi di Formamide, together with 1 μl of Purified sequence products, making a total of 10 μl. The samples were loaded on the ABI 3130 × l automated sequencing machine. The forward and reverse sequences were manually edited to avoid any ambiguities.

#### ITS Data Analysis

The BLAST searches were performed using the GenBank sequence database to confirm the identity of the sequence of the fungal strains (Geiser et al., 2004). The output from BLAST algorithms was used to query any unknown sequences against the database of all the fungal strains in the gene regions. MEGA X software (Tamura et al., 2013) was used to edit and align the nucleotide data, after which the alignments were improved manually.

#### Phylogenetic Analysis

Phylogenetic analyses were achieved using amplified PCR product generated from ITS 1 and ITS 4 primers. The BLAST algorithm performed the sequence similarity searches. MEGA X was used to generate the dendrogram as described by Nei and Kumar (2000); Tamura et al. (2013).

### Pathogenesis and Evaluation of Oil Palm Seedlings

#### Preparation of Oil Palm Germinating Seeds

The seeds were sprouted by the dry heat treatment method. Harvested seeds from susceptible and tolerant oil palm trees were soaked in plastic bowls containing water for 1 day (Table 2). It was to ensure a moisture content level of 17–18%. The floated seeds were discarded. Seeds were later transferred to white transparent polythene bags, tightly sealed with a band. Polythene bags containing the seeds were weighed at intervals to continually check that the moisture content had not declined below 17%. Heat was then applied at 37–39°C for 50 days. After the expiration of 50 days, the seeds were soaked for 3 days to jack up the moisture content to 21–23%. After drying, seeds were subjected to mild fungicide treatment, which was succeeded by 8 h of air-drying. The seeds were finally put back in polythene bags and sealed to give it an ambient temperature of around 25–30°C. At the end of 1 month, seeds were inspected for germination and processed for field trials. The parents of the oil palm genotypes are represented in Table 3.

**Table 2:** Background derivation of susceptible and tolerant oil palm genotypes screened.

**S/no**	**Field(S) Code**	**Palm number**	**Pedigree**	**Genotypes**
1	25 (4.17 × 4.17)	120	Aba dura	1
2	25 (3.361 × 1.53)	2,211	Calabar x dura	2
3	25 (32.2824 × 1.2209)	3,023	Angola Dura	3
4	25 (5.1225 × G145)	2,478	Serdang ave deli dura	4
5	25 (26/0932 × G144)	3,456	Malaya deli dura	5
6	54 (31.5703d × 31.5703d)	1,621	Ecuador dura	6
7	54 (25.3337d × 25.3337d)	1,723	Ecuador dura	7
8	25 (6.594 × 5.1450)	4,189	Calabar tenera	8

**Table 3:** Parentage and origin of oil palm genotypes.

**S/no**	**Codes**	**Origin**	**Types of fruit**
1	P3	A cross between Aba and Calabar	Nigerian tenera
2	BB4	An Ecuador deli	Deli dura
3	P8	A cross between Ufuma and Angola	Nigerian tenera
4	P1	A cross between Ufuma and Aba	Nigerian tenera

#### Seed and Soil Preparation for Pathogenesis

The germinating seeds obtained were planted with their plumules facing upwards at a depth of 2.54 cm in black polythene bags filled with topsoil (1 seed in 3 kg of soil). The poly bags containing the seeds were watered twice daily. After 2 months (two-leaf stage), initial heights measurements of the seedlings were taken using a meter rule and recorded.

#### Preparation of Conidial Suspensions

Preparations of conidial suspensions were done by cutting 3 mm portions of the already grown *F. oxysporum* f.sp. *elaeidis* strains in Petri dishes. The cutoff portions of the cultures were inoculated into 250 ml conical flasks containing sterilized 50 ml of Armstrong medium. At each preparation, flasks were inoculated with *F. oxysporum* f.sp. *elaeidis* strain and incubated for 14 days at room temperature (26–29°C). After the incubation period had elapsed, contents in the flasks were poured out into a warren blender (Model MSE, London, serial no: 7702164) and macerated. The macerated mixtures were then filtered through a muslin cloth. The supernatant was decanted, and the suspension was filled with sterile water to the required spore concentration.

#### Determination of Spore Count

A drop of the inoculum was pipetted on a hemocytometer and covered with a coverslip. A phase-contrast illumination microscope with a 10x ocular and 4 mm objective for at least 20 squares was used to count the spores. The average number per square was determined. Three counts of diluted spore suspension were made, and their average was taken. The number per milliliter of the original spore suspension (undiluted) was calculated as follows:

(Average No. per square) × y × 4,000,000

Explanation of factors:

Area of square = 1/20 × 1/20 mm = 1/400 mm^2^

Volume over square =1/400 mm^2^ × 1/10 mm (depth)

= 1/4,000 mm^3^

= 1/4,000 mm^3^ × 1/1,000 ml/mm^3^

= 1/4,000,000 ml

Where dilution factor = y.

### Pathogenesis of *F. oxysporum* f.sp. *elaeidis* Strains on Oil Palm Genotypes

The conidia suspension of *F. oxysporum* f.sp. *elaeidis* strains were inoculated on 2 months old oil palm seedlings for pathogenesis, and to determine tolerance and susceptibility of the oil palm genotypes. The pathogenesis trial was carried out by separately inoculating the roots of oil palm genotypes with conidia suspensions. The roots were exposed by removing layers of soil covering the root system. Conidia suspensions of 3.2 × 10^6^ spores/ml were dispensed into the root system. Soils were used to cover up the exposed roots. In the control experiments, sterile distilled water was used instead of spore suspension. After 1 month of inoculation, 5 g of NPK 15: 15: 20 fertilizer was applied to the inoculated seedlings. Six months post-inoculation, the seedlings were analyzed for disease symptoms.

### Statistical Analysis

Univariate Analysis of Variance with LSD plus Duncan Multiple Test was used to analyze the data for morphological parameters. The means were separated by the least significant difference at the 95% confidence interval for the difference. Bar charts were employed to compare the effect of the different *F. oxysporum* f.sp. *elaeidis* strains on the growth rate of the different oil palm genotypes.

### Identification of *F. oxysporum* f.sp. *elaeidis* Virulence Effector Genes

#### Root Inoculation for the Identification of Virulence Effector Genes

The inocula of *F. oxysporum* f.sp. *elaeidis* strains were prepared as earlier described. The seeds of tolerant (6 and 7) and susceptible (1, 2, 3, 4, and 5) oil palm genotypes used for this study were sprouted using dry heat treatment method as described earlier. The roots of the oil palm seedlings were inoculated with the inoculum of the *Fusarium oxysporum* strains as described by Renard et al. (1972); Mcfadden et al. (2006). Samples for effector gene identification analysis were harvested when external symptoms of *Fusarium* wilt disease appeared within 6 months. All spots of infected seedlings on the bole showing discolouration were taken immediately for DNA extraction. The DNA extraction was carried out using a modified CTAB method.

#### PCR Requirements

Modifications were made to the PCR protocol involving specific primers used in this study. The PCR cocktail mix consists of 2.5 ul of 10x PCR buffer,1 ul of 25 mM MgCl2, 1 ul each of forward and reverse specific primers, 1 ul of DMSO, 2 ul of 2.5 mMDNTPs, 0.1 ul of 5 u/ul Taq DNA polymerase, and 3 ul of 10 ng/ul DNA. The whole reaction volume was made up to 24 ul using 13.4 ul Nuclease-free water. The PCR cycling parameter consists of a PCR profile as follows: Initial denaturation at 94°C for 5 min, nine cycles of denaturation at 94°C for 30 s, annealing at 65°C for 30 s and elongation at 72°C for 30 s succeeded by 35 cycle sequence of denaturation at 94°C for 30 s, annealing at 55°C for 30 s, and elongation time at 72°C for 30 s. followed by a final elongation step at 72°C for 7 min and hold temperature at 10°C. Amplified fragments were visualized on 1.5% agarose electrophoresis gels. The annealing temperatures varied for the defense-related gene and virulence effector genes primers used in Table 4. After the PCR procedure, sequencing was carried out as earlier described. The sequences generated were analyzed using the BlastX searches across multiple databases (Altschul et al., 1990). The identity of the sequences was matched with sequences homologous to the fungal sequence in the database. Thus, sequences that matched a known fungal sequence with an E-value equal to or lower than 10-10 corresponding to the genes were identified.

**Table 4:** ISSR primers used for genetic diversity study, defense-related gene, and putative virulence effector genes.

**S/no**	**Primer name**	**Primer sequence 5^′^−3^′^**	**Annealing temperature ^**°**^C**	**GC Content %**	**Design basis**
1	ISSR 836	AGAGAGAGAGAGAGAGTA	57	44.4	Diversity studies
2	ISSR 858	TGTGTGTGTGTGTGTGGT	52	50	Diversity studies
3	ISSR 890	GTAGTGTGTGTGTGTGT	54	41	Diversity studies
4	ISSR 811	GAGAGAGAGAGAGAGAC	53	59.2	Diversity studies
5	UBC 901	CACACACACACACACARY	55	44	Diversity studies
6	HB-10	GAGAGAGAGAGACC	57	50	Diversity studies
7	ISSR 842	GAGAGAGAGAGAGAGATG	56	55.56	Diversity studies
8	ISSR 818	CACACACACACACACAG	48	52.94	Diversity studies
9	ISSR 827	ACACACACACACACACG	53	53	Diversity studies
10	P1 P2	CCTGATCGTGCGTGCAGTCTTGCT ATCTATGCAGAGGTCACAAGATAG	55	50	14-3-3 defense gene
11	PR-1F PR-1R	AGACGCCAGACAAGTCACCGCTAC TCCCTCGAAAGCTCAAGATAGCCC	65	50	PR-1 defense gene
12	ORX1-F ORX1-R	CCAGGCCATCAAGTTACTC CTTGTGGATATCTGAAG	55	53	Foe virulence effector gene
13	SIX4-F1 SIX4-R1	TCAGGCTTCACTTAGCATAC GCCGACCGAAAAACCCTAA	53	53	Detection of pathogenesis genes
14	SIX6-F1 SIX6-R1	CTCTCCTGAACCATCAACTT CAAGACCAGGTGTAGGCATT	53	53	Detection of pathogenesis genes

### Inter-Simple Sequence Repeat (ISSR) Diversity Evaluation

#### Sample Collection and DNA Extraction

A total of seventeen *F. oxysporum* f.sp. *elaeidis* strains were used for this investigation. The *Fusarium* strains were grown overnight in *Fusarium* liquid medium containing a mixture of Glucose (30 g); Calcium nitrate quatrehydrate (Ca (NO_3_)2. 4H_2_O (8.4 g); Sodium nitrate (2 g); Potassium dihydrogen phosphate (KH_2_PO_4_) (1.09 g); Potassium chloride (KCL) (0.22 g); Iron III chloride (FeCl_3_) (0.2 μg); Magnesium sulfate (MgSO_4_) (0.75 g); Ferrous sulfate (FeSO_4_) (trace); Zinc sulfate (ZnSO_4_) (trace); Marmite (trace); Distilled water (1,000 ml) and Agar (1 g). The total genomic DNA extraction was carried out using ZYMOGEN KIT (Zymo Research Corporation, USA), according to its manufacturer's instructions.

#### Polymerase Chain Reaction and Agarose Gel Electrophoresis

Polymerase chain reaction (PCR) amplification was accomplished by mixing 1.50 μl of 50 mM MgCl2 (BIOLINE Massachusetts, USA), 2.00 μl of 2.50 mM dNTPs (BIOLINE, Massachusetts, USA), 0.20 μl 500 U Taq DNA polymerase (BIOLINE, Massachusetts, USA), 1.0 μl of 10 μM each of ISSR primer pair, 15.05 μl of 500 ml diethylpyrocarbonate (DEPC)-treated water (INVITROGEN, Carlsbad, CA, USA) and 2.0 μL 100 ng DNA, 2.5 μl of 10 × Taq Buffer (BIOLINE, Massachusetts, USA) to make up a volume of 24.25 μL. The list of ISSR markers, their sequences, and annealing temperatures are presented in Table 4. The PCR cycling profile employed for the reaction entailed an initial step at 94 °C for 5 min, succeeded by 35 cycles of 94°C for 30 s, 72°C for 1 min, and a 10 min last extension at 72°C. For the PCR reaction, eight (8) μl of the PCR products were dispensed in a 1.5% agarose gel comprising 0.5 mg/ml ethidium bromide. The bands were photographed on Transilluminator UV light (Fotodyne Incorporated, Analyst Express, USA).

#### Data Analyses

The data matrix of ISSR profiles obtained from fragments of each amplicon was scored as 1 (presence of alleles) and 0 (absence of alleles). The data generated from the scoring of the ISSR amplicons were employed for phylogenetic reconstruction using Unweighted Pair Group Mean with Arithmetic (UPGMA) and dissimilarity index (Ojuederie et al., 2013). The analysis was carried out using NTSYSpc software version 2.02. Furthermore, genetic diversity, allele frequency, and the polymorphic information content (PIC) were analyzed using PowerMarker Version 3.25. Genetic diversity and population structure analyses of *F. oxysporum* f.sp. *elaeidis* strains were analyzed using POPGENE software version 1.32. Also, total gene diversity (Ht), gene diversity within the population (Hs), the level of gene flow (Nm), and the coefficient of gene differentiation (Gst) were calculated with POPGENE software version 1.32 (Yeh et al., 1999).

## Results

**Figure 1:**
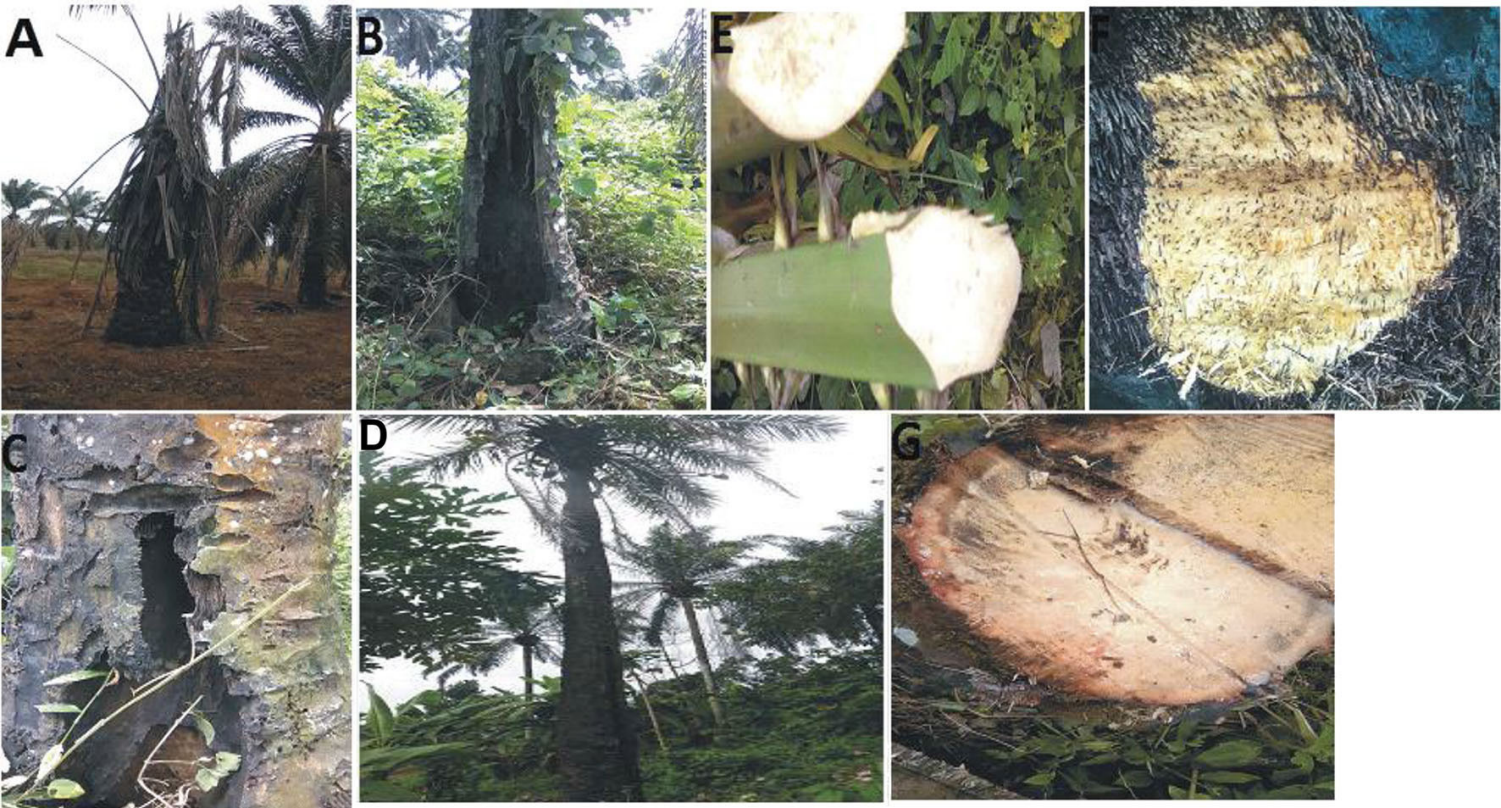
**(A)** Chronic symptoms of Fusarium wilt disease with wilting leaves. **(B)** Chronic symptoms of *Fusarium* wilt disease revealing extreme cracking of the trunk. **(C)** Chronic symptoms of *Fusarium* wilt disease with perforations in the vascular bundles. **(D)** Chronic symptoms of *Fusarium* wilt disease with a thin terminating trunk. **(E)** Infected petiole of an acute infected oil palm. **(F)** The cut transverse section of the vascular bundle of an acute *Fusarium* wilt infected oil palm. **(G)** The inner tissues of the vascular bundles of an acute *Fusarium* wilt infected oil palm.

**Figure 2:**
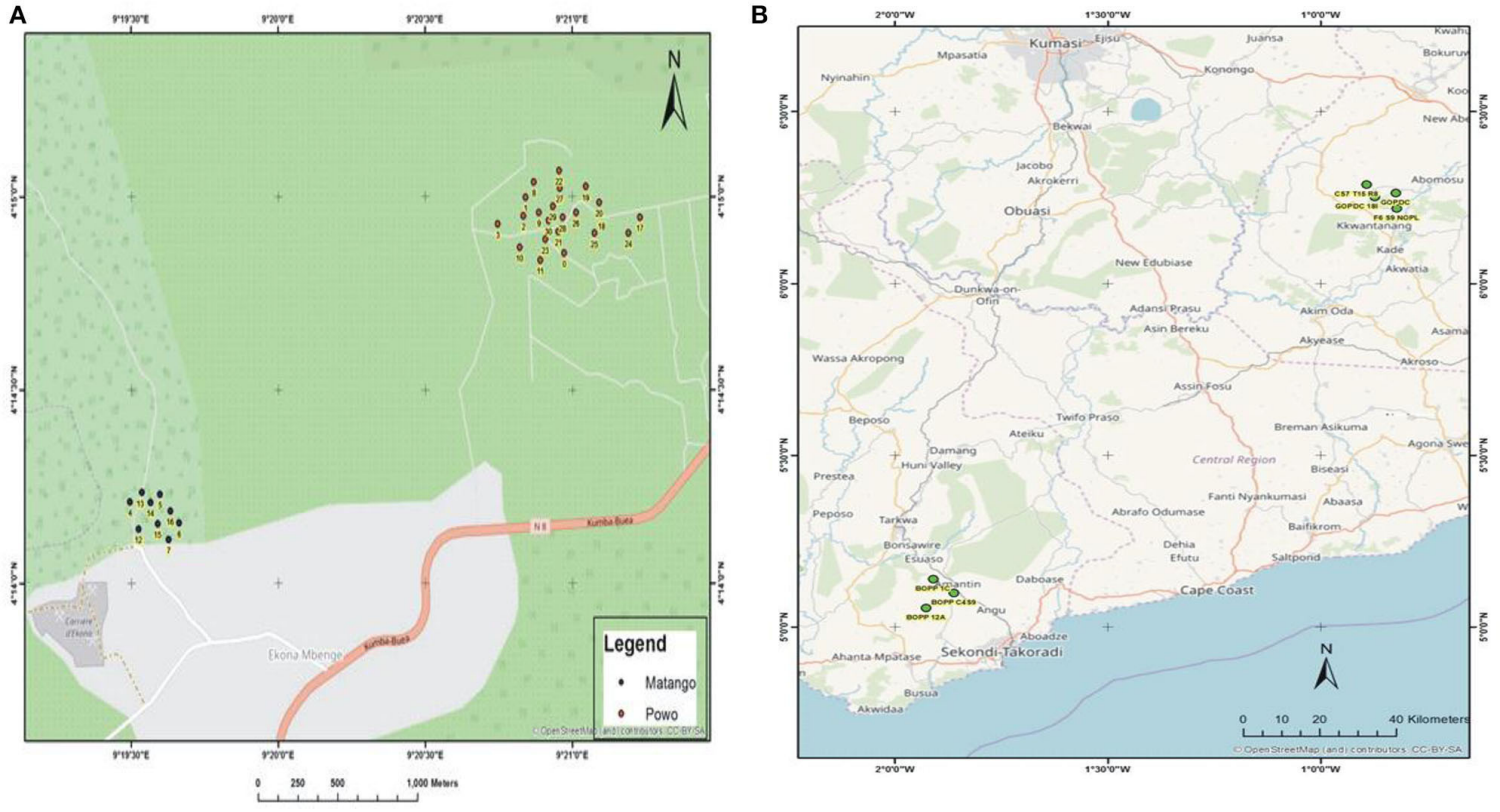
**(A)** The GIS map site of sample collection in Cameroon**. (B)** The GIS map site of sample collection in Ghana.

During the survey period, oil palm trees infected with *Fusarium* wilt disease displaying different states of chronic and acute symptoms were visited and felled (Figures 1A–D). The asexual resources investigated are represented in Figures 1E–G. The Geographical Positioning System (GPS) and the Geographical Information System (GIS) maps of the sample location points designed are presented in Supplementary Table 1, Figures 2A,B.

### Morphological and Molecular Identified *Fusarium* Strains and Other Secondary Pathogens

Fifty-six (56) *Fusarium* strains and two (2) secondary pathogens were morphologically identified and further confirmed by sequencing the ITS region of the extracted genomic DNA of the fungal strains. The sequences generated from each of the strains were used to confirm the identity of the fungal strains. The results of the BLAST queries carried out on the GenBank sequence database confirmed the identity of the fungal strains as having 95–100% homology with highly similar sequences in the GeneBank (Supplementary Table 2) (Supplementary Figure 1). The sequences of 49 fungal strains showed 100% homology with the sequence of the accessions in the GenBank (Supplementary Figure 1). Twenty-three *Fusarium* strains showed homology with 100% similarity to *F. oxysporum*. Fifteen of these *F. oxysporum* strains are from Cameroon; three from Ghana, and five from Nigeria; although one *Fusarium* strain from Cameroon showed 95% homology to *F. oxysporum*. The sequenced data generated showed that out of the 56 *Fusarium* strains identified, *F. oxysporum* was the most prevalent strain in the regions that were sampled with a percentage of 41.37%. Other strains were identified as *F. solani* (20.68%), *F. proliferatum* (3.44%), *F. equiseti* (20.68%), *F. verticilliodes* (5.17%), *F. chlamydosporum* (3.44%), *F. nelsonii* (1.72%), and other fungi strains, *Fomes fomentarius* (1.72%), and *Penicillium simplicissimum* (1.72%). Four strains of *F. oxysporum* (1) from Cameroon; *F. oxysporum* (4) from Cameroon; *F. oxysporum* (CRT) from Ghana, and *F. oxysporum* (13) from Nigeria were selected for further studies based on their degree of virulence as compared to the other *F. oxysporum* strains screened.

### Phylogenetic Investigation

**Figure 3:**
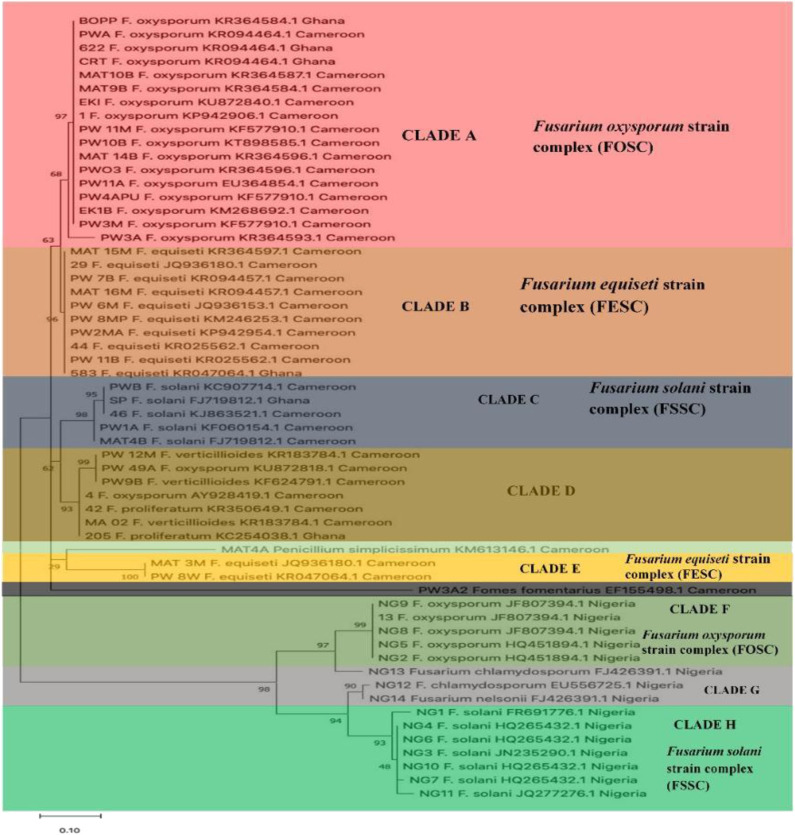
The phylogenetic tree of *Fusarium* strains and secondary pathogens using Neighbor- Joining method.

The phylogenetic investigation of the fungal pathogens from the African countries indicates that the confidence probability (multiplied by 100) means of the interior branch length is > 0, as estimated using a bootstrap test (1,000 replicates shown next to the branches). The investigation of the ITS region positioned the majority of the fungal pathogens from the acute and chronic samples in the *Fusarium* genus (Figure 3). The investigation put all fungal strains into eight main clusters. Clade A clustered *F. oxysporum* strains into the *F. oxysporum* strain complex (FOSC) representing Cameroon and Ghana. The cluster contains a virulent *F. oxysporum* strain, CRT, from Ghana, and a low virulent strain, *F. oxysporum* (1) from Cameroon. The *F. equiseti* strains (MAT 15M, PW7B, MAT 16M, PW6M, PW2MA, PW8MP, 583, 44, PW11B, and 29) formed a distinct clade B assisted by 96% bootstrap values. Clade C consists of the *F. solani* strain complex (FSSC). Interestingly, the most virulent strain, *F. oxysporum* (4) and another strain, *F. oxysporum* (PW49A) are contained in clade D with some members of the Gibberella *Fujikuroi* species complex. Clade E contained the *F. equiseti* strains mainly from Cameroon. The members of the *Fusarium oxysporum* strain complex from Nigeria are grouped in clade F. It includes a low virulent strain, *F. oxysporum* (13). *F. chlamydosporum* (NG12, NG13) and *Fusarium nelsonii* (NG14) are grouped in clade G. Clade H includes members of the *Fusarium solani* strain complex from Nigeria. *Penicillium simplicissimum* (MAT4A) and *Fomes fomentarius* (PW'3A2) from Cameroon are in the out-group.

### Disease Incidence and Severity on Oil Palm Genotypes

**Figure 4:**
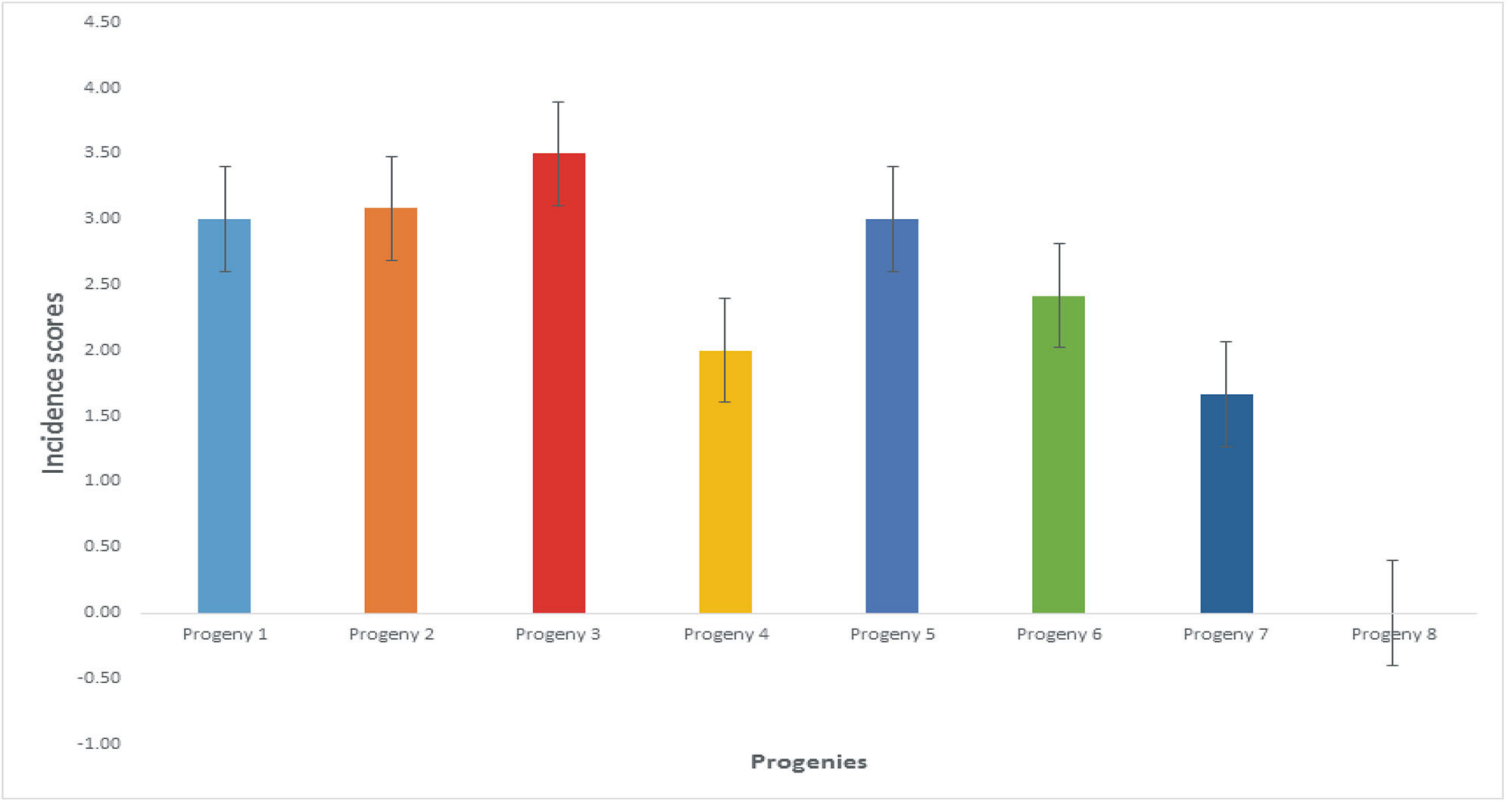
Mean incidence scores on oil palm genotypes as a result of the effects of *Fusarium* strains.

The mean disease incidence scores on the oil palm genotypes were different for all the genotypes (Figure 4) (Supplementary Table 3). The mean incidence scores on the oil palm genotypes indicate they were not significantly different from each other (*p* < 0.05) when inoculated with *F. oxysporum* f.sp. *elaeidis* (4) only. The control seedlings had no recorded incidence because they were not inoculated. In contrast, genotypes 6 and 7 had no disease incidence when inoculated with *F. oxysporum* f.sp. *elaeidis* strain (1). The effects of *F. oxysporum* f.sp. *elaeidis* strains (13) and (CRT) were not significantly different from each other on the genotypes. In contrast, the effect of *F. oxysporum* f.sp. *elaeidis* (4) differed on all other genotypes (Table 5).

**Table 5:** Mean ± SE of Score Incidence of *Fusarium oxysporum* f.sp. *elaeidis* on oil palm genotypes in the leaf and root symptom category.

**Genotypes**	***Fusarium oxysporum* f.sp**.	***Fusarium oxysporum* f.sp**.	***Fusarium oxysporum* f.sp**.	***Fusarium oxysporum* f.sp**.
	***elaeidis* 1^**a**^**	***elaeidis* 4^**c**^**	***elaeidis* 13^**b**^**	***elaeidis* CRT^**b**^**
	Mean ± SE	Mean ± SE	Mean ± SE	Mean ± SE
Genotype 1	0.67 ± 0.33^a^	6.67 ± 0.88^cd^	1.67 ± 1.67^a^	3.67 ± 2.03^a^
Genotype 2	1.67 ± 0.33^a^	7.00 ± 0.58^cd^	2.33 ± 2.33^a^	3.00 ± 0.58^a^
Genotype 3	0.33 ± 0.33^a^	7.33 ± 1.20^d^	3.00 ± 3.00^a^	3.67 ± 0.88^a^
Genotype 4	1.00 ± 1.00^a^	5.33 ± 0.67^cd^	1.33 ± 1.33^a^	1.33 ± 0.67^a^
Genotype 5	0.67 ± 0.67^a^	7.00 ± 0.58^cd^	2.33 ± 2.33^a^	2.67 ± 0.88^a^
Genotype 6	0^a^	5.00 ± 0.00^c^	1.67 ± 1.67^a^	3.00 ± 0.00^a^
Genotype 7	0^a^	2.00 ± 0.00^b^	0.67 ± 0.67^a^	4.00 ± 0.00^a^
Genotype 8	0^a^	0^a^	0^a^	0^a^
*F* – statistics	*F*_7, 16_ = 1.571;	*F*_7, 16_ = 17.252;	*F*_7, 16_ = 0.270;	*F*_7, 16_ = 2.286;
	*p* = 0.2143	*p* < 0.001	*p* = 0.957	*p* = 0.081

*NB: Genotypes with different superscripts in each column are not significantly different at 5% confidence level*.

**Figure 5:**
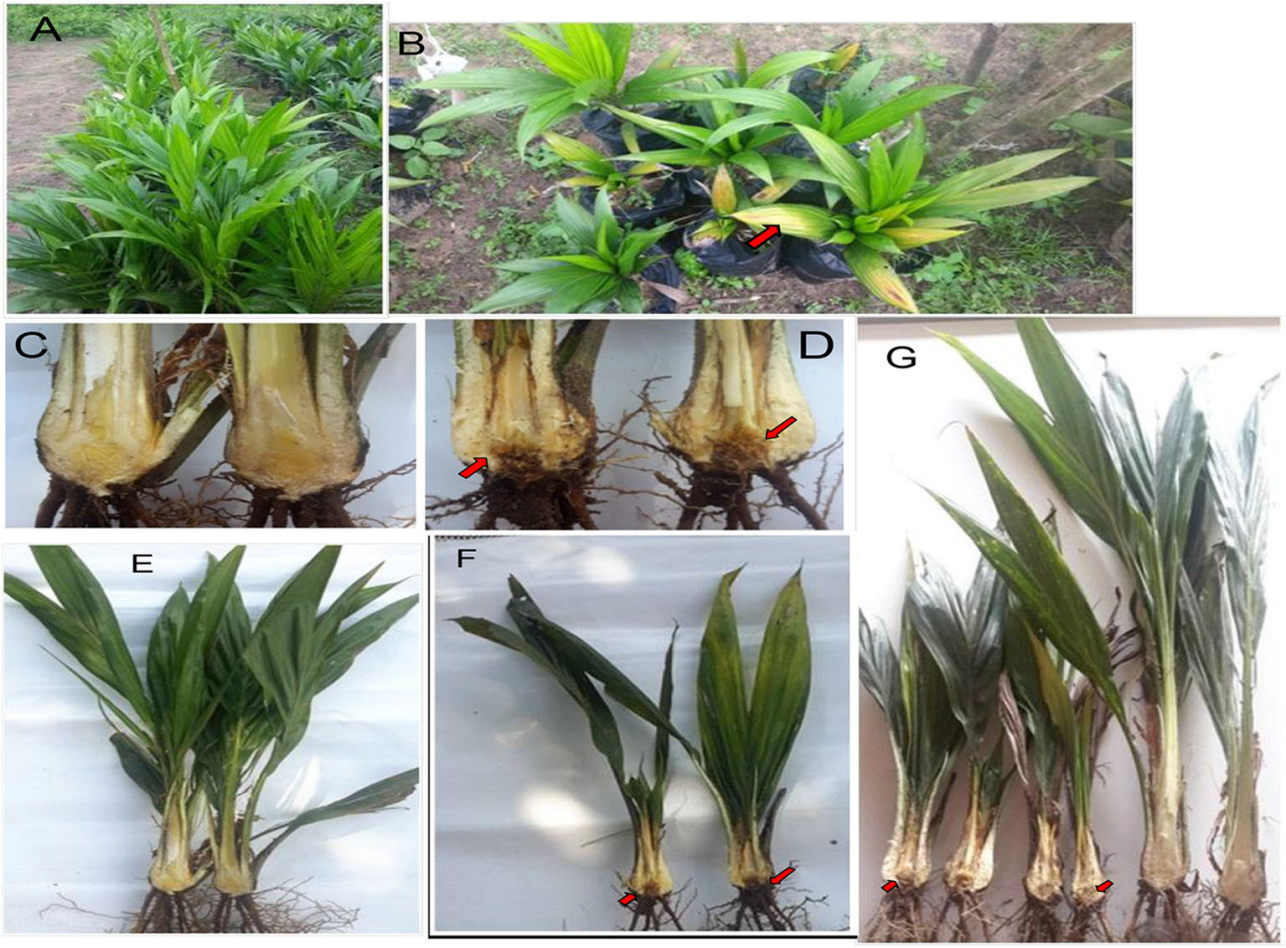
Phenotypic evaluation of oil palm genotypes. **(A,C,E)** Control of oil palm seedlings with normal height and creamy bole color. **(B,D,F)** Arrowheads indicate infected oil palm seedlings, external and internal symptoms of *Fusarium* wilt disease appearing sixty days after inoculations. **(G)** The control of oil palm seedlings differentiated itself with normal height and creamy bole color when put side by side with the diseased seedlings which exhibited stunted growth and brownish discolouration.

Fifty to sixty days after inoculation, external symptoms of *Fusarium* wilt disease began to appear. The severity of the disease caused by high-level aggressiveness of *F. oxysporum* f.sp. *elaeidis* strains is seen in the ability to colonize the roots and shoot of the oil palm seedlings (Figures 5A–G). The diseased seedlings exhibited suppressed growth and loss of vitality with chlorosis and necrosis (Figure 5B). The color of bole changed from being creamy to dark colored, which was seen in the internal tissue, a vivid sign of *Fusarium* wilt disease and symptom (Figures 5D,F). The control seedlings were differentiated with a standard height and creamy colored bole (Figures 5A–E) compared with the diseased seedlings exhibiting stunted growth and brownish discolouration (Figure 5G).

Disease severity by *Fusarium* strains showed that the severity on the different genotypes differed from each other as a result of their variance in virulence. *F. oxysporum* f.sp. *elaeidis* strain (4) had the highest disease severity of 86% on genotype 1, and severity of 77.7, 85.9, 74.8, 84, 62, and 50% on genotypes 2, 3, 4, 5, 6, and 7, respectively. *F. oxysporum* f.sp. *elaeidis* (1 and 13) had low disease severity on the oil palm genotypes as compared to *F. oxysporum* f.sp. *elaeidis* (CRT) which caused high disease severity close to *F. oxysporum* f.sp. *elaeidis* (4). Supplementary Table 4.

The adequate permissions for this particular plate 2 have been obtained from the copyright holders.

### Genetic Diversity Evaluation Revealed by Inter-Simple Sequence Repeat Molecular Markers

**Figure 6:**
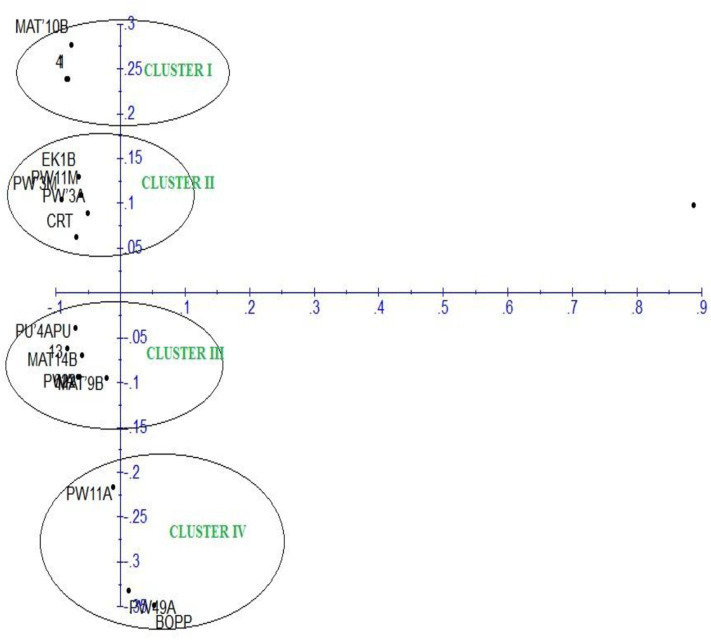
Principal component analysis of *F. oxysporum* f.sp. *elaeidis* strains based on Inter-simple sequence repeat markers.

The principal component analysis (PCA) of the generated amplicons resulted in four clusters (Figure 6). Each group was a representative of unique alleles of the strains of *F. oxysporum* f.sp. *elaeidis*. It shows the positions of unique alleles of *F. oxysporum* f.sp. *elaeidis* on the factorial axis which determined the positions of the *Fusarium* strains. Based on the positions, it separated the virulent *Fusarium* strains from other *Fusarium* strains on the factorial axis. It shows that *Fusarium* strain (4) and *Fusarium* strain (CRT) which were the most severe in the morphological screening are positioned up the principal component scale. In contrast, *Fusarium* strain (13) and *Fusarium* strain (1) which were less severe are positioned on a different factorial axis of the principal component scale.

**Figure 7:**
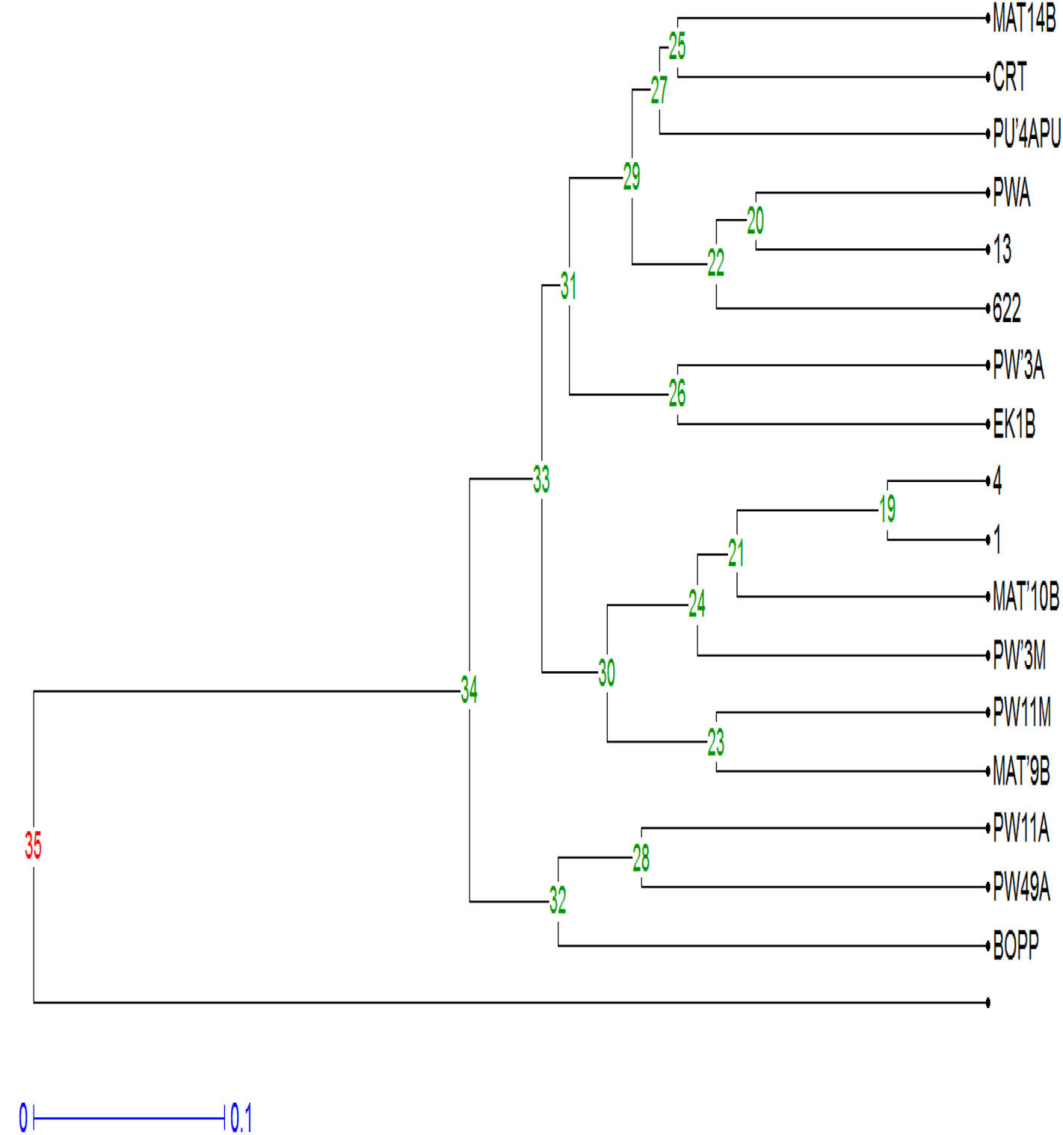
Dendrogram of *F. oxysporum* f.sp. *elaeidis* strains amplified with ISSR molecular markers.

The dendrogram of 17 strains of *F. oxysporum* f.sp. *elaeidis* was constructed using Unweighted Pair Group Mean Arithmetic (UPGMA) and dissimilarity index. It grouped the *Fusarium* strains into four major clusters, similar to the PCA (Figure 7). Cluster I grouped *Fusarium* strains MAT14B (Matango, Cameroon), CRT (Ghana), PU'4APU (Powo, Cameroon), PWA (Powo, Cameroon), 13(Nigeria) and 622(Ghana) at a bootstrap of 29%. Strain PU'4APU from Powo in Cameroon was the most genetically diverse in the group, followed by MAT14B and CRT. Cluster II contained PW3A (Powo, Cameroon) and EK1B (Ekona, Cameroon) both from Cameroon at a bootstrap of 31%. Cluster III was divided into subclusters I (SCI) and II (SCII) at a bootstrap of 30%. The SCI clustered four *Fusarium* strains at a bootstrap of 24%. The strains are 4(Cameroon) 1(Cameroon), MAT10B (Matango, Cameroon) and PW3M (Powo, Cameroon), all isolated from Cameroon. *Fusarium* strain PW3M (Powo, Cameroon) was the most genetically isolated strain in this subcluster. At 23%, the SGII clustered PW11M (Powo, Cameroon) and MAT9B (Matango, Cameroon) from Cameroon. Cluster IV had PW11A (Powo, Cameroon), PW49A (Powo, Cameroon) and BOPP (Ghana) isolated from Cameroon and Ghana. *Fusarium* strain BOPP was the most genetically dissimilar in this subcluster.

### Genetic Structure and *F. oxysporum* f.sp. *elaeidis* Strains Differentiation

The genetic diversity between PW11A (Powo, Cameroon) and PW49A (Powo, Cameroon) strains were identified to be the highest when compared with other *Fusarium* strains. Their effective number of alleles (Ne), Nei's genetic diversity (H), and Shannon's information index (I) values of 1.9621, 0.4904, and 0.6835 are represented in Table 6. In contrast, MAT10B strain was found to have the lowest genetic diversity with Ne, H, and I values of 1.1484, 0.1292, and 0.2522 when compared with other *Fusarium* strains. The genetic diversity values of these strains were ranked in a descending order as MAT10B < (1, 4) < (PWA, PW11M) < PW3M < EK1B < CRT <13 < (PWA, PU'4APU, MAT9B) < (622, BOPP) < MAT 14B < (PW49A, PW11A) from low to high. The range values of Ne, H and I obtained were 1.1484–1.9621, 0.1292–0.4904, and 0.2522–0.6835, respectively. The overall mean values and standard deviations of Ne, H and I detected in the strains using ISSR were 1.6868 ± 0.2802, 0.3889 ± 0.1177, and 0.5705 ± 0.1369, respectively.

**Table 6:** Genetic diversity and genetic differentiation parameters generated from strains of *F. oxysporum* f.sp. *elaeidis* using ISSR markers.

**S/no**	**Sample name**	**Na**	**Ne**	**H**	**I**	**Ht**	**Hs**	**Gst**	**Nm**
1	1	2.0000	1.2462	0.1975	0.3488	0.1975	0.1528	0.2266	1.7069
2	4	2.0000	1.2462	0.1975	0.3488	0.1975	0.1806	0.0859	5.3182
3	13	2.0000	1.8292	0.4533	0.6457	0.4533	0.4271	0.0579	8.1397
4	CRT	2.0000	1.8000	0.4444	0.6365	0.4444	0.4028	0.0937	4.8333
5	622	2.0000	1.9059	0.4753	0.6682	0.4753	0.4306	0.0942	4.8103
6	EK1B	2.0000	1.5643	0.3607	0.5466	0.3607	0.3229	0.1048	4.2704
7	PWA	2.0000	1.8824	0.4688	0.6616	0.4688	0.4479	0.0444	10.7500
8	PU'4APU	2.0000	1.8824	0.4688	0.6616	0.4688	0.4410	0.0593	7.9375
9	MAT14B	2.0000	1.9459	0.4861	0.6792	0.4861	0.4444	0.0857	5.3333
10	MAT10B	2.0000	1.1484	0.1292	0.2522	0.1292	0.1076	0.1672	2.4911
11	PW49A	2.0000	1.9621	0.4904	0.6835	0.4904	0.3854	0.2140	1.8364
12	BOPP	2.0000	1.9059	0.4753	0.6682	0.4753	0.4028	0.1526	2.7766
13	PW'3A	2.0000	1.4922	0.3299	0.5117	0.3299	0.2743	0.1684	2.4687
14	PW'3M	2.0000	1.5283	0.3457	0.5297	0.3457	0.3125	0.0960	4.7093
15	MAT'9B	2.0000	1.8824	0.4688	0.6616	0.4688	0.2812	0.4000	0.7500
16	PW11M	2.0000	1.4922	0.3299	0.5117	0.3299	0.1840	0.4421	0.6310
17	PW11A	2.0000	1.9621	0.4904	0.6835	0.4904	0.3854	0.2140	1.8364
	Mean	2.0000	1.6868	0.3889	0.5705	0.3889	0.3284	0.1556	2.7142
	St. Dev.	0.0000	0.2802	0.1177	0.1369	0.0139	0.0129		

*Ne, Effective number of alleles; H, Nei's gene diversity; I, Shannon's Information index; Ht total gene diversity, Hs gene diversity within the population, Gst coefficient of gene differentiation and Nm estimate of gene flow. (F. oxysporum (1) from Cameroon; F. oxysporum (4); F. oxysporum (PW11A); F. oxysporum (PW11M); F. oxysporum (PW'3M); F. oxysporum (PW'3A); F. oxysporum (PW49A); F. oxysporum (MAT'10B); F. oxysporum (MAT14B); F. oxysporum (PU'4APU); F. oxysporum (PWA); F. oxysporum (EK1B); F. oxysporum (MAT'9B) all from Cameroon); F. oxysporum (BOPP); F. oxysporum (CRT); F. oxysporum (622) all from Ghana; F. oxysporum (13) from Nigeria*.

The genetic variation among the *Fusarium* strains assessed shows that the mean values of total gene diversity (Ht), gene diversity within the population (Hs), coefficient of gene differentiation (Gst) and level of gene flow (Nm) are 0.3889, 0.3284, 0.1556, and 2.7142, respectively (Table 6). The allelic score count and frequencies were obtained from *F. oxysporum* f.sp. *elaeidis* strains using Inter-simple sequence repeat (ISSR) markers; Allele frequency, number of alleles, genetic diversity and polymorphic information content of ISSR markers (Supplementary Table 5).

### Identification of *Fusarium oxysporum* f.sp. *elaeidis* Virulence Effector Genes

**Figure 8:**
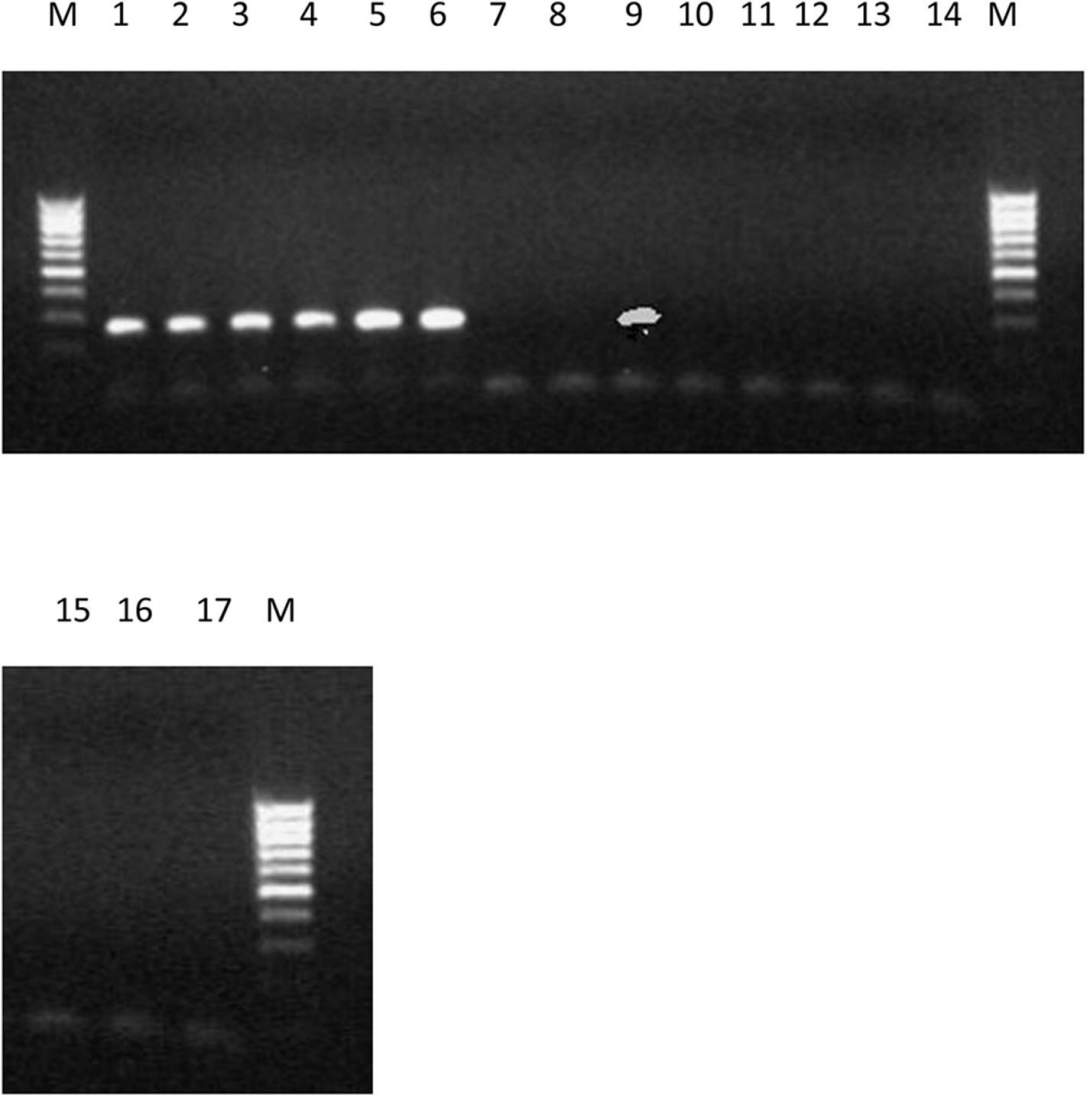
PCR products produced using primer set ORX-F1 and ORX-R1 and DNA from 17 strains of *F. oxysporum* f.sp. *elaeidis* from different African countries. The presence or absence of *ORX1* effector genes indicates virulence. Lane 1-6(4), CRT, EK1B, PWIIM, PW3M, and PW3A. Lane 7-8(1, 13); lane 9 (MAT10B); lane 10-17(PU4APU, MAT14B, MAT9B, 622, PWA, PW11A, PW49A, and BOPP).

The PCR amplification of the ORX1 effector which amplifies coding regions of secreted effector proteins from plant pathogenic fungi were used to screen the DNA from *F. oxysporum* f.sp. *elaeidis* (1); *F. oxysporum* f.sp. *elaeidis* (4); *F. oxysporum* f.sp. *elaeidis* (CRT); *F. oxysporum* f.sp. *elaeidis* (13), *F. oxysporum* f.sp. *elaeidis* (MAT 10B, EK1B, PW11M, PW3M, PW3A, PU4APU, MAT14B, MAT9B, 622, PWA, PW11A, PW49A, and BOPP in order to detect the presence or absence of candidate pathogenesis genes associated with virulence toward Nigerian oil palm genotypes. The result shows that some strains of *F. oxysporum* f.sp. *elaeidis* possess putative virulent effector genes. As a result, the *ORX1* effector genes were amplified in seven strains of *F. oxysporum* f.sp. *elaeidis* (4, CRT, MAT10B, EK1B, PWIIM, PW3M, and PW3A, while absent in the other *F. oxysporum* f.sp. *elaeidis* strains (Figure 8). The amplified sequences gotten were BLAST, and GMC oxidoreductases, hypothetical proteins *FOIG 16629, FOXG 14258, FOVG 19709, FOVG 19549*, and Pyranose dehydrogenase-3-like effector genes homologous to *F. oxysporum* in the data bank were identified (Supplementary Figure 2). The sequences generated from the PCR amplification product of the oil palm and *F. oxysporum* f.sp. *elaeidis* interaction were compared with the sequences of fungal EST's homologous to *F. oxysporum* which led to the identification of putative virulent effector genes. *PRK02106, betA*, and *BetA* effector genes were identified from susceptible oil palm genotypes. In contrast, *FGGY_L-XK 1, PRK10939, FGGY_N 1, XylB 1, XylB 2, FGGY_L-XK 2, XylB 3, FGGY_N 2* and *XylB 4* effector genes were identified from tolerant oil palm genotypes when screened with virulent strains of *F. oxysporum* f.sp. *elaeidis* (4 and CRT) (Table 7) (Supplementary Figure 3).

**Table 7:** Virulence effector genes identified from *Fusarium oxysporum* f.sp. *elaeidis* 4 and CRT post inoculation of tolerant and susceptible oil palm genotypes.

**S/no**	**Name**	**Accession**	**Description**	**Interval**	***E*-value**
1	*PRK02106*	*PRK02106*	Choline dehydrogenase; validated	74–253	2.29e-14
2	*Beta*	*TIGR01810*	Choline dehydrogenase; Choline dehydrogenase catalyzes the conversion of exogenously supplied	86–370	7.84e-12
3	*BetA*	*COG2303*	Choline dehydrogenase or related flavoprotein [lipid transport and metabolism], General	89–265	9.10e-09
4	*FGGY_L-XK*	*Cd07802*	L-xylulose kinases; a subfamily of the FGGY family of carbohydrate kinases;	413–550	0e+00
5	*PRK10939*	*PRK10039*	autoinducer-2 (AI- 2) kinase; Provisional	413–544	0e+00
6	*FGGY_N*	*Pfam00370*	FGGY family of carbohydrate kinases, N-terminal domain; this domain adopts a ribonuclease	413-544	0e+00
7	*XylB*	*TIGR01312*	D-xylulose kinase; this model describes dxylulose kinases, a subfamily of the FGGY family	410–550	0e+00
8	*XylB*	*COG1070*	Sugar (pentulose or hexulose) kinase [carbohydrate transport and metabolism];	413–550	0e+00
9	*FGGY_L-XK*	*Cd07802*	L-xylulose kinases; a subfamily of the FGGY family of carbohydrate kinases;	9–386	0e+00
10	*XylB*	*COG1070*	Sugar (pentulose or hexulose) kinase [carbohydrate transport and metabolism];	21–389	0e+00
11	*FGGY_N*	*Pfam00370*	FGGY family of carbohydrate kinases, N-terminal domain; this domain adopts a ribonuclease.	18–389	0e+00
12	*XylB*	*TIGR01312*	D-xylulose kinase	9–386	0e+00
13	*HSP70/actin*	*Pfam00370*	cell shape-determining	413–544	0e+00

*One gene can fit into more than one GO category*.

The amplification of the *ORX1* effector genes in *F. oxysporum* f.sp. *elaeidis* (4, and CRT) confirms the virulence of these strains in the pathogenesis trial. It tallies with the results obtained from the disease incidence in Table 5. Since the *ORX1* effector gene was not amplified in *F. oxysporum* f.sp. *elaeidis* (1, 13, PU4APU, MAT14B, MAT9B, 622, PWA, PW11A, PW49A, and BOPP strains, it implies that they are not pathogenic. This may be responsible for the low disease incidence caused by *F. oxysporum* f.sp. *elaeidis* (1, and 13) during pathogenesis. Additional PCR amplification was carried out on the oil palm genotypes using defense gene primers. The results indicate that there was no amplification of the *P1* and *P2* effector genes, as well as the *PR-1* genes (Supplementary Figure 4). This is consistent with the results from the pathogenesis trail during screening oil palm genotypes using *F. oxysporum* f.sp. *elaeidis* (4, and CRT).

## Discussion

This study is the first within its limits to report the phylogenetic relationship among *Fusarium* strains infecting oil palms in some parts of Africa, and identifying the virulent effector genes possessed by African *F. oxysporum* f.sp. *elaeidis* strains during pathogenesis in screening Nigerian oil palm genotypes. Despite the strains being identified morphologically, some of the strains posed difficulty in proper identification. For instance, *F. oxysporum* f.sp. *elaeidis* (4) strain from Cameroon, which was identified morphologically as *F. oxysporum* had 95% homology to *F. proliferatum* when subjected to molecular analysis. Similarly, some fungi strains like *Fomes fomentarius* and *Penicillium simplicissimum*, which could not be identified using cultural techniques were identified by comparing their BLAST sequences with the hit in the database. The result of this study is similar to El-Rabbat et al. (2018). Abd Murad et al. (2016) and Zhenyue et al. (2014) likewise found limitations in the use of morphological characteristics in characterizing pathogens in the *Gibberella fujikuroi* species complex.

The planting of alternative crops, such as maize found in some of the locations where samples were collected could be the cause of the isolation of other *Fusarium* strains, like *F. verticilliodes* and *F. proliferatum* which are pathogenic to these alternative crops. *F. proliferatum* and *F. verticilliodes* had been isolated from the diseased roots of banana and other crops (Hsuan et al., 2011; Zakaria and Rahman, 2011).

The variations in the disease incidence on the leaves and roots of the oil palm genotypes during pathogenesis showed that the seedlings responded differently to the strains of *F. oxysporum* f.sp. *elaeidis*. Ntsomboh et al. (2015) also found disparities in the disease incidence on the seeds while carrying out pathogenesis. In this study, the disease incidence observed in some tolerant and susceptible oil palm genotypes remained until the termination of the experiment. This result is in contrast with some of the tolerant seedlings recovering from the leaf symptoms (Ntsomboh et al., 2015). The disease severity caused by pathogenic strains of *F. oxysporum* f.sp. *elaeidis* in this study could be attributed to the possession of virulent effector proteins secreted in the xylem of the oil palm genotypes. Tagoe (1995) disagrees in stating that the aggressiveness of strains on oil palm is linked to the oil palm genotypes coming from the same parental cross. The color change found in the leaves of the oil palm genotypes indicates the severity of *Fusarium* wilt disease. This is similar to previous results where disease severity was observed in the leaves of oil palm genotypes treated with a pathogen (Tengoua et al., 2014; Ntsomboh et al., 2015).

The clustering of some *F. oxysporum* f.sp. *elaeidis* strains by principal component analysis (PCA) were based on their virulence and the representation of a unique allele. This study is in contrast with the clustering of *F. oxysporum* f.sp. lentis from Lentis plant not linked to the aggressiveness of the strains (Belabid et al., 2004). Furthermore, Sibounnavong (2012); Debbi et al. (2018) disagree with this study. The variations in the genetic diversity found in the strains could be attributed to ecotypic adaptations leading to mutations. The high genetic diversity shown in some of the *Fusarium* strains could be connected to the genetic recombination through a sexual cross between well-matched mating strains in the field.

In contrast, the low genetic diversity value found in *F. oxysporum* f.sp. *elaeidis* (MAT10B) could be linked to the less active asexual reproduction in that particular location where it was isolated. The coefficient of gene differentiation found in most of the *Fusarium* strains was high. This high values may be as a result of the host-pathogen relations, virulence ability and long-term resistance to unsuitable environmental conditions. This is similar to the high genetic diversity among *M. nivale* strains isolated from turfgrass (Mahuku et al., 1998).

The molecular relationship in disease are frequently not understood. An area of possible utilization involves pathogen protein effectors. When pathogens infect, they secrete a lot of virulent effectors to aid the colonization of the host. In this study, close to 193 *F. oxysporum* EST representing unique effector genes were identified as revealed in the NCBI data bank. These effector proteins represent close to 1.5% of the predicted genes in the genome. Most of these effector genes lack designated functions. This is similar to the inability to assign functions to a more significant part of the genes detected in *F. oxysporum vasinfectum* (Mcfadden et al., 2006). The PCR amplification of the *ORX1* effector genes in some of the *Fusarium* strains shows that there are presences of pathogenesis genes linked to virulence toward the oil palm genotypes. Houterman et al. (2007) also indicated that the *ORX1* genes were used as an indicator in detecting the presence or absence of pathogenicity genes. *ORX1* (in planta-secreted oxidoreductase) are conserved in *F. oxysporum* f.sp. lycopersici strain initiating tomato wilt disease and found on the pathogenesis chromosome of *F. oxysporum* f.sp. lycopersici (Ma et al., 2010). Houterman et al. (2007) and Rep et al. (2004) also reported that the *SIX* genes encode for *Avr3* protein that confers its virulence to susceptible tomato crops.

The *SIX* effector proteins were not amplified in all the *F. oxysporum* f.sp. *elaeidis* strains screened because the *SIX* proteins are limited within *Fusarium oxysporum* clade, and could be used to identify their host specificity. This is similar to *F. oxysporum lycopersici* which encompass genes encoding for *SIX* proteins (Lievens et al., 2009). Rep et al. (2004) found the majority of the *SIX* genes conserved in all *F. oxysporum* f.sp. *lycopersici* strains and not in other *Fusarium* strains. Chakrabarti et al. (2011) reported the *SIX* gene homolog in *F. oxysporum* f.sp. *vasinfectum* found in Australia. In this study, the oil palm genotypes screened did not amplify any of *P1, P2*, and *PR-1* defense genes because of the virulent effector genes produced by the virulent strains of *F. oxysporum* f.sp. *elaeidis* during pathogenesis. This is similar to plant pathogenic microorganisms using minute secreted proteins called effectors which can suppress the immune responses from the host plants (Dowd et al., 2004; Houterman et al., 2009).

The Pyranose dehydrogenase 3-like protein detected in this study encodes the *PDH* genes, which enables the *Fusarium* strains to degrade the cellulose of the oil palm genotypes. In other studies, it was also found in the Basidiomycetes and wood-degrading fungi (*Trametes* sp. and *Phanerochaete* sp.). Jahr et al. (2000) likewise identified a protein which induces bacterial wilt symptoms in tomato crop when inoculated with phytopathogenic bacterium *Clavibacter michiganensis*. The presence of *FGGY_L-XK 1, PRK10939, FGGY_N 1, XylB 1, XylB 2, FGGY_L-XK 2, XylB 3, FGGY_N 2*, and *XylB 4* detected in susceptible oil palm genotypes during the screening must have led to the increased browning found in the bole of the susceptible oil palm genotypes since the majority of these proteins are responsible for metabolic processes involved in oxidative reactions. Mcfadden et al. (2006) reported that the genes identified from *F. oxysporum* f.sp. *vasinfectum* encode for homolog proteins involved in metabolic activities. In the tolerant genotypes, the *betA* effector gene identified is also essential in the oxidative reaction found in both gram-positive and gram-negative bacteria, *Escherichia coli, Staphylococcus xylosus* and *Sinorhizobium meliloti*. The discrimination in the effector genes secreted by the pathogenic strains of *F. oxysporum* f.sp. *elaeidis* in the xylem of the susceptible and tolerant oil palm genotypes depends on the level of the compartmentalization of the cell wall.

## Conclusion

Many methods can lead to the identification of secreted virulent effector proteins. The potential of using effector proteins has provided a platform to study the basis of pathogenesis. The comparison with genomes of different sequences could shed more light on any exceptional features linked to the pathogenesis of *F oxysporum* f.sp. *elaeidis*. However, detailed analysis involving expression of genes will be needed to reveal genes that are unique to *F. oxysporum* f.sp. *elaeidis* strains.

## Data Availability Statement

The datasets presented in this study can be found in online repositories. The names of the repository/repositories and accession number(s) can be found in the article/Supplementary Material.

## Author Contributions

NC and AA envisaged and designed the research. NC carried out the study. NC, AA, TS, and EE executed data evaluation. AA, TS, and EE reviewed the manuscript. All authors have read and approved the completed manuscript.

## Conflict of Interest

The authors declare that the research was conducted in the absence of any commercial or financial relationships that could be construed as a potential conflict of interest.
