# Supplementary material for: Molecular Identification of Secreted Effector Genes Involved in African Fusarium oxysporum f.sp. elaeidis Strains Pathogenesis During Screening Nigerian Susceptible and Tolerant Oil Palm (Elaeis guineensis Jacq.) Genotypes
Source: Front Cell Infect Microbiol. 2020 Oct 6;10:552394. doi: 10.3389/fcimb.2020.552394 (PMC7573130; doi:10.3389/fcimb.2020.552394)
Supplement: Supplementary file 1 [file Data_Sheet_1.docx]

46, *F. solani* KJ863521.1 Cameroon

TTCGAGGTCAACATTCAGAAGTTGGGTGTTTTACGGCGTGGCCGCGCCGCTCTCCAGTTGCGAGGTGTTAGCTACTACGCAATGGAAGCTGCGGCGGGACCGCCACTGTATTTGGGGGACGGCGTTGCGCCCACAGGGGGCTTCCGCCGATCCCCAACGCCAGGCCCGGGGGCCTGAGGGTTGTAATGACGCTCGAACAGGCATGCCCGCCAGAATACTGGCGGGCGCAATGTGCGTTCAAAGATTCGATGATTCACTGAATTCTGCAATTCACATTACTTATCGCATTTCGCTGCGTTCTTCATCGATGCCAGAGCCAAGAGATCCGTTGTTGAAAGTTTTGATTTATTTGCTTGTTTACTCAGAAAAAACATTATAGAAACAGAGTTAGGGGGTCCTCTGGCGGGGGCGGTCCGTGTTACGGGGCCGACTGTTCCCGCCGAGGCAACGTTTTACGTATGCCCACAGGGTTGATGAGTCGTATAACTCGGCACTGATSCCTCCGGTGTGGCCCCAGCGGATACCTTGGTACAACTT

4, *F. oxysporum* AY928419.1 Cameroon

ATTCCCCTCGCGAAACCCGAGGTCAACATTCACGAAGTTGGGGGTTTAACGGCTTGGCCGCGCCGCGTACCACTTTGCGAGGGTTTTACTACTACGCAATGGAAGCTGCAGCGAGACCGCCACTAGATTTCGGGGCCGGCTTGCCGCGAGGGCTCGCCGATCCCCAACACCAAACCCGGGGGCTTGAGGGTTGAAATGACGCTCGAACAGGCATGCCCGCCAGAATACTGGCGGGCGCAATGTGCGTTCAAAGATTCGATGATTCACTGAATTCTGCAATTCACATTACTTATCGCATTTTGCTGCGTTCTTCATCGATGCCAGAACCAAGAGATCCGTTGTTGAAAGTTTTGATTTATTTATGGTTTTACTCAGAAGTTACATATAGAAACAGAGTTTAGGGGTCCTCTGGCGGGCCGTCCCGTTTTACCGGGAGCGGGCTGATCCGCCGAGGCAACAATTGGTATGTTCACAGGGGTTTGGGAGTTGTAAACTCGGTAATGATCCCTCCGCTGGTTCACCAACGGAGACCTTGTTACGACTTTTACTTCCA

EK1B, *F. oxysporum* KM268692.1 Cameroon

TCCTGATCCGAGGTCAACATTCAGAAGTTGGGGTTTAACGGCGTGGCCGCGACGATTACCACCTATGGAGGGTTTTACTACTACGCTATGGAAGCTCGACGTGACCGCCAATCAATTTGAGGAACGCGAATTAACGCGAGTCCCAACACCAAGCTGTGCTTGAGGGTTGAAATGACGCTCGAACAGGCATGCCCGCCAGAATACTGGCGGGCGCAATGTGCGTTCAAAGATTCGATGATTCACTGAATTCTGCAATTCACATTACTTATCGCATTTTGCTGCGTTCTTCATCGATGCCAGAACCAAGAGATCCGTTGTTGAAAGTTTTGATTTATTTATGGTTTTACTCAGAAGTTACATATAGAAACAGAGTTTAGGGGTCCTCTGGCGGGCCGTCCCGTTTTACCGGGAGCGGGCTGATCCGCCGAGGCAACAAGTGGTATGTTCACAGGGGTTTGGGAGTTGTAAACTCGGTAATGATCCCTCCGCTGGTTCACCAACGGAGACCTTGTTACGACTTTTTACTTCCA

PW’4APU, *F. oxysporum* KF577910.1 Cameroon

AGGTCAACATTCAGAAGTTGGGGTTTAACGGCGTGGCCGCGACGATTACCAGTAACGAGGGTTTTACTACTACGCTATGGAAGCTCGACGTGACCGCCAATCAATTTGAGGAACGCGAATTAACGCGAGTCCCAACACCAAGCTGTGCTTGAGGGTTGAAATGACGCTCGAACAGGCATGCCCGCCAGAATACTGGCGGGCGCAATGTGCGTTCAAAGATTCGATGATTCACTGAATTCTGCAATTCACATTACTTATCGCATTTTGCTGCGTTCTTCATCGATGCCAGAACCAAGAGATCCGTTGTTGAAAGTTTTGATTTATTTATGGTTTTACTCAGAAGTTACATATAGAAACAGAGTTTAGGGGTCCTCTGGCGGGCCGTCCCGTTTTACCGGGAGCGGGCTGATCCGCCGAGGCAACAAGTGGTATGTTCACAGGGGTTTGGGAGTTGTAAACTCGGTAATGATCCCTCCGCTGGTTCACCAACGGAGACCTTGTTACGACTTTTTACTTCCA

42, *F. proliferatum* KR350649.1 Cameroon

ACTGATCCGAGGTCAACATTCAGAAGTTGGGGGTTTAACGGCTTGGCCGCGCCGCGTACCMGTTGCGAGGGTTTTACTACTACGCAATGGAAGCTGCAGCGAGACCGCCACTAGATTTCGGGGCCGGCTTGCCGCAAGGGCTCGCCGATCCCCAACACCAAACCCGGGGGCTTGAGGGTTGAAATGACGCTCGAACAGGCATGCCCGCCAGAATACTGGCGGGCGCAATGTGCGTTCAAAGATTCGATGATTCACTGAATTCTGCAATTCACATTACTTATCGCATTTTGCTGCGTTCTTCATCGATGCCAGAACCAAGAGATCCGTTGTTGAAAGTTTTGATTTATTTATGGTTTTACTCAGAAGTTACATATAGAAACAGAGTTTAGGGGTCCTCTGGCGGGCCGTCCCGTTTTACCGGGAGCGGGCTGATCCGCCGAGGCAACAATTGGTATGTTCACAGGGGTTTGGGAGTTGTAAACTCGGTAATGATCCCTCCGCTGGTTCACCAACGGAGACCTTGTTACGACTTTTTACTTCCA

PW11A, *F. oxysporum* EU364854.1 Cameroon

GAGGTCAACATTCAGAAGTTGGGGTTTAACGGCGTGGCCGCGACGATTACCACCTATGGAGGGTTTTACTACTACGCTATGGAAGCTCGACGTGACCGCCAATCAATTTGAGGAACGCGAATTAACGCGAGTCCCAACACCAAGCTGTGCTTGAGGGTTGAAATGACGCTCGAACAGGCATGCCCGCCAGAATACTGGCGGGCGCAATGTGCGTTCAAAGATTCGATGATTCACTGAATTCTGCAATTCACATTACTTATCGCATTTTGCTGCGTTCTTCATCGATGCCAGAACCAAGAGATCCGTTGTTGAAAGTTTTGATTTATTTATGGTTTTACTCAGAAGTTACATATAGAAACAGAGTTTAGGGGTCCTCTGGCGGGCCGTCCCGTTTTACCGGGAGCGGGCTGATCCGCCGAGGCAACAAGTGGTATGTTCACAGGGGTTTGGGAGTTGTAAACTCGGTAATGATCCCTCCGCTGGTTCACCAACGGAGACCTTGTT

PWO3, *F. oxysporum* KR364596.1 Cameroon

CCTGCATCCGAGGTCAACATTCAGAAGTTGGGGTTTAACGGCGTGGCCGCGACGATTACCAGTAACGAGGGTTTTACTACTACGCTATGGAAGCTCGACGTGACCGCCAATCAATTTGAGGAACGCGAATTAACGCGAGTCCCAACACCAAGCTGTGCTTGAGGGTTGAAATGACGCTCGAACAGGCATGCCCGCCAGAATACTGGCGGGCGCAATGTGCGTTCAAAGATTCGATGATTCACTGAATTCTGCAATTCACATTACTTATCGCATTTTGCTGCGTTCTTCATCGATGCCAGAACCAAGAGATCCGTTGTTGAAAGTTTTGATTTATTTATGGTTTTACTCAGAAGTTACATATAGAAACAGAGTTTAGGGGTCCTCTGGCGGGCCGTCCCGTTTTACCGGGAGCGGGCTGATCCGCCGAGGCAACAAGTGGTATGTTCACAGGGGTTTGGGAGTTGTAAACTCGGTAATGATCCCTCCGCTGGTTCACCAACGGAGACCTTGTTACGACTTTTACTTCCA

PW’B, *F. solani* KC907714.1 Cameroon

CCTGATTCGAGGTCAACATTCAGAAGTTGGGTGTTTTACGGCGTGGCCGCGCCGCTCTCCAGTTGCGAGGTGTTAGCTACTACGCAATGGAAGCTGCGGCGGGACCGCCACTGTATTTGGGGGACGGCGTTGCGCCCACAGGGGGCTTCCGCCGATCCCCAACGCCAGGCCCGGGGGCCTGAGGGTTGTAATGACGCTCGAACAGGCATGCCCGCCAGAATACTGGCGGGCGCAATGTGCGTTCAAAGATTCGATGATTCACTGAATTCTGCAATTCACATTACTTATCGCATTTCGCTGCGTTCTTCATCGATGCCAGAGCCAAGAGATCCGTTGTTGAAAGTTTTGATTTATTTGCTTGTTTACTCAGAAAAAACATTATAGAAACAGAGTTAGGGGGTCCTCTGGCGGGGGCGGTCCGTGTTACGGGGCCGACTGTTCCCGCCGAGGCAACGTTTTACGTATGCCCACAGGGTTGATGAGTCGTATAACTCGGCACTGATSCCTCCGGTGTGGCCCCAGCGGATACCTTGGTACAACTTTTACTTCCAAC

PW9B, *F. verticillioides* KF624791.1 Cameroon

GAAGTTGGGGGTTTAACGGCTTGGCCGCGCCGCCGACCAGTTGCGAGGGGTTTACTACTACGCAATGGAAGCTGCAGCGAGACCGCCACTAAATTTCGGGGCCGGCTTGCCGCGAGGGCTCGCCGATCCCCAACACCAAACCCGGGGGCTTGAGGGTTGAAATGACGCTCGATCAGGCATGCCCGCCAGAATACTGGTGGGCGCAATGTGCGTTCAAAGATTCGATGATTCACTGAATTCTGCAATTCACATTACTTATCGCATTTTGCTGCGTTCTTCCTCGATGCCGGAACCAAAAAATCCGTTGTTGAAAGTTTTGATTTATTTATGGTTTTACTCAAAASTTACATATAGAAACACACTTTAGGGGTCCTCTGGCGGGCCGTCCCGTTTTACCGGGAGCGGGCTGATCCGSCGAGGCAACAATTGGTATGTTCACAGGGGTTTGGGAGTTGCAAACTCAGGAATGATCCCTCCGCTGGT

MAT 15M, *F. equiseti* KR364597.1 Cameroon

AACCATTCAGAAGTTGGGGTTTTACGGCGTGGCCGCGACGATTACCTGTAACGAGGTGTATGATTACTACGCTATGGAAGCTCGACGTGACCGCCAATCGATTTGGGGAACGCGGGTTACCGCGAGTCCCAACACCAAGCTGAGCTTGAGGGTTGAAATGACGCTCGAACAGGCATGCCCGCCAGAATACTGGCGGGCGCAATGTGCGTTCAAAGATTCGATGATTCACTGAATTCTGCAATTCACATTACTTATCGCATTTTGCTGCGTTCTTCATCGATGCCAGAACCAAGAGATCCGTTGTTGAAAGTTTTGATTTATTTGTTTGTTTTACTCAGAAGTTCCACTAAAAACAGAGTTTAGGGGTCCTCGGGCGGGCCGTCCCTTTTTACGGGGCGCGGGCTGATCCGCCGAGGCAACGTATAGGTATGTTCACAGGGGTTTGGGAGTTGTAAACTCGGTAATGATCCCTCCGCTGGTTCACCAACGGAGACCTTGT

PW’1A, *F. solani* KF060154.1 Cameroon

CTGGATTCGAGGTCAACATTCAGAAGTTGGGTGTTTTACGGCGTGGCCGCGCCGCTCTCCAGTTGCGAGGTGTTAGCTACTACGCAATGGAAGCTGCGGCGGGACCGCCACTGTATTTGGGGGACGGCGTTGCGCCCACAGGGGGCTTCCGCCGATCCCCAACGCCAGGCCCGGGGGCCTGAGGGTTGTAATGACGCTCGAACAGGCATGCCCGCCAGAATACTGGCGGGCGCAATGTGCGTTCAAAGATTCGATGATTCACTGAATTCTGCAATTCACATTACTTATCGCATTTCGCTGCGTTCTTCATCGATGCCAGAGCCAAGAGATCCGTTGTTGAAAGTTTTGATTTATTTGCTTGTTTACTCATAAAAAACATTATAGAAACAGAGTTAGGGGGTCCTCTGGCGGGGGCGGCCCGTGTTACGGGGCCGTCTGTTCCCGCCGAGGCAACGTTTTAGGTATGTTCACAGGGTTGATGAGTTGTATAACTCGGTAATGATCCCTCCGCTGGTTCACCAACGGAGAACTTGTTACGACTTTTACTTCCA

MAT 14B, *F. oxysporum* KR364596.1 Cameroon

ATATACCCCGTGGCCCCGARGTCAGCATTCACTAAGTTGGGGTTTAACGGCGTGGCCGCGACGATTACCAGTATCAGAGGGTTTTACTACTACGCTATGGAAGCTCGACGTGACCGCCAATCAATTTGAGGAACGCGAATTAACGCGAGTCCCAACACCAAGCTGTGCTTGAGGGTTGAAATGACGCTCGAACAGGCATGCCCGCCAGAATACTGGCGGGCGCAATGTGCGTTCAAAGATTCGATGATTCACTGAATTCTGCAATTCACATTACTTATCGCATTTTGCTGCGTTCTTCATCGATGCCAGAACCAAGAGATCCGTTGTTGAAAGTTTTGATTTATTTATGGTTTTACTCAGAAGTTACATATAGAAACAGAGTTTAGGGGTCCTCTGGCGGGCCGTCCCGTTTTACCGGGAGCGGGCTGATCCGCCGAGGCAACAAGTGGTATGTTCACAGGGGTTTGGGAGTTGTAAACTCGGTAATGATCCCTCCGCTGGTTCACCAACGGAGACCTTGTTACGACTTTTACTTCCA

MA 02, *F. verticillioides* KR183784.1 Cameroon

ATATTCCATCCTGATCCGAGGTCACATTCAGAAGTTGGGGGTTTAACGGCTTGGCCGCGCCGCGTACCTGTTGCGAGGGTTTTACTACTACGCAATGGAAGCTGCAGCGAGACCGCCACTAGATTTCGGGGCCGGCTTGCCGCGAGGGCTCGCCGATCCCCAACACCAAACCCGGGGGCTTGAGGGTTGAAATGACGCTCGAACAGGCATGCCCGCCAGAATACTGGCGGGCGCAATGTGCGTTCAAAGATTCGATGATTCACTGAATTCTGCAATTCACATTACTTATCGCATTTTGCTGCGTTCTTCATCGATGCCAGAACCAAGAGATCCGTTGTTGAAAGTTTTGATTTATTTATGGTTTTACTCAGAAGTTACATATAGAAACAGAGTTTAGGGGTCCTCTGGCGGGCCGTCCCGTTTTACCGGGAGCGGGCTGATCCGCCGAGGCAACAATTGGTATGTTCACAGGGGTTTGGGAGTTGTAAACTCGGTAATGATCCCTCCGCTGGTTCACCAACGGAGACCTTGTTACGACTTTTACTTCCA

PW10B, *F. oxysporum* KT898585.1 Cameroon

TCTTTYCCAACCTGCCCCGAGGTCACATTCAGAAGTTGGGGTTTAACGGCGTGGCCGCGACGATTACCAGTAACGAGGGTTTTACTACTACGCTATGGAAGCTCGACGTGACCGCCAATCAATTTGAGGAACGCGAATTAACGCGAGTCCCAACACCAAGCTGTGCTTGAGGGTTGAAATGACGCTCGAACAGGCATGCCCGCCAGAATACTGGCGGGCGCAATGTGCGTTCAAAGATTCGATGATTCACTGAATTCTGCAATTCACATTACTTATCGCATTTTGCTGCGTTCTTCATCGATGCCAGAACCAAGAGATCCGTTGTTGAAAGTTTTGATTTATTTATGGTTTTACTCAGAAGTTACATATAGAAACAGAGTTTAGGGGTCCTCTGGCGGGCCGTCCCGTTTTACCGGGAGCGGGCTGATCCGCCGAGGCAACAAGTGGTATGTTCACAGGGGTTTGGGAGTTGTAAACTCGGTAATGATCCCTCCGCTGGTTCACCAACGGAGACCTTGTTACGACTTTTACTTCCA

MAT’ 3M, *F. equiseti* JQ936180.1 Cameroon

TCTTGAACCGAGGTCAACCATTAAAAAGTGCTGCCGAAGCATGCGGTTTCTGGCTATCGTCTAGACGTTTTCAGAAGCGAGAATAGAATTACTGCGCTCAGAGTACGTAAAAACTCTGCCACTGGTTTTGAGGAGCTGCGTATTAGGCAGTCTCCCAACACTAAGCTAGGCTTAAGGGTTGAAATGACGCTCGAACAGGCATGCCCACTAGAATACTAATGGGCGCAATGTGCGTTCAAAGATTCGATGATTCACTGAATTCTGCAATTCACATTACTTATCGCATTTCGCTGCGTTCTTCATCGATGCCAGAACCAAGAGATCCGTTGTTGAAAGTTTTAACTTATTTCTTAGTTATGATTCAGAATGCCAAAAATTAACAAGAGTTTAGATGTCCACCGGCTTCCAGCGCTGTTTCCAGCGCCTTCCACCGAGGCAACAGTGGTAAGTTCACATGGTTTTGGGAGTTAGATAACTCTGTAATGATCCCTCCGCAGGTTCACCTACGGAGACCTTGTTACGACTTTTACTTCCA

29, *F. equiseti* JQ936180.1 Cameroon

CTGGATCCGAGGTCAACATTCAGAAGTTGGGGTTTTACGGCGTGGCCGCGACGATTACCASTATCGAGGTGTATGATTACTACGCTATGGAAGCTCGACGTGACCGCCAATCGATTTGGGGAACGCGGGTTACCGCGAGTCCCAACACCAAGCTGAGCTTGAGGGTTGAAATGACGCTCGAACAGGCATGCCCGCCAGAATACTGGCGGGCGCAATGTGCGTTCAAAGATTCGATGATTCACTGAATTCTGCAATTCACATTACTTATCGCATTTTGCTGCGTTCTTCATCGATGCCAGAACCAAGAGATCCGTTGTTGAAAGTTTTGATTTATTTGTTTGTTTTACTCAGAAGTTCCACTAAAAACAGAGTTTAGGGTCCTCGGGCGGGCCGTCCCGTTTTACGGGGCGCGGGCTGATCCGCCGAGGCAACGTATAGGTATGTTCACAGGGGTTTGGGAGTTGTAAACTCGGTAATGATCCCTCCGCTGGTTCACCAACGGAGACCTTGTTACGACTTTTTACTTCCA

PW 7B, *F. equiseti* KR094457.1 Cameroon

CCCCTGGATCCGAGGTCAACATTCAGAAGTTGGGGTTTTACGGCGTGGCCGCGACGATTACCAGTAACGAGGTGTATGATTACTACGCTATGGAAGCTCGACGTGACCGCCAATCGATTTGGGGAACGCGGGTTACCGCGAGTCCCAACACCAAGCTGAGCTTGAGGGTTGAAATGACGCTCGAACAGGCATGCCCGCCAGAATACTGGCGGGCGCAATGTGCGTTCAAAGATTCGATGATTCACTGAATTCTGCAATTCACATTACTTATCGCATTTTGCTGCGTTCTTCATCGATGCCAGAACCAAGAGATCCGTTGTTGAAAGTTTTGATTTATTTGTTTGTTTTACTCAGAAGTTCCACTAAAAACAGAGTTTAGGGGTCCTCGGGCGGGCCGTCCCTTTTTACGGGGCGCGGGCTGATCCGCCGAGGCAACGTATAGGTATGTTCACAGGGGTTTGGGAGTTGTAAACTCGGTAATGATCCCTCCGCTGGTTCACCAACGGAGACCTTGTTACGACTTTTTACTTC

MAT’4’A, *Penicillium simplicissimum* KM613146.1 Cameroon

TCCTGATCCGAGGTCACCTGGAAGATTGATTGGGGTCGCCGGCGGGCGCCGGCCGGGCCTACAGAGCGGGTGACGAAGCCCCATACGCTCGAGGACCGGACGCGGTGCCGCCGCTGCCTTTCGGGCCCGCCCCCCGGGAGCCGGGGGGCGAAGCCCAACACACAAGCCGTGCTTGAGGGCAGCAATGACGCTCGGACAGGCATGCCCCCCGGAATACCAGGGGGCGCAATGTGCGTTCAAAGACTCGATGATTCACTGAATTCTGCAATTCACATTACTTATCGCATTTCGCTGCGTTCTTCATCGATGCCGGAACCAAGAGATCCGTTGTTGAAAGTTTTAACTGATTTAGCTAATCTGCTCAGACTGCAATCTTCAGACAGAGTTCATTGGTGTCTTCGGCGGGCGCGGGCCCGGGGGCGAGTGCCCCCCGGCGGCCGTGAGGCGGGCCCGCCGAAGCAACAAGGTAGAATAAACACGGGTGGGAGGTTGGACCCAGAGGGCCCTCACTCGGTAATGATCCTTCCGCAGGTTCACCTACGGAAACCTTGTTACGACTTTTTACTTCCA

MAT 16M, *F. equiseti* KR094457.1 Cameroon

AGGGTCAACCATTCAGAAGTTGGGGTTTTACGGCGTGGCCGCGACGATTACCTGTAACGAGGTGTATGATTACTACGCTATGGAAGCTCGACGTGACCGCCAATCGATTTGGGGAACGCGGGTTACCGCGAGTCCCAACACCAAGCTGAGCTTGAGGGTTGAAATGACGCTCGAACAGGCATGCCCGCCAGAATACTGGCGGGCGCAATGTGCGTTCAAAGATTCGATGATTCACTGAATTCTGCAATTCACATTACTTATCGCATTTTGCTGCGTTCTTCATCGATGCCAGAACCAAGAGATCCGTTGTTGAAAGTTTTGATTTATTTGTTTGTTTTACTCAGAAGTTCCACTAAAAACAGAGTTTAGGGGTCCTCGGGCGGGCCGTCCCTTTTTACGGGGCGCGGGCTGATCCGCCGAGGCAACGTATAGGTATGTTCACAGGGGTTTGGGAGTTGTAAACTCGGTAATGATCCCTCCGCTGGTTCACCAACGGAGACCTTGTTACGACTTTTACTTCCA

PW’3A2, *Fomes fomentarius* EF155498.1 Cameroon

ACTGAATTGGAGGTCATATTCATAATAGGCGGTTCCTAGGCGGGAGCGAAAAGACTACCCTTATGGATGGGGTACTACTACACAATGGATCTCCTGAGACCGATCCGAATTTGCCGAACGCTAATTTTCGCGAGGACCCGACCGCGATGTGCTTGAGGCTCCAAATGACGCTCCTACTAGCATGCCCGCCATAATAGTGCTGAGAATACTGTGACACTCAARATGGTATGATCCTCTGAATACTGCAGATCGCATTGCGTATCCAATTATGCTGCGATTTTTGAATTCTGCTAACCACATAGATTATCGCATGAAACTGTGATCTATTTATGGCGAACTCAGAATAACCTATGTTGACAGATGTATTAGGATGCTCTGGAGGCSCGTACCTTTTTACCGTTAATGGCGTGAGCCGTGAAACCTACGTGCGTGAAGTGCCTCGGGGGTCGGGAAAGGCCCCCTAAGCCCAGATCCCTCCGATGGTAATGGCACGGGTGACTAGTGAATAATTTTACTTCCCACATGCCTCGTAAGGCCAGCTACAACCCATGTCAGAACTCGTTATTGATCCTTCCGCAGGTTCACCTACGGAAACCTTGTTACGACTTTTCACTTCCA

PW 12M, *F. verticillioides* KR183784.1 Cameroon

CCTGATCCGAGGTCACATTCAGAAGTTGGGGGTTTAACGGCTTGGCCGCGCCGCCGACCAGTTGCGAGGGGTTTACTACTACGCAATGGAAGCTGCAGCGAGACCGCCACTAAATTTCGGGGCCGGCTTGCCGCGAGGGCTCGCCGATCCCCAACACCAAACCCGGGGGCTTGAGGGTTGAAATGACGCTCGATCAGGCATGCCCGCCAGAATACTGGTGGGCGCAATGTGCGTTCAAAGATTCGATGATTCACTGAATTCTGCAATTCACATTACTTATCGCATTTTGCTGCGTTCTTCCTCGATGCCGGAACCAAAAAATCCGTTGTTGAAAGTTTTGATTTATTTATGGTTTTACTCAAAASTTACATATAGAAACACACTTTAGGGGTCCTCTGGCGGGCCGTCCCGTTTTACCGGGAGCGGGCTGATCCGSCGAGGCAACAATTGGTATGTTCACAGGGGTTTGGGAGTTGCAAACTCAGGAATGATCCCTCCGCTGGTCCACCAACGRATACCTTGTTTTGACTTTTACTTCCACCA

PW 11M, *F. oxysporum* KF577910.1 Cameroon

AGGTCAACATTCAGAAGTTGGGGTTTAACGGCGTGGCCGCGACGATTACCAGTAACGAGGGTTTTACTACTACGCTATGGAAGCTCGACGTGACCGCCAATCAATTTGAGGAACGCGAATTAACGCGAGTCCCAACACCAAGCTGTGCTTGAGGGTTGAAATGACGCTCGAACAGGCATGCCCGCCAGAATACTGGCGGGCGCAATGTGCGTTCAAAGATTCGATGATTCACTGAATTCTGCAATTCACATTACTTATCGCATTTTGCTGCGTTCTTCATCGATGCCAGAACCAAGAGATCCGTTGTTGAAAGTTTTGATTTATTTATGGTTTTACTCAGAAGTTACATATAGAAACAGAGTTTAGGGGTCCTCTGGCGGGCCGTCCCGTTTTACCGGGAGCGGGCTGATCCGCCGAGGCAACAAGTGGTATGTTCACAGGGGTTTGGGAGTTGTAAACTCGGTAATGATCCCTCCGCTGGTTCACCAACGGAGACCTTGTTACGACTTTTTACTTCCA

PW 6M, *F. equiseti* JQ936153.1 Cameroon

AAGGTCAACATTCAGAAGTTGGGGTTTTACGGCGTGGCCGCGACGATTACCAGTAACGAGGTGTATGATTACTACGCTATGGAAGCTCGACGTGACCGCCAATCGATTTGGGGAACGCGGGTTACCGCGAGTCCCAACACCAAGCTGAGCTTGAGGGTTGAAATGACGCTCGAACAGGCATGCCCGCCAGAATACTGGCGGGCGCAATGTGCGTTCAAAGATTCGATGATTCACTGAATTCTGCAATTCACATTACTTATCGCATTTTGCTGCGTTCTTCATCGATGCCAGAACCAAGAGATCCGTTGTTGAAAGTTTTGATTTATTTGTTTGTTTTACTCAGAAGTTCCACTAAAAACAGAGTTTAGGGTCCTCGGGCGGGCCGTCCCGTTTTACAGGGCGCGGGCTGATCCGCCGAGGCAACGTATAGGTATGTTCACAGGGGTTTGGGAGTTGTAAACTCGGTAATGATCCCTCCGCTGGTTCACCAACGGAGACCTTGTTACGACTTTTACTTCCA

PW 8MP, *F. equiseti* KM246253.1 Cameroon

ATCCGAGGTCAACATTCAGAAGTTGGGGTTTTACGGCGTGGCCGCGACGATTACCAGTAACGAGGTGTATGATTACTACGCTATGGAAGCTCGACGTGACCGCCAATCGATTTGGGGAACGCGGGTTACCGCGAGTCCCAACACCAAGCTGAGCTTGAGGGTTGAAATGACGCTCGAACAGGCATGCCCGCCAGAATACTGGCGGGCGCAATGTGCGTTCAAAGATTCGATGATTCACTGAATTCTGCAATTCACATTACTTATCGCATTTTGCTGCGTTCTTCATCGATGCCAGAACCAAGAGATCCGTTGTTGAAAGTTTTGATTTATTTGTTTGTTTTACTCAGAAGTTCCACTAAAAACAGAGTTTAGGGGTCCTCGGGCGGGCCGTCCCTTTTTACGGGGCGCGGGCTGATCCGCCGAGGCAACGTATAGGTATGTTCACAGGGGTTTGGGAGTTGTAAACTCGGTAATGATCCCTCCGCTGGTTCACC

PW’2MA, *F. equiseti* KP942954.1 Cameroon

ACATTCAGAAGTTGGGGTTTTACGGCGTGGCCGCGACGATTACCAGTAACGAGGTGTATGATTACTACGCTATGGAAGCTCGACGTGACCGCCAATCGATTTGGGGAACGCGGGTTACCGCGAGTCCCAACACCAAGCTGAGCTTGAGGGTTGAAATGACGCTCGAACAGGCATGCCCGCCAGAATACTGGCGGGCGCAATGTGCGTTCAAAGATTCGATGATTCACTGAATTCTGCAATTCACATTACTTATCGCATTTTGCTGCGTTCTTCATCGATGCCAGAACCAAGAGATCCGTTGTTGAAAGTTTTGATTTATTTGTTTGTTTTACTCAGAAGTTCCACTAAAAACAGAGTTTAGGGTCCTCGGGCGGGCCGTCCCGTTTTACAGGGCGCGGGCTGATCCGCCGAGGCAACGTATAGGTATGTTCACAGGGGTTTGGGAGTTGTAAACTCGGTAATGATC

1, *F. oxysporum* KP942906.1 Cameroon

TCCGAGGTCAACATTCAGAAGTTGGGGTTTAACGGCGTGGCCGCGACGATTACCACCTATGGAGGGTTTTACTACTACGCTATGGAAGCTCGACGTGACCGCCAATCAATTTGAGGAACGCGAATTAACGCGAGTCCCAACACCAAGCTGTGCTTGAGGGTTGAAATGACGCTCGAACAGGCATGCCCGCCAGAATACTGGCGGGCGCAATGTGCGTTCAAAGATTCGATGATTCACTGAATTCTGCAATTCACATTACTTATCGCATTTTGCTGCGTTCTTCATCGATGCCAGAACCAAGAGATCCGTTGTTGAAAGTTTTGATTTATTTATGGTTTTACTCAGAAGTTACATATAGAAACAGAGTTTAGGGGTCCTCTGGCGGGCCGTCCCGTTTTACCGGGAGCGGGCTGATCCGCCGAGGCAACAAGTGGTATGTTCACAGGGGTTTGGGAGTTGTAAACTCGGTAATGATCCCTCCGCTGGTTCACCAACGGAGACCTTGTTACGA

44, *F. equiseti* KR025562.1 Cameroon

GAGGTCAACATTCAGAAGTTGGGGTTTTACGGCGTGGCCGCGACGATTACCAGTAACGAGGTGTATGATTACTACGCTATGGAAGCTCGACGTGACCGCCAATCGATTTGGGGAACGCGGGTTACCGCGAGTCCCAACACCAAGCTGAGCTTGAGGGTTGAAATGACGCTCGAACAGGCATGCCCGCCAGAATACTGGCGGGCGCAATGTGCGTTCAAAGATTCGATGATTCACTGAATTCTGCAATTCACATTACTTATCGCATTTTGCTGCGTTCTTCATCGATGCCAGAACCAAGAGATCCGTTGTTGAAAGTTTTGATTTATTTGTTTGTTTTACTCAGAAGTTCCACTAAAAACAGAGTTTAGGGGTCCTCGGGCGGGCCGTCCCTTTTTACGGGGCGCGGGCTGATCCGCCGAGGCAACGTATAGGTATGTTCACAGGGGTTTGGGAGTTGTAAACTCGGTAATGATCCCTCCGCTGG

EKI, *F. oxysporum* KU872840.1 Cameroon

CCGAGGTCAACATTCAGAAGTTGGGGTTTAACGGCGTGGCCGCGACGATTACCAGTAACGAGGGTTTTACTACTACGCTATGGAAGCTCGACGTGACCGCCAATCAATTTGAGGAACGCGAATTAACGCGAGTCCCAACACCAAGCTGTGCTTGAGGGTTGAAATGACGCTCGAACAGGCATGCCCGCCAGAATACTGGCGGGCGCAATGTGCGTTCAAAGATTCGATGATTCACTGAATTCTGCAATTCACATTACTTATCGCATTTTGCTGCGTTCTTCATCGATGCCAGAACCAAGAGATCCGTTGTTGAAAGTTTTGATTTATTTATGGTTTTACTCAGAAGTTACATATAGAAACAGAGTTTAGGGGTCCTCTGGCGGGCCGTCCCGTTTTACCGGGAGCGGGCTGATCCGCCGAGGCAACAAGTGGTATGTTCACAGGGGTTTGGGAGTTGTAAACTCGGTAATGATCCCTCCGCTGGTTCACCAACGGAGACCTTGTTACGACTTTT

MAT’4B, *F. solani* FJ719812.1 Cameroon

TTCGAGGTCAACATTCAGAAGTTGGGTGTTTTACGGCGTGGCCGCGCCGCTCTCCAGTTGCGAGGTGTTAGCTACTACGCAATGGAAGCTGCGGCGGGACCGCCACTGTATTTGGGGGACGGCGTTGCGCCCACAGGGGGCTTCCGCCGATCCCCAACGCCAGGCCCGGGGGCCTGAGGGTTGTAATGACGCTCGAACAGGCATGCCCGCCAGAATACTGGCGGGCGCAATGTGCGTTCAAAGATTCGATGATTCACTGAATTCTGCAATTCACATTACTTATCGCATTTCGCTGCGTTCTTCATCGATGCCAGAGCCAAGAGATCCGTTGTTGAAAGTTTTGATTTATTTGCTTGTTTACTCATAAAAAACATTATAGAAACAGAGTTAGGGGGTCCTCTGGCGGGGGCGGCCCGTGTTACGGGGCCGTCTGTTCCCGCCGAGGCAACGTTTTAGGTATGTTCACAGGGTTGATGAGTTGTATAACTCGGTAATGATCCCTCCGCTGGTTCACCAACGGAGAACTTGTTACGACTTTTACTT

MAT’9B, *F. oxysporum* KR364584.1 Cameroon

CATTCAGAAGTTGGGGTTTAACGGCGTGGCCGCGACGATTACCACCTATGGAGGGTTTTACTACTACGCTATGGAAGCTCGACGTGACCGCCAATCAATTTGAGGAACGCGAATTAACGCGAGTCCCAACACCAAGCTGTGCTTGAGGGTTGAAATGACGCTCGAACAGGCATGCCCGCCAGAATACTGGCGGGCGCAATGTGCGTTCAAAGATTCGATGATTCACTGAATTCTGCAATTCACATTACTTATCGCATTTTGCTGCGTTCTTCATCGATGCCAGAACCAAGAGATCCGTTGTTGAAAGTTTTGATTTATTTATGGTTTTACTCAGAAGTTACATATAGAAACAGAGTTTAGGGGTCCTCTGGCGGGCCGTCCCGTTTTACCGGGAGCGGGCTGATCCGCCGAGGCAACAAGTGGTATGTTCACAGGGGTTTGGGAGTTGTAAACTCGGTAATGATCCCTCCGCTGGTTCACCAACGGAGACCTT

MAT’10B, *F. oxysporum* KR364587.1 Cameroon

CGTGGCCCCGARGTCAGCATTCACTAAGTTGGGGTTTAACGGCGTGGCCGCGACGATTACCAGTATCAGAGGGTTTTACTACTACGCTATGGAAGCTCGACGTGACCGCCAATCAATTTGAGGAACGCGAATTAACGCGAGTCCCAACACCAAGCTGTGCTTGAGGGTTGAAATGACGCTCGAACAGGCATGCCCGCCAGAATACTGGCGGGCGCAATGTGCGTTCAAAGATTCGATGATTCACTGAATTCTGCAATTCACATTACTTATCGCATTTTGCTGCGTTCTTCATCGATGCCAGAACCAAGAGATCCGTTGTTGAAAGTTTTGATTTATTTATGGTTTTACTCAGAAGTTACATATAGAAACAGAGTTTAGGGGTCCTCTGGCGGGCCGTCCCGTTTTACCGGGAGCGGGCTGATCCGCCGAGGCAACAAGTGGTATGTTCACAGGGGTTTGGGAGTTGTAAACTCGGTAATGATCCCTCCGCTGGTTCACCAACGGAGACCTTGTTACGACTTTTACT

PW 8W, *F. equiseti* KR047064.1 Cameroon

GAGGTCAACCATTAAAAAGTGCTGCCGAAGCATGCGGTTTCTGGCTATCGTCTAGACGTTTTCAGAAGCGAGAATAGAATTACTGCGCTCAGAGTACGTAAAAACTCTGCCACTGGTTTTGAGGAGCTGCGTATTAGGCAGTCTCCCAACACTAAGCTAGGCTTAAGGGTTGAAATGACGCTCGAACAGGCATGCCCACTAGAATACTAATGGGCGCAATGTGCGTTCAAAGATTCGATGATTCACTGAATTCTGCAATTCACATTACTTATCGCATTTCGCTGCGTTCTTCATCGATGCCAGAACCAAGAGATCCGTTGTTGAAAGTTTTAACTTATTTCTTAGTTATGATTCAGAATGCCAAAAATTAACAAGAGTTTAGATGTCCACCGGCTTCCAGCGCTGTTTCCAGCGCCTTCCACCGAGGCAACAGTGGTAAGTTCACATGGTTTTGGGAGTTAGATAACTCTGTAATGATCCCTCCGCAGGTTCACCTACGGAGACCTTGTTACGACTTTT

PW 11B, *F. equiseti* KR025562.1 Cameroon

GTCAACATTCAGAAGTTGGGGTTTTACGGCGTGGCCGCGACGATTACCAGTAACGAGGTGTATGATTACTACGCTATGGAAGCTCGACGTGACCGCCAATCGATTTGGGGAACGCGGGTTACCGCGAGTCCCAACACCAAGCTGAGCTTGAGGGTTGAAATGACGCTCGAACAGGCATGCCCGCCAGAATACTGGCGGGCGCAATGTGCGTTCAAAGATTCGATGATTCACTGAATTCTGCAATTCACATTACTTATCGCATTTTGCTGCGTTCTTCATCGATGCCAGAACCAAGAGATCCGTTGTTGAAAGTTTTGATTTATTTGTTTGTTTTACTCAGAAGTTCCACTAAAAACAGAGTTTAGGGTCCTCGGGCGGGCCGTCCCGTTTTACAGGGCGCGGGCTGATCCGCCGAGGCAACGTATAGGTATGTTCACAGGGGTTTGGGAGTTGTAAACTCGGTAATGATCCCTCCGCTGGTTCACCAACGGAGACCTTGTTACGACTT

PW 49A, *F. oxysporum* KU872818.1 Cameroon

TGATCCGAGGTCACATTCAGAAGTTGGGGGTTTAACGGCTTGGCCGCGCCGCCGACCAGTTGCGAGGGGTTTACTACTACGCAATGGAAGCTGCAGCGAGACCGCCACTAAATTTCGGGGCCGGCTTGCCGCGAGGGCTCGCCGATCCCCAACACCAAACCCGGGGGCTTGAGGGTTGAAATGACGCTCGATCAGGCATGCCCGCCAGAATACTGGTGGGCGCAATGTGCGTTCAAAGATTCGATGATTCACTGAATTCTGCAATTCACATTACTTATCGCATTTTGCTGCGTTCTTCCTCGATGCCGGAACCAAAAAATCCGTTGTTGAAAGTTTTGATTTATTTATGGTTTTACTCAAAASTTACATATAGAAACACACTTTAGGGGTCCTCTGGCGGGCCGTCCCGTTTTACCGGGAGCGGGCTGATCCGSCGAGGCAACAATTGGTATGTTCACAGGGGTTTGGGAGTTGCAAACTCAGGAATGATCCCTCCGCTGGTCCACCAACGRATACCTTGTTTTGACTTTTACTTCCA

CRT, *F. oxysporum* KR094464.1 Ghana

CAACATTCAGAAGTTGGGGTTTAACGGCGTGGCCGCGACGATTACCAGTAACGAGGGTTTTACTACTACGCTATGGAAGCTCGACGTGACCGCCAATCAATTTGAGGAACGCGAATTAACGCGAGTCCCAACACCAAGCTGTGCTTGAGGGTTGAAATGACGCTCGAACAGGCATGCCCGCCAGAATACTGGCGGGCGCAATGTGCGTTCAAAGATTCGATGATTCACTGAATTCTGCAATTCACATTACTTATCGCATTTTGCTGCGTTCTTCATCGATGCCAGAACCAAGAGATCCGTTGTTGAAAGTTTTGATTTATTTATGGTTTTACTCAGAAGTTACATATAGAAACAGAGTTTAGGGGTCCTCTGGCGGGCCGTCCCGTTTTACCGGGAGCGGGCTGATCCGCCGAGGCAACAAGTGGTATGTTCACAGGGGTTTGGGAGTTGTAAACTCGGTAATGATCCCTCCGCTGGTTCACCAACGGAGACCTT

205, *F. proliferatum* KC254038.1 Ghana

ACCCGAGGTCAACATTCACGAAGTTGGGGGTTTAACGGCTTGGCCGCGCCGCGTACCACTTTGCGAGGGTTTTACTACTACGCAATGGAAGCTGCAGCGAGACCGCCACTAGATTTCGGGGCCGGCTTGCCGCGAGGGCTCGCCGATCCCCAACACCAAACCCGGGGGCTTGAGGGTTGAAATGACGCTCGAACAGGCATGCCCGCCAGAATACTGGCGGGCGCAATGTGCGTTCAAAGATTCGATGATTCACTGAATTCTGCAATTCACATTACTTATCGCATTTTGCTGCGTTCTTCATCGATGCCAGAACCAAGAGATCCGTTGTTGAAAGTTTTGATTTATTTATGGTTTTACTCAGAAGTTACATATAGAAACAGAGTTTAGGGGTCCTCTGGCGGGCCGTCCCGTTTTACCGGGAGCGGGCTGATCCGCCGAGGCAACAATTGGTATGTTCACAGGGGTTTGGGAGTTGTAAACTCGGTAATGATCCCTCCGCTGGTTCACCAACGGAGACCTTGTTACGACTTTTACT

583, *F. equiseti* KR047064.1 Ghana

GGGGTTTTACGGCGTGGCCGCGACGATTACCAGTAACGAGGTGTATGATTACTACGCTATGGAAGCTCGACGTGACCGCCAATCGATTTGGGGAACGCGGGTTACCGCGAGTCCCAACACCAAGCTGAGCTTGAGGGTTGAAATGACGCTCGAACAGGCATGCCCGCCAGAATACTGGCGGGCGCAATGTGCGTTCAAAGATTCGATGATTCACTGAATTCTGCAATTCACATTACTTATCGCATTTTGCTGCGTTCTTCATCGATGCCAGAACCAAGAGATCCGTTGTTGAAAGTTTTGATTTATTTGTTTGTTTTACTCAGAAGTTCCACTAAAAACAGAGTTTAGGGTCCTCGGGCGGGCCGTCCCGTTTTACAGGGCGCGGGCTGATCCGCCGAGGCAACGTATAGGTATGTTCACAGGGGTTTGGGAGTTGTAAACTCGGTAATGATCCCTCCGCTGGTTCACCAACGGAGACCTTGTTACG

622, *F. oxysporum* KR094464.1 Ghana

CTGATCCGAGGTCAACATTCAGAAGTTGGGGTTTAACGGCGTGGCCGCGACGATTACCACCTATGGAGGGTTTTACTACTACGCTATGGAAGCTCGACGTGACCGCCAATCAATTTGAGGAACGCGAATTAACGCGAGTCCCAACACCAAGCTGTGCTTGAGGGTTGAAATGACGCTCGAACAGGCATGCCCGCCAGAATACTGGCGGGCGCAATGTGCGTTCAAAGATTCGATGATTCACTGAATTCTGCAATTCACATTACTTATCGCATTTTGCTGCGTTCTTCATCGATGCCAGAACCAAGAGATCCGTTGTTGAAAGTTTTGATTTATTTATGGTTTTACTCAGAAGTTACATATAGAAACAGAGTTTAGGGGTCCTCTGGCGGGCCGTCCCGTTTTACCGGGAGCGGGCTGATCCGCCGAGGCAACAAGTGGTATGTTCACAGGGGTTTGGGAGTTGTAAACTCGGTAATGATCCCTCCGCTGGTTCACCAACGGAGACCTTGTTACGA

BOPP, *F. oxysporum* KR364584.1 Ghana

CATTCAGAAGTTGGGGTTTAACGGCGTGGCCGCGACGATTACCAGTAACGAGGGTTTTACTACTACGCTATGGAAGCTCGACGTGACCGCCAATCAATTTGAGGAACGCGAATTAACGCGAGTCCCAACACCAAGCTGTGCTTGAGGGTTGAAATGACGCTCGAACAGGCATGCCCGCCAGAATACTGGCGGGCGCAATGTGCGTTCAAAGATTCGATGATTCACTGAATTCTGCAATTCACATTACTTATCGCATTTTGCTGCGTTCTTCATCGATGCCAGAACCAAGAGATCCGTTGTTGAAAGTTTTGATTTATTTATGGTTTTACTCAGAAGTTACATATAGAAACAGAGTTTAGGGGTCCTCTGGCGGGCCGTCCCGTTTTACCGGGAGCGGGCTGATCCGCCGAGGCAACAAGTGGTATGTTCACAGGGGTTTGGGAGTTGTAAACTCGGTAATGATCCCTCCGCTGGTTCACCAACGGAGACCTTGTTACGACTTTTT

SP, *F. solani* FJ719812.1 Ghana

TTCGAGGTCAACATTCAGAAGTTGGGTGTTTTACGGCGTGGCCGCGCCGCTCTCCAGTTGCGAGGTGTTAGCTACTACGCAATGGAAGCTGCGGCGGGACCGCCACTGTATTTGGGGGACGGCGTTGCGCCCACAGGGGGCTTCCGCCGATCCCCAACGCCAGGCCCGGGGGCCTGAGGGTTGTAATGACGCTCGAACAGGCATGCCCGCCAGAATACTGGCGGGCGCAATGTGCGTTCAAAGATTCGATGATTCACTGAATTCTGCAATTCACATTACTTATCGCATTTCGCTGCGTTCTTCATCGATGCCAGAGCCAAGAGATCCGTTGTTGAAAGTTTTGATTTATTTGCTTGTTTACTCAGAAAAAACATTATAGAAACAGAGTTAGGGGGTCCTCTGGCGGGGGCGGTCCGTGTTACGGGGCCGACTGTTCCCGCCGAGGCAACGTTTTACGTATGCCCACAGGGTTGATGAGTCGTATAACTCGGCACTGATSCCTCCGGTGTGGCCCCAGCGGATACCTTGGTACAACTTTTACTTCC

PWA, *F. oxysporum* KR094464.1 Cameroon

AGAAGTTGGGGTTTAACGGCGTGGCCGCGACGATTACCACCTATGGAGGGTTTTACTACTACGCTATGGAAGCTCGACGTGACCGCCAATCAATTTGAGGAACGCGAATTAACGCGAGTCCCAACACCAAGCTGTGCTTGAGGGTTGAAATGACGCTCGAACAGGCATGCCCGCCAGAATACTGGCGGGCGCAATGTGCGTTCAAAGATTCGATGATTCACTGAATTCTGCAATTCACATTACTTATCGCATTTTGCTGCGTTCTTCATCGATGCCAGAACCAAGAGATCCGTTGTTGAAAGTTTTGATTTATTTATGGTTTTACTCAGAAGTTACATATAGAAACAGAGTTTAGGGGTCCTCTGGCGGGCCGTCCCGTTTTACCGGGAGCGGGCTGATCCGCCGAGGCAACAAGTGGTATGTTCACAGGGGTTTGGGAGTTGTAAACTCGGTAATGATCCCTCCGCTGGTTCACCAACGGAG

PW’3M, *F. oxysporum* KF577910.1 Cameroon

TGGATCCACCTGCCCCGAGGTCACATTCAGAAGTTGGGGTTTAACGGCGTGGCCGCGACGATTACCAGAAACGAGGGTTTTACTACTACGCTATGGAAGCTCGACGTGACCGCCAATCAATTTGAGGAACGCGAATTAACGCGAGTCCCAACACCAAGCTGTGCTTGAGGGTTGAAATGACGCTCGAACAGGCATGCCCGCCAGAATACTGGCGGGCGCAATGTGCGTTCAAAGATTCGATGATTCACTGAATTCTGCAATTCACATTACTTATCGCATTTTGCTGCGTTCTTCATCGATGCCAGAACCAAGAGATCCGTTGTTGAAAGTTTTGATTTATTTATGGTTTTACTCAGAAGTTACATATAGAAACAGAGTTTAGGGGTCCTCTGGCGGGCCGTCCCGTTTTACCGGGAGCGGGCTGATCCGCCGAGGCAACAAGTGGTATGTTCACAGGGGTTTGGGAGTTGTAAACTCGGTAATGATCCCTCCGCTGGTTCACCAACGGAGACCTTGTTACGACTTTTTACTTCCA

PW’3A, *F. oxysporum* KR364593.1 Cameroon

ACTGCATCCGAGGTCACATTCAGAAGTTGGGGGTTAATGGCGAGGACGAAACCATTACCAATAACGAGGGTTTTACTACTACGCTATGGAAACTCGACGTGACCGCCAATCTGTTGAGGAACGCGAATTAGCGCGAGTCCCAACACCATGCTGAGCTTGAGGGTTGAAATGACGCTCGAACAGGCATGCCCGCCAGAATACTGGCGGGCGCAATGTGCGTTCGAAGATTCCATGATTCACTGAATTCTGCAATTCACATTACTTATCGCATTTTGCTGCCTTCTTCCTCGATGCCACAACCAATACATCCGCTGCTGAAAGCTTTGATTTATTTATGGCTTTACTCACAAGCTACATATATAAACCTAGCTTAGGGGTCCTCTGGCGGGCCGTCCCGCTTTACCGGGAGCGGGCTGATCCGSCGAGTAAACCTGTGGCATGTTCACAGGGGTTTGGGAGCTGTAAACTCGGCAATGATCCCTCCGCTGGTTCACCAACGGAGACCTTGTTACGACTTTTACTTCCACCATTGTACATTTTACTCCCA

NG1, *F. solani* FR691776.1 Nigeria

NNNNNNNNNNNNTTCTGGCGGGCATGCCTGTTCGAGCGTCATTACAACCCTCAGGCCCCCGGGTCTGGCGTTGGGGATCGGCGGAAGCCCCCTGTGGGCGCAACGCCCTCCCCCAAATACAGTGGCGGTCCCGCCGCAGCTTCCATTGTTTACTACCTAACACCTCNAAACTGGAAAGCGGCGCGGCCACGCCGTAAAACACCCAACTTCTGAATGTTGACCTCGAATCAGGTAGGAATACCCGCTGAACTTAAGCATATCAATAATACCCAGGAAAACTTAAGCATATCAATAAGCGGAGGAA

NG2, *F. oxysporum* HQ451894.1 Nigeria

NNNNNNNNCNGNNNNNTTCTGGCGGGCATGCCTGTTCGAGCGTCATTTCAACCCTCAAGCACAGCTTGGTGTTGGGACTCGCGTTAATTCGCGTTCCTCAAATTGATTGGCGGTCACGTCGAGCTTCCATAGCGTAGTAGTAAAACCCTCGTTACTGGTAATCGTCGCGGCCACGCCGTTAAACCCCAACTTCTGAATGTTGACCTCGGATCAGGTAGGAATACCCGCTGAACTTAAGCATATCAATAAGCGGAGGAA

NG3, *F. solani* JN235290.1 Nigeria

NNNNNNNNNNNNNNTTCTGGCGGGCATGCCTGTTCGAGCGTCATTACAACCCTCAGGCCCCCGGGCCTGGCGTTGGGGATCGGCGGAAGCCCCCTGTGGGCGCAACGCCGTCCCCCAAATACAGTGGCGGTCCCGCCGCAGCTTCCATTGCGTAGTAGCTAACACCTCGCAACTGGAGAGCGGCGCGGCCACGCCGTAAAACACCCAACTTCTGAATGTTGACCTCGAATCAGGTAGGAATACCCGCTGAACTTAAGCATATCAATAAGCGGAGGAA

NG4, *F. solani* HQ265432.1 Nigeria

NNNNNNNNNNNANNNTTCTGGCGGGCATGCCTGTTCGAGCGTCATTACAACCCTCAGGCCCCCGGGCCTGGCGTTGGGGATCGGCGGAAGCCCCCTGTGGGCGCAACGCCGTCCCCCAAATACAGTGGCGGTCCCGCCGCAGCTTCCATTGCGTAGTAGCTAACACCTCGCAACTGGAGAGCGGCGCGGCCACGCCGTAAAACACCCAACTTCTGAATGTTGACCTCGAATCAGGTAGGAATACCCGCTGAACTTAAGCATATCAATAAGCGGAGGAA

NG5, *F. oxysporum* HQ451894.1 Nigeria

NNNNNNNNNNNNNNNTTCTGGCGGGCATGCCTGTTCGAGCGTCATTTCAACCCTCAAGCACAGCTTGGTGTTGGGACTCGCGTTAATTCGCGTTCCTCAAATTGATTGGCGGTCACGTCGAGCTTCCATAGCGTAGTAGTAAAACCCTCGTTACTGGTAATCGTCGCGGCCACGCCGTTAAACCCCAACTTCTGAATGTTGACCTCGGATCAGGTAGGAATACCCGCTGAACTTAAGCATATCAATAAGCGGAGGAA

NG6, *F. solani* HQ265432.1 Nigeria

NNNNNNNNNNNNNNNTTCTGGCGGGCATGCCTGTTCGAGCGTCATTACAACCCTCAGGCCCCCGGGCCTGGCGTTGGGGATCGGCGGAAGCCCCCTGTGGGCGCAACGCCGTCCCCCAAATACAGTGGCGGTCCCGCCGCAGCTTCCATTGCGTAGTAGCTAACACCTCGCAACTGGAGAGCGGCGCGGCCACGCCGTAAAACACCCAACTTCTGAATGTTGACCTCGAATCAGGTAGGAATACCCGCTGAACTTAAGCATATCAATAAGCGGAGGAA

NG7, *F. solani* HQ265432.1 Nigeria

ANNNNNNNNANNNTTCTGGCGGGCATGCCTGTTCGAGCGTCATTACAACCCTCAGGCCCCCGGGCCTGGCGTTGGGGATCGGCGGAAGCCCCCTGTGGGCGCAACGCCGTCCCCCAAATACAGTGGCGGTCCCGCCGCAACTTCCATTGCGTAGTAGCTAACACCTCGCAACTGGAGAGCGGCGCGGCCACGCCGTAAAACACCCAACTTCTGAATGTTGACCTCGAATCATGTAGGAATACCCGCTGAACTTAAACATATCAATAAGCGGAGGAA

NG8, *F. oxysporum* JF807394.1 Nigeria

NNNNGNNNNNANNNTTCTGGCGGGCATGCCTGTTCGAGCGTCATTTCAACCCTCAAGCACAGCTTGGTGTTGGGACTCGCGTTAATTCGCGTTCCTCAAATTGATTGGCGGTCACGTCGAGCTTCCATAGCGTAGTAGTAAAACCCTCGTTACTGGTAATCGTCGCGGCCACGCCGTTAAACCCCAACTTCTGAATGTTGACCTCGGATCAGGTAGGAATACCCGCTGAACTTAAGCATATCAATAAGCGGAGGAA

NG9, *F. oxysporum* JF807394.1 Nigeria

NNNNNGNNNNNNNTTCTGGCGGGCATGCCTGTTCGAGCGTCATTTCAACCCTCAAGCACAGCTTGGTGTTGGGACTCGCGTTAATTCGCGTTCCTCAAATTGATTGGCGGTCACGTCGAGCTTCCATAGCGTAGTAGTAAAACCCTCGTTACTGGTAATCGTCGCGGCCACGCCGTTAAACCCCAACTTCTGAATGTTGACCTCGGATCAGGTAGGAATACCCGCTGAACTTAAGCATATCAATAAGCGGAGGAA

NG10*, F. solani* HQ265432.1 Nigeria

NNNNNNNNNNNNTTCTGGCGGGCATGCCTGTTCGAGCGTCATTACAACCCTCAGGCCCCCGGGCCTGGCGTTGGGGATCGGCGGAAGCCCCCTGTGGGCGCAACGCCGTCCCCCAAATACAGTGGCGGTCCCGCCGCAGCTTCCATTGCGTAGTAGCTAACACCTCGCAACTGGAGAGCGGCGCGGCCACGCCGTAAAACACCCAACTTCTGAATGTTGACCTCGAATCAGGTAGGAATACCCGCTGAACTTAAGCATATCATANNNCGGAGGAA

NG11, *F. solani* JQ277276.1 Nigeria

NNNNNNNNNNNNTTCTGGCGGGCATGCCTGTTCGAGCGTCATTACAACCCTCAGGCCCCCGGGCCTGGCGTTGGGGATCGGCGGAAGCCCCCTGCGGGCACAACGCCGTCCCCCAAATACAGTGGCGGTCCCGCCGCAGCTTCCATTGCGTAGTAGCTAACACCTCGCAACTGGAGAGCGGCGCGGCCACGCCGTAAAACACCCAACTTCTGAATGTTGACCTCGAATCAGGTAGGAATACCCGCTGAACTTAAGCATATCATAAAGGCGGAGGT

NG12, *F. chlamydosporum* EU556725.1 Nigeria

NNNNNNNNNNNANNATTCTGGCGGGCATGCCTGTTCGAGCGTCATTTCAACCCTCAAGCCCCCGGGTTTGGTGTTGGGGATCGGGCTGTACTCCAGCCCGGCCCCGAAATCTAGTGGCGGTCTCGCTGCAGCCTCCATTGCGTAGTAGCTAACACCTCGCAACTGGAACGCGGCGCGGCCAAGCCGTTAAACCCCCAACTTCTGAATGTTGACCTCGGATCAGGTAGGAATACCCGCTGAACTTAAGCATATCAATAAAGCGGAGGAA

13, *F. oxysporum* JF807394.1 Nigeria

NNNNNNNNNNNANNNTTCTGGCGGGCATGCCTGTTCGAGCGTCATTTCAACCCTCAAGCACAGCTTGGTGTTGGGACTCGCGTTAATTCGCGTTCCTCAAATTGATTGGCGGTCACGTCGAGCTTCCATAGCGTAGTAGTAAAACCCTCGTTACTGGTAATCGTCGCGGCCACGCCGTTAAACCCCAACTTCTGAATGTTGACCTCGGATCAGGTAGGAATACCCGCTGAACTTAAGCATATCAATAAGCGGAGGAA

NG13, *Fusarium chlamydosporum* FJ426391.1 Nigeria

NNNNTNNNNNCNNCNNNNTTTCTGGNGGGCATGCCTGTTCGAGCGTCATTTCAACCCTCAAGCTCAGCTTGGTGTTGGGACTCGCGGTAACCCGCGTTCCCCAAATCGATTGGCGGTCACGTCGAGCTTCCATAGCGTAGTAATCATACACCTCGTTACTGGTAATCGTCGCGGCCACGCCGTAAAACCCCAACTTCTGAATGTTGACCTCGGATCAGGTAGGAATACCCGCTGAACTTAAGCATATCAATAAGCGGAGGAATCTA

NG14, *Fusarium nelsonii* FJ426391.1 Nigeria

NNNNNNNNNNNNNTTCTGGCGGGCATGCCTGTTCGAGCGTCATTTCAACCCTCAAGCCCCCGGGTTTGGTGTTGGGGATCGGGCTGTACTCCAGCCCGGCCCCGAAATCTAGTGGCGGTCTCGCTGCAGCCTCCATTGCGTAGTAGCTAACACCTCGCAACTGGAACGCGGCGCGGCCAAGCCGTTAAACCCCCAACTTCTGAATGTTGACCTCGGATCAGGTAGGAATACCCGCTGAACTTAAGCATATCAATAAGCGGAGGAA

**Supplementary material: Figure S1: Genetic sequence of characterized *F. oxysporum* strains and secondary pathogens**
