# Supplementary material for: Molecular Identification of Secreted Effector Genes Involved in African Fusarium oxysporum f.sp. elaeidis Strains Pathogenesis During Screening Nigerian Susceptible and Tolerant Oil Palm (Elaeis guineensis Jacq.) Genotypes
Source: Front Cell Infect Microbiol. 2020 Oct 6;10:552394. doi: 10.3389/fcimb.2020.552394 (PMC7573130; doi:10.3389/fcimb.2020.552394)
Supplement: Supplementary file 2 [file Data_Sheet_2.docx]

10 20 30 40 50 60 70 80

....|....|....|....|....|....|....|....|....|....|....|....|....|....|....|....|

**OXY_1** **-----------------RRGKLQLFSDPCSYLTISLQFAKNRDSAKKTLKAKKEVILAAGAVHTPQILQVSGIGDSALLS**

**OXY_2** **---------------SCNSSRTLVHIRFLCSLPRTGTAPRRLSRRRRRSSLRLEPFTPLRSCRFPALVTQLYSPASMSLS**

**OXY_3** **--------------------KLQLFSDPCSYLTISLQFAKNRDSAKKTLKAKKEVILAAGAVHTPQILQVSGIGDSALLS**

**OXY_4** **-------------------------------------------SAKKTLKAKKEVILAAGAVHTPQILQVSGIGDSALLS**

**OXY_5** **-------------------DKRAIKYVEPWYQSPKVKRDSQPHTMQSIWAGQPVALLAWLQDNEPETLEKA--RYIFMVK**

**OXY_6** **FGKKRSVRA-NGVEVSCNSSRTLVHIRFLCSLPRTGTAPRRLSRRRRRSSLRLEPFTPLRSCRFPALVTQLYSPASMSLS**

**OXY_7** **--NNSSVREGYGVEVSCNSSRTLVHIRFLCSLPRTGTAPRRLSRRRRRSSLRLEPFTPLRSCRFPALVTQLYSPASMSLS**

**OXY_9** **---------------YCRYYWHLIDRSWHWSLFSAGKTHSWHYFNKSSLKICTMVSITIRKNSTNHAINLG--RPTCRPT**

90 100 110 120 130 140 150 160

....|....|....|....|....|....|....|....|....|....|....|....|....|....|....|....|

**OXY_1** **SIDVPVVVDLPAVG--QNFHDHVLLAVVSTSKSFPAIGNYRRLTLLSRCT------YSRQQPYQKRHFRCGARISAKEGT**

**OXY_2** **SLISLLLVRTSTIMSYSPWAQVSHFLQETTGGRCLVDAPIQGSNLTKNAT------FAAEARAEYDQQKKGP-LTSPTAD**

**OXY_3** **SIDVPVVVDLPAVG--QNFHDHVFLAVVSTSKSFPAIGKYRRLTLLSRCT------YSRQQPYQKRHFRCGARISAKEGT**

**OXY_4** **SIDVPVVVDLPAVG--QNFHDHVFLAVVSTSKSFPAIGKYRRLTLLSRCT------YSRQQPYQKRHFRCGARISAKEGT**

**OXY_5** **DLIRFYLTG-------EATQELTDISGTNLINVRDRCYDNELLAFWGGLS------WKDKLPPIKLSTDCCGRITEEIAA**

**OXY_6** **SLISLLLVRTSTIMSYSPWAQVSHFLQETTGGRCLVDAPIQGSNLTKNAT------FAAEARAEYDQQKKGP-LTSPTAD**

**OXY_7** **SLISLLLVRTSTIMSYSPWAQVSHFLQETTGGRCLVDAPIQGSNLTKNAT------FAAEARAEYDQQKKGP-LTSPTAD**

**OXY_9** **RLATRTNTGKGALHLYGERSDPLLFDGSHTRADRHLRYLDRTRSLLRQTTRFLVRLSEDKLPPIKLSTDCCGRITEEIAA**

170 180 190

....|....|....|....|....|....|....

**OXY_1** **FN-FTYRFPSLPSAFQLHKRCF------------**

**OXY_2** **FL-LFLPLSNYTSAAS------------------**

**OXY_3** **FN-FTYRFPSLPSAFQLHKRCF------------**

**OXY_4** **FN-FTYRFPSLPSAFQLHKRCF------------**

**OXY_5** **LTGLKAGTPVSGGIFDISASSLASVIPQQVALPS**

**OXY_6** **FL-LFLPLSNYTSAASEF----------------**

**OXY_7** **FL-LFLPLSNYTSAASEFH---------------**

**OXY_9** **LTGLKAGTPVSGGIFDISASSLASV---------**

**Supplementary Material: Figure S2: Sequence of putative virulent effector genes from strains of *F. oxysporum* f.sp. *elaeidis* 4 and CRT during screening tolerant and susceptible Nigerian oil palm genotypes**
