# Supplementary material for: Molecular Identification of Secreted Effector Genes Involved in African Fusarium oxysporum f.sp. elaeidis Strains Pathogenesis During Screening Nigerian Susceptible and Tolerant Oil Palm (Elaeis guineensis Jacq.) Genotypes
Source: Front Cell Infect Microbiol. 2020 Oct 6;10:552394. doi: 10.3389/fcimb.2020.552394 (PMC7573130; doi:10.3389/fcimb.2020.552394)
Supplement: Supplementary file 3 [file Data_Sheet_3.docx]

510 520 530 540 550 560 570 580 590 600

....|....|....|....|....|....|....|....|....|....|....|....|....|....|....|....|....|....|....|....|

**OXY_5** **GCGTGCCATCAAATATGTTGAACCTT---GGTATCAATCACCTAAAGTCAAAAGGGATTCTCAACCCCACACGATGCAATCAATTTGGGCAGGCCAACCT**

**OXY_6** **T..GA..C.TGTTC..A.CTG..GA.TTCTT.GCAGT.TG..A.G.ACAGGG.CA.CGC.AAG.AGA.T.T.A.G..G.AG..GGA..T..TC.TTG.GG**

**OXY_4** **-----------------------------------------------------CA.CGC.AAG.AGA.T.T.A.G..G.AG..GGA..T..TC.TTG.GG**

**OXY_3** **T..GA..C.TGTTC..A.CTG..GA.TTCTT.GCAGT.TG..A.G.A.AGGG.CA.CGC.AAG.AGA.T.T.A.G..G.AG..GGA..T..TC.TTG.GG**

**OXY_2** **T..GA..C.TGTTC..A.CTG..GA.TTCTT.GCAGT.TG..A.G.ACAGGG.CA.CGC.AAG.AGA.T.T.A.G..G.AG..GGA..T..TC.TTG.GG**

**OXY_9** **T..A...T.A.......C.....AA.---.................T..G....T..AAT..T...TA.A..C...........................**

**OXY_7** **T..GA..C.TGTTC..A.CTG..GA.TTCTT.GCAGT.TG..A.G.ACAGGG.CA.CGC.AAG.AGA.T.T.A.G..G.AG..GGA..T..TC.TTG.GG**

610 620 630 640 650 660 670 680 690 700

....|....|....|....|....|....|....|....|....|....|....|....|....|....|....|....|....|....|....|....|

**OXY_5** **GTCGCCCTACTCGCTTGGCTACAAGATAATGAACCTGAAACACTGGAAAAGGCGCGTTACATCTTTATGGTGAAAGATCTGATCCGCTTTTATTTGACGG**

**OXY_6** **C.G.AG.CGT..A.ACCC..CAG.TCCTGCAGGTT.CCGG..T...TG.CTCA..T....-...CC.GCA.CG.T.TC.CTG..GT.G..G..C.CC.T.**

**OXY_4** **C.G.AG.CGT..A.ACCC..CAG.TCCTGCAGGTT.CCGG..T...TG.CTCA..T....-...CC.GCA.CG.T.TC.CTG..GT.G..G..C.CC.T.**

**OXY_3** **C.G.AG.CGT..A.ACCC..CAG.TCCTGCAGGTT.CCGG..T...TG.CTCA..T....-...CC.GCA.CG.T.TC.CTG..GT.G..G..C.CC.T.**

**OXY_2** **C.G.AG.CGT..A.ACCC..CAG.TCCTGCAGGTT.CCGG..T...TG.CTCA..T....-...CC.GCA.CG.T.TC.CTG..GT.G..G..C.CC.T.**

**OXY_9** **....................................................................................................**

**OXY_7** **C.G.AG.CGT..A.ACCC..CAG.TCCTGCAGGTT.CCGG..T...TG.CTCA..T....-...CC.GCA.CG.T.TC.CTG..GT.G..G..C.CC.T.**

710 720 730 740 750 760 770 780 790 800

....|....|....|....|....|....|....|....|....|....|....|....|....|....|....|....|....|....|....|....|

**OXY_5** **GTGA-AGCCACACAAGAGCTGACCGACATCTCAGGTACTAACTTGATTA-ACGTACGCGATCGTTGCTACGACAATGAACTACTCGCTTTTTGGGGCGGA**

**OXY_6** **C..TTG.T..G.ACTTCCAC..T.-.TG...T.CTCG.CGTGG...GC.C.A...A.TC..--..C..G.A.T.GGA......CG.AGGC.GAC.TT.CT**

**OXY_4** **C..TTG.T..G.ACTTCCAC..T.-.TG...TTCTCG.CGTGG...GC.C.A...A.TC..--.CC..G.A.T.GGA..G...CG.AGGC.GAC.TT.CT**

**OXY_3** **C..TTG.T..G.ACTTCCAC..T.-.TG...TTCTCG.CGTGG...GC.C.A...A.TC..--.CC..G.A.T.GGA..G...CG.AGGC.GAC.TT.CT**

**OXY_2** **C..TTG.T..G.ACTTCCAC..T.-.TG...T.CTCG.CGTGG...GC.C.A...A.TC..--..C..G.A.T.GGA......CG.AGGC.GAC.TT.CT**

**OXY_9** **....-............................................-............................................TT...-**

**OXY_7** **C..TTG.T..G.ACTTCCAC..T.-.TG...T.CTCG.CGTGG...GC.C.A...A.TC..--..C..G.A.T.GGA......CG.AGGC.GAC.TT.CT**

810 820 830 840 850 860 870 880 890 900

....|....|....|....|....|....|....|....|....|....|....|....|....|....|....|....|....|....|....|....|

**OXY_5** **CTATCGTGGAA---AGATAAACTACCG--CCGATCAAGTTATCCACT--GATTGTTGTGGCCGTATCACAGAAGAAATCGCTGCACTGACTGGCCTTAAA**

**OXY_6** **TAG...AT.C.CCT.TTC..GGC.G.AAC.TT.C...AAACG.....TTCGC..C..A...-.CGCGCTGA.TATG...AGCAA.AGA.GG.A....T..**

**OXY_4** **TAG...AT.C.CCT.TTC..GGC.G.AAC.TT.C...AAACG.....TTCGC..C..A...-.CGCGCTGA.TATG...AGCAA.AGA.GG.A....T..**

**OXY_3** **TAG...AT.C.CCT.TTC..GGC.G.AAC.TT.C...AAACG.....TTCGC..C..A...-.CGCGCTGA.TATG...AGCAA.AGA.GG.A....T..**

**OXY_2** **TAG...AT.C.CCT.TTC..GGC.G.AAC.TT.C...AAACG.....TTCGC..C..A...-.CGCGCTGA.TATG...AGCAA.AGA.GG.A....T..**

**OXY_9** **........AG.---.............--..................--...................................................**

**OXY_7** **TAG...AT.C.CCT.TTC..GGC.G.AAC.TT.C...AAACG.....TTCGC..C..A...-.CGCGCTGA.TATG...AGCAA.AGA.GG.A....T..**

910 920 930 940 950 960 970 980 990

....|....|....|....|....|....|....|....|....|....|....|....|....|....|....|....|....|....|...

**OXY_5** **GCAGGCA---CGCCAGTTTCAGGTGGCATCTTTGATATTTCCGCTTCGTCATTG--GCTTCAGTAATTCCACAACAAGTCGCACTCCCGTCGT**

**OXY_6** **CTTCA.CTAC...TGA....CTTCTCTTC...CCGCT..C.AA..A.ACA.GC.CT.......A.T....-----------------------**

**OXY_4** **CTTCA.CTAC...TGA....CTTCTCTTC...CCGCT..C.AA..A.ACA.GC.CT......-------------------------------**

**OXY_3** **CTTCA.CTAC...TGA....CTTCTCTTC...CCGCT..C.AA..A.ACA.GC.CT......-------------------------------**

**OXY_2** **CTTCA.CTAC...TGA....CTTCTCTTC...CCGCT..C.AA..A.ACA.GC.CT......-------------------------------**

**OXY_9** **.......---............................................--.........T---------------------------**

**OXY_7** **CTTCA.CTAC...TGA....CTTCTCTTC...CCGCT..C.AA..A.ACA.GC.CT.......A.T.C.ACA.--------------------**


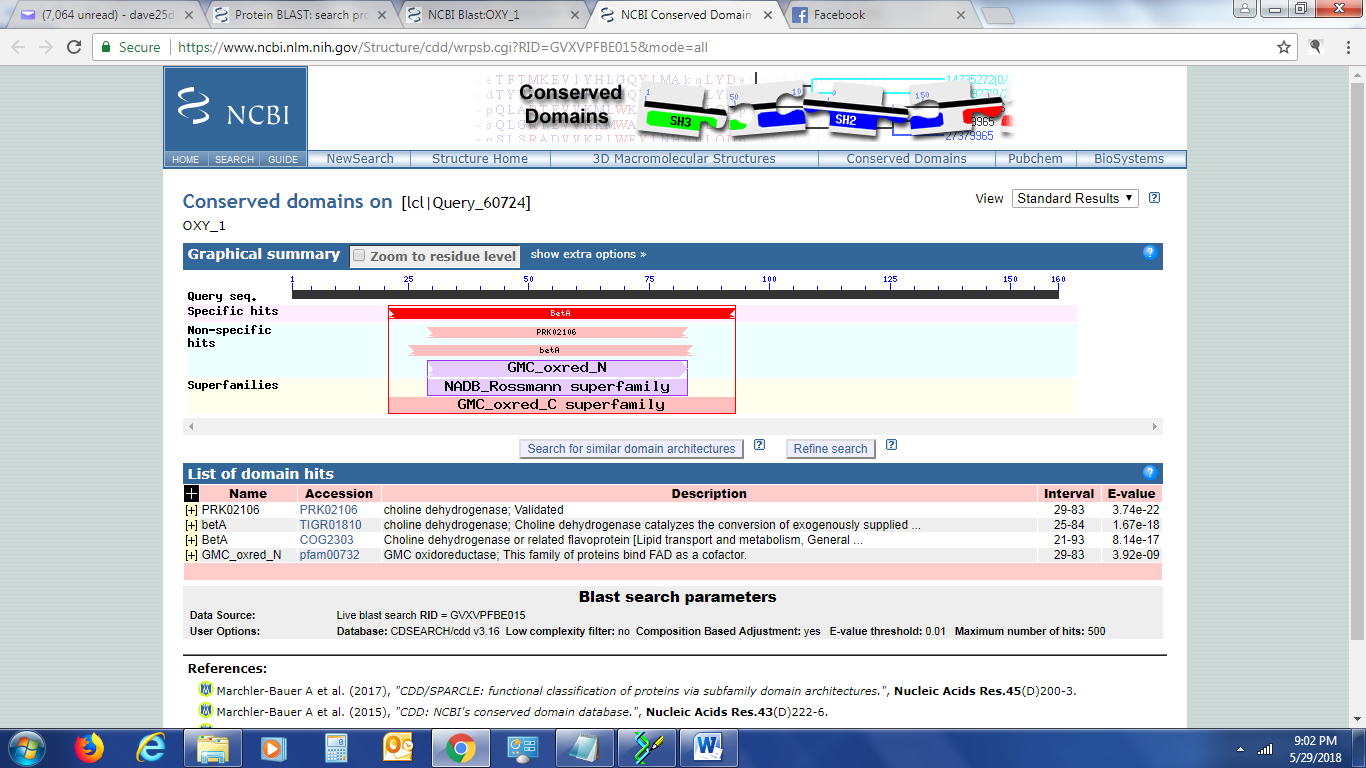


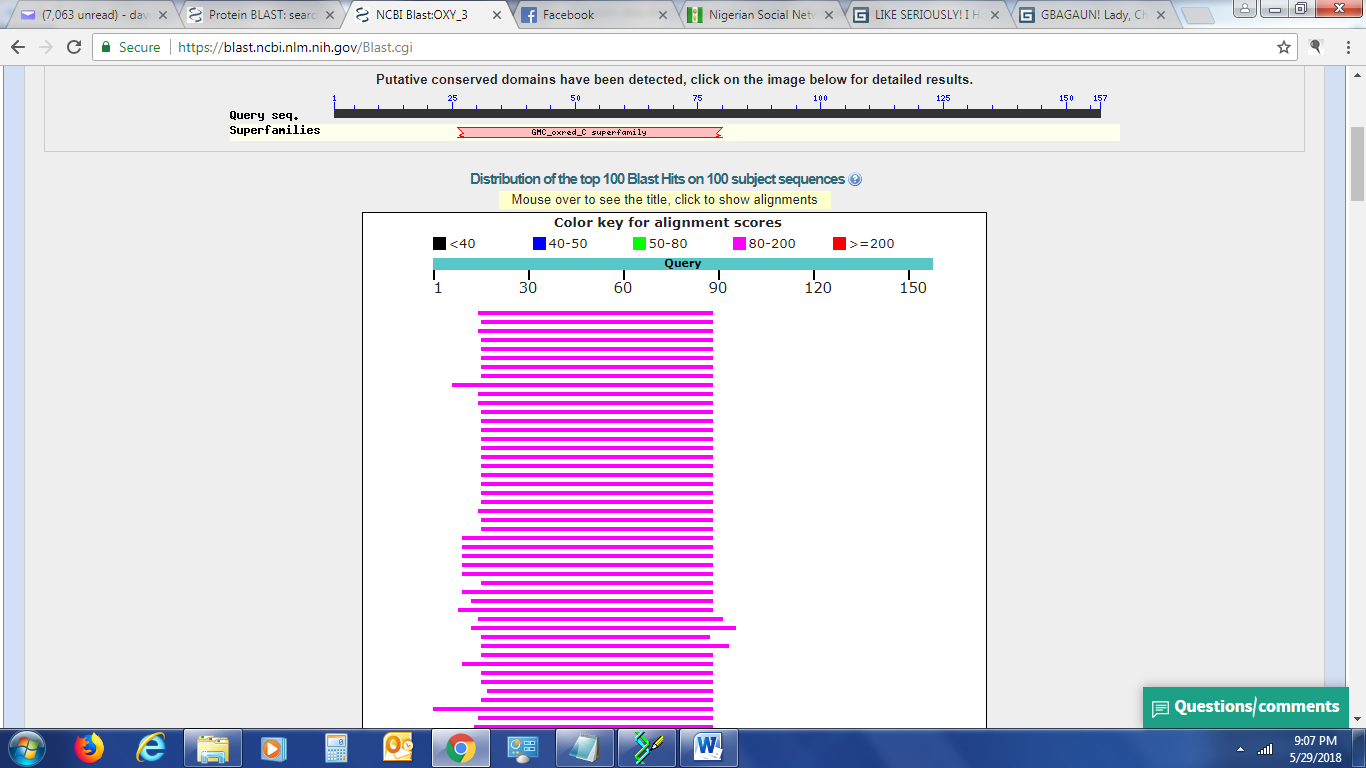


**Supplementary Material: Figure S3: Sequence of putative virulent effector genes from strains of *F. oxysporum* f.sp. *elaeidis* 4 and CRT during screening tolerant and susceptible Nigerian oil palm genotypes.**
