# Supplementary material for: Molecular Identification of Secreted Effector Genes Involved in African Fusarium oxysporum f.sp. elaeidis Strains Pathogenesis During Screening Nigerian Susceptible and Tolerant Oil Palm (Elaeis guineensis Jacq.) Genotypes
Source: Front Cell Infect Microbiol. 2020 Oct 6;10:552394. doi: 10.3389/fcimb.2020.552394 (PMC7573130; doi:10.3389/fcimb.2020.552394)
Supplement: Supplementary file 4 [file Data_Sheet_4.docx]

**
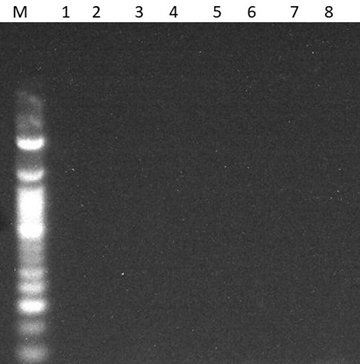
**

**1000 bp ladder**

Electropherogram of PCR product of 8 oil palm genotype using specific primer pair, P1 and P2. Lane 1-5 (genotype 120, susceptible), (genotype 2211, susceptible), (genotype 3023, susceptible), (genotype 2478, susceptible), (genotype 3456, susceptible), Lane 6-7 (genotype 1621, tolerant) (genotype 1723, tolerant) and lane 8 (genotype 4189, tolerant.

**
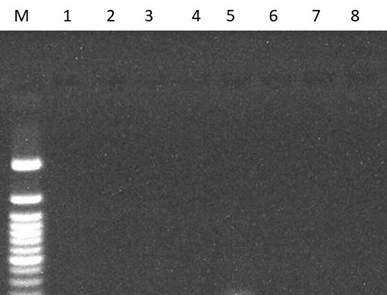
**

**1000 bp ladder**

Electropherogram of PCR product of 8 oil palm genotype using specific primer pair, PR-1F and PR-1R. Lane 1-5 (genotype 120, susceptible), (genotype 2211, susceptible), (genotype 3023, susceptible), (genotype 2478, susceptible), (genotype 3456, susceptible), Lane 6-7 (genotype 1621, tolerant) (genotype 1723, tolerant) and lane 8 (genotype 4189, tolerant.

**Supplementary Material: Figure S4: Electropherogram of PCR amplified products of oil palm genotypes using defence gene primers**
