# Supplementary material for: Molecular Identification of Secreted Effector Genes Involved in African Fusarium oxysporum f.sp. elaeidis Strains Pathogenesis During Screening Nigerian Susceptible and Tolerant Oil Palm (Elaeis guineensis Jacq.) Genotypes
Source: Front Cell Infect Microbiol. 2020 Oct 6;10:552394. doi: 10.3389/fcimb.2020.552394 (PMC7573130; doi:10.3389/fcimb.2020.552394)
Supplement: Supplementary file 5 [file Data_Sheet_5.docx]

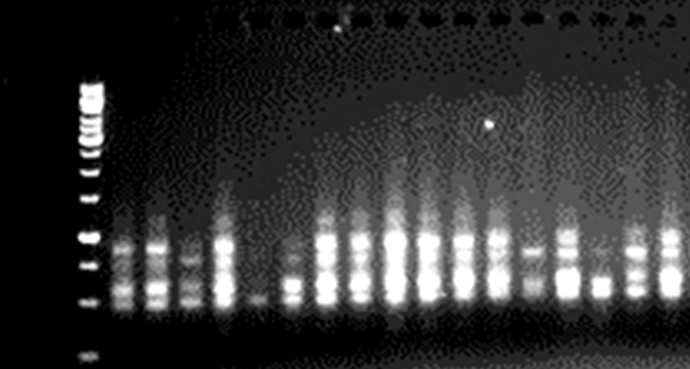


M 1 2 3 4 5 6 7 8 9 10 11 12 13 14 15 16 17

**Plate 3**: PCR amplification profile of seventeen *F. oxysporum* f.sp. *elaeidis* DNA fragments using ISSR markers (ISSR 858). M=100 bp step DNA ladder, 1-17. *(F. oxysporum* f.sp. *elaeidis* (1); *F. oxysporum* f.sp. *elaeidis* (4); *F. oxysporum* f.sp. *elaeidis* (PW11A); *F. oxysporum* f.sp. *elaeidis* (PW11M), *F. oxysporum* f.sp. *elaeidis* (PW’3M); *F. oxysporum* f.sp. *elaeidis* (PW’3A); *F. oxysporum* f.sp. *elaeidis* (PW49A); *F. oxysporum* f.sp. *elaeidis* (MAT’10B); *F. oxysporum* f.sp. *elaeidis* (MAT14B); *F. oxysporum* f.sp. *elaeidis* (PU’4APU); *F. oxysporum* f.sp. *elaeidis* (PWA); *F. oxysporum* f.sp. *elaeidis* (EK1B); *F.* *oxysporum* f.sp. *elaeidis* (MAT’9B) all from Cameroon); *F. oxysporum* f.sp. *elaeidis* (BOPP); *F. oxysporum* f.sp. *elaeidis* (CRT); *F. oxysporum* f.sp. *elaeidis* (622) all from Ghana) *F. oxysporum* f.sp. *elaeidis* (13) from Nigeria.

**Supplementary Material: Figure S5:** PCR amplification profile of *F. oxysporum* f.sp. *elaeidis* DNA fragments using ISSR markers
