# Supplementary material for: Molecular Identification of Secreted Effector Genes Involved in African Fusarium oxysporum f.sp. elaeidis Strains Pathogenesis During Screening Nigerian Susceptible and Tolerant Oil Palm (Elaeis guineensis Jacq.) Genotypes
Source: Front Cell Infect Microbiol. 2020 Oct 6;10:552394. doi: 10.3389/fcimb.2020.552394 (PMC7573130; doi:10.3389/fcimb.2020.552394)
Supplement: Supplementary file 6 [file Data_Sheet_6.docx]

**Supplementary Material: Table S1: Geographical positioning systems of coordinates of *Fusarium* infected oil palm field in Nigeria**

| **N°** | **Block** **/ Year of Plantation/**  **Location** | **Oil palm**  **Genotype** | **Symptoms** | **Geographical coordinates** **(Degrees)** | | | **Observations** |
| --- | --- | --- | --- | --- | --- | --- | --- |
|  |  |  |  | **Latitude**  **°N** | **Longitude**  **°W** | **Elevation**  **(m)** |  |
| 1 | OPC | Tenera | Acute | 5°3'22.84 | 1°55'57.962 | 150 | Yellowing of the leaves |
| 2 | NIFOR central station |  | Chronic | 5°8'28.456 | 1°54'44.323 | 170 | Stunted |
| 3 | NIFOR central station |  | Chronic | 6°15'14.42 | 0°52'35.667 | 163 | Trunk with the pointed apex |
| 4 | NIFOR central station |  | Acute | 6°15'4.635 | 0°52'21.622 | 169 | Yellowing of the leaves |
| 5 | Ajagbodudu |  | Chronic | 6°13'11.611 | 0°49'18.314 | 162 | Stunted |
| 6 | ABAK |  | Acute | 6°17'24.229 | 0°53'43.164 | 250 | Yellowing of the leaves |
| 7 | ABAK |  | Chronic | 5°6'1.766 | 1°51'40.225 | 280 | Completely opened trunk |

OPC means Oil producing company

NIFOR means Nigerian Institute for Oil Palm Research
