# Supplementary material for: Molecular Identification of Secreted Effector Genes Involved in African Fusarium oxysporum f.sp. elaeidis Strains Pathogenesis During Screening Nigerian Susceptible and Tolerant Oil Palm (Elaeis guineensis Jacq.) Genotypes
Source: Front Cell Infect Microbiol. 2020 Oct 6;10:552394. doi: 10.3389/fcimb.2020.552394 (PMC7573130; doi:10.3389/fcimb.2020.552394)
Supplement: Supplementary file 7 [file Data_Sheet_7.docx]

**Supplementary Material: Table S2:** Identified *Fusarium* strains, secondary pathogen, and percentage homology

| **S/NO** | **CODE NUMBER** | **FUNGUS ISOLATED** | **PERCENTAGE MATCH (%)** | **ACCESSION NUMBER** | **COUNTRIES** |
| --- | --- | --- | --- | --- | --- |
| 1 | 46 | *F. solani* | 100 | KJ863521.1 | CAMEROON |
| 2 | 4 | *F. oxysporum* | 95 | AY928419.1 | CAMEROON |
| 3 | EK1B | *F. oxysporum* | 100 | KM268692.1 | CAMEROON |
| 4 | PU’4APU | *F. oxysporum* | 100 | KF577910.1 | CAMEROON |
| 5 | 42 | *F. proliferatum* | 100 | KR350649.1 | CAMEROON |
| 6 | PW11A | *F. oxysporum* | 100 | EU364854.1 | CAMEROON |
| 7 | PW03 | *F. oxysporum* | 100 | KR364596.1 | CAMEROON |
| 8 | PW’B | *F. solani* | 100 | KC907714.1 | CAMEROON |
| 9 | PW9B | *F. verticillioides* | 100 | KF624791.1 | CAMEROON |
| 10 | MAT 15M | *F. equiseti* | 100 | KR364597.1 | CAMEROON |
| 11 | PW’1A | *F. solani* | 100 | KF060154.1 | CAMEROON |
| 12 | MAT 14B | *F. oxysporum* | 100 | KR364596.1 | CAMEROON |
| 13 | MA02 | *F. verticillioides* | 100 | KR183784.1 | CAMEROON |
| 14 | PW10B | *F. oxysporum* | 100 | KT898585.1 | CAMEROON |
| 15 | MAT’3M | *F. equiseti* | 100 | JQ936180.1 | CAMEROON |
| 16 | 29 | *F. equiseti* | 100 | JQ936180.1 | CAMEROON |
| 17 | PW7B | *F. equiseti* | 100 | KR094457.1 | CAMEROON |
| 18 | MAT’4’A | *Penicillium simplicissimum* | 100 | KM613146.1 | CAMEROON |
| 19 | MAT16M | *F. equiseti* | 100 | KR094457.1 | CAMEROON |
| 20 | PW’3A2 | *Fomes fomentarius* | 100 | EF155498.1 | CAMEROON |
| 21 | PW12M | *F. verticillioides* | 100 | KR183784.1 | CAMEROON |
| 22 | PW11M | *F. oxysporum* | 100 | KF577910.1 | CAMEROON |
| 23 | PW6M | *F. equiseti* | 100 | JQ936153.1 | CAMEROON |
| 24 | PW8MP | *F. equiseti* | 100 | KM246253.1 | CAMEROON |
| 25 | PW’2MA | *F. equiseti* | 100 | KP942954.1 | CAMEROON |
| 26 | 1 | *F. oxysporum* | 100 | KP942906.1 | CAMEROON |
| 27 | 44 | *F. equiseti* | 100 | KR025562.1 | CAMEROON |
| 28 | EKI | *F. oxysporum* | 100 | KU872840.1 | CAMEROON |
| 29 | MAT’4B | *F. solani* | 100 | FJ719812.1 | CAMEROON |
| 30 | MAT’9B | *F. oxysporum* | 100 | KR364584.1 | CAMEROON |

**Supporting Information: Table S2 (Contd):** Identified *Fusarium* strains, secondary pathogen, and percentage homology

| **S/NO** | **CODE NUMBER** | **FUNGUS ISOLATED** | **PERCENTAGE MATCH (%)** | **ACCESSION NUMBER** | **COUNTRIES** |
| --- | --- | --- | --- | --- | --- |
| 31 | MAT’10B | *F. oxysporum* | 100 | KR364587.1 | CAMEROON |
| 32 | PW’3M | *F. oxysporum* | 100 | KF577910.1 | CAMEROON |
| 33 | PW’3A | *F. oxysporum* | 100 | KR364593.1 | CAMEROON |
| 34 | PW49A | *F. oxysporum* | 100 | KU872818.1 | CAMEROON |
| 35 | CRT | *F. oxysporum* | 100 | KR094464.1 | GHANA |
| 36 | 205 | *F. proliferatum* | 100 | KC254038.1 | GHANA |
| 37 | 583 | *F. equiseti* | 100 | KR047064.1 | GHANA |
| 38 | 622 | *F. oxysporum* | 100 | KR094464.1 | GHANA |
| 39 | BOPP | *F. oxysporum* | 100 | KR364584.1 | GHANA |
| 40 | SP | *F. solani* | 100 | FJ719812.1 | GHANA |
| 41 | PWA | *F. oxysporum* | 100 | KR094464.1 | CAMEROON |
| 42 | 13 | *F. oxysporum* | 100 | JF807394.1 | NIGERIA |
| 43 | PW11B | *F. equiseti* | 100 | KR025562.1 | CAMEROON |
| 44 | PW8W | *F. equiseti* | 100 | KR047064.1 | CAMEROON |
| 45 | NG1 | *F. solani* | 96 | [FR691776.1](http://www.ncbi.nlm.nih.gov/nucleotide/319801169?report=genbank&log$=nucltop&blast_rank=28&RID=ZGEX13SR013) | NIGERIA |
| 46 | NG2 | *F. oxysporum* | 100 | [HQ451894.1](http://www.ncbi.nlm.nih.gov/nucleotide/325662669?report=genbank&log$=nucltop&blast_rank=11&RID=ZGF9071Z013) | NIGERIA |
| 47 | NG3 | *F. solani* | 99 | [JN235290.1](http://www.ncbi.nlm.nih.gov/nucleotide/365733785?report=genbank&log$=nucltop&blast_rank=2&RID=ZGFF1H63011) | NIGERIA |
| 48 | NG4 | *F. solani* | 99 | [HQ265432.1](http://www.ncbi.nlm.nih.gov/nucleotide/315111141?report=genbank&log$=nucltop&blast_rank=65&RID=ZGFK0VKW011) | NIGERIA |
| 49 | NG5 | *F. oxysporum* | 100 | [HQ451894.1](http://www.ncbi.nlm.nih.gov/nucleotide/325662669?report=genbank&log$=nucltop&blast_rank=11&RID=ZGG8DUJJ013) | NIGERIA |
| 50 | NG6 | *F. solani* | 99 | [HQ265432.1](http://www.ncbi.nlm.nih.gov/nucleotide/315111141?report=genbank&log$=nucltop&blast_rank=65&RID=ZGGCEUHH011) | NIGERIA |
| 51 | NG7 | *F. solani* | 98 | [HQ265432.1](http://www.ncbi.nlm.nih.gov/nucleotide/315111141?report=genbank&log$=nucltop&blast_rank=66&RID=ZGGG2F70011) | NIGERIA |
| 52 | NG8 | *F. oxysporum* | 100 | [JF807394.1](http://www.ncbi.nlm.nih.gov/nucleotide/356582660?report=genbank&log$=nucltop&blast_rank=4&RID=ZGGR34SA011) | NIGERIA |
| 53 | NG9 | *F. oxysporum* | 100 | [JF807394.1](http://www.ncbi.nlm.nih.gov/nucleotide/356582660?report=genbank&log$=nucltop&blast_rank=4&RID=ZGJS2UPP013) | NIGERIA |
| 54 | NG10 | *F. solani* | 99 | [HQ265432.1](http://www.ncbi.nlm.nih.gov/nucleotide/315111141?report=genbank&log$=nucltop&blast_rank=65&RID=ZGJV6BMM013) | NIGERIA |
| 55 | NG11 | *F. solani* | 99 | [JQ277276.1](http://www.ncbi.nlm.nih.gov/nucleotide/380863939?report=genbank&log$=nucltop&blast_rank=1&RID=ZGJYGGR6013) | NIGERIA |
| 56 | NG12 | *F. chlamydosporum* | 97 | [EU556725.1](http://www.ncbi.nlm.nih.gov/nucleotide/170676838?report=genbank&log$=nucltop&blast_rank=51&RID=ZGK1E57G01N) | NIGERIA |
| 57 | NG13 | *F. chlamydosporum* | 100 | [FJ426391.1](http://www.ncbi.nlm.nih.gov/nucleotide/221267444?report=genbank&log$=nucltop&blast_rank=1&RID=ZGKG9UFS013) | NIGERIA |
| 58 | NG14 | *F. nelsonii* | 100 | [GQ505434.1](http://www.ncbi.nlm.nih.gov/nucleotide/262476204?report=genbank&log$=nucltop&blast_rank=31&RID=ZGKM7YBJ011) | NIGERIA |
