# Supplementary material for: Molecular Identification of Secreted Effector Genes Involved in African Fusarium oxysporum f.sp. elaeidis Strains Pathogenesis During Screening Nigerian Susceptible and Tolerant Oil Palm (Elaeis guineensis Jacq.) Genotypes
Source: Front Cell Infect Microbiol. 2020 Oct 6;10:552394. doi: 10.3389/fcimb.2020.552394 (PMC7573130; doi:10.3389/fcimb.2020.552394)
Supplement: Supplementary file 8 [file Data_Sheet_8.docx]

**Supplementary Material: Table S3: Summary of incidence score of Fusarium wilt disease of oil palm genotypes**

**STRAIN (1)**

| **GENOTYPES** | **BLOCK 1** | | | |  | **BLOCK 2** | | | |  | **BLOCK 3** | | | |
| --- | --- | --- | --- | --- | --- | --- | --- | --- | --- | --- | --- | --- | --- | --- |
|  | **H** | **LS** | **L&R** | **RS** |  | **H** | **LS** | **L&R** | **RS** |  | **H** | **LS** | **L&R** | **RS** |
| **1** | 3 | 0 | 0 | 6 |  | 9 | 0 | 0 | 0 |  | 8 | 0 | 0 | 1 |
| **2** | 8 | 0 | 0 | 1 |  | 9 | 0 | 0 | 0 |  | 7 | 1 | 0 | 1 |
| **3** | 6 | 1 | 0 | 2 |  | 8 | 0 | 0 | 1 |  | 9 | 0 | 0 | 0 |
| **4** | 7 | 0 | 0 | 2 |  | 8 | 0 | 0 | 1 |  | 7 | 0 | 0 | 2 |
| **5** | 7 | 2 | 0 | 0 |  | 9 | 0 | 0 | 0 |  | 8 | 0 | 0 | 1 |
| **6** | 7 | 2 | 0 | 0 |  | 7 | 2 | 0 | 0 |  | 7 | 2 | 0 | 0 |
| **7** | 8 | 1 | 0 | 0 |  | 8 | 1 | 0 | 0 |  | 8 | 1 | 0 | 0 |
| **8** | 12 | 2 | 0 | 0 |  | 10 | 4 | 0 | 0 |  | 13 | 1 | 0 | 0 |

**STRAIN (4)**

| **GENOTYPES** | **BLOCK 1** | | | |  | **BLOCK 2** | | | |  | **BLOCK 3** | | | |
| --- | --- | --- | --- | --- | --- | --- | --- | --- | --- | --- | --- | --- | --- | --- |
|  | **H** | **LS** | **L&R** | **RS** |  | **H** | **LS** | **L&R** | **RS** |  | **H** | **LS** | **L&R** | **RS** |
| **1** | 1 | 0 | 5 | 2 |  | 0 | 0 | 7 | 2 |  | 1 | 0 | 8 | 0 |
| **2** | 0 | 0 | 7 | 2 |  | 3 | 0 | 6 | 0 |  | 1 | 0 | 8 | 0 |
| **3** | 0 | 0 | 9 | 0 |  | 2 | 0 | 5 | 2 |  | 1 | 0 | 8 | 0 |
| **4** | 1 | 0 | 4 | 4 |  | 1 | 0 | 6 | 2 |  | 2 | 0 | 6 | 1 |
| **5** | 0 | 0 | 7 | 2 |  | 2 | 0 | 6 | 1 |  | 1 | 0 | 8 | 0 |
| **6** | 3 | 0 | 5 | 1 |  | 3 | 0 | 5 | 1 |  | 3 | 0 | 5 | 1 |
| **7** | 2 | 2 | 2 | 3 |  | 2 | 2 | 2 | 3 |  | 2 | 2 | 2 | 3 |
| **8** | 14 | 0 | 0 | 0 |  | 11 | 3 | 0 | 0 |  | 12 | 2 | 0 | 0 |

**STRAIN (13)**

| **GENOTYPES** | **BLOCK 1** | | | |  | **BLOCK 2** | | | |  | **BLOCK 3** | | | |
| --- | --- | --- | --- | --- | --- | --- | --- | --- | --- | --- | --- | --- | --- | --- |
|  | **H** | **LS** | **L&R** | **RS** |  | **H** | **LS** | **L&R** | **RS** |  | **H** | **LS** | **L&R** | **RS** |
| **1** | 3 | 5 | 0 | 1 |  | 6 | 1 | 0 | 1 |  | 7 | 1 | 0 | 1 |
| **2** | 3 | 3 | 0 | 3 |  | 4 | 5 | 0 | 0 |  | 8 | 1 | 0 | 0 |
| **3** | 6 | 3 | 0 | 0 |  | 5 | 4 | 0 | 0 |  | 4 | 4 | 0 | 1 |
| **4** | 6 | 3 | 0 | 0 |  | 6 | 3 | 0 | 0 |  | 8 | 1 | 0 | 0 |
| **5** | 8 | 1 | 0 | 0 |  | 8 | 0 | 0 | 1 |  | 8 | 1 | 0 | 0 |
| **6** | 8 | 1 | 0 | 0 |  | 8 | 1 | 0 | 0 |  | 8 | 1 | 0 | 0 |
| **7** | 8 | 1 | 0 | 0 |  | 8 | 1 | 0 | 0 |  | 8 | 1 | 0 | 0 |
| **8** | 11 | 3 | 0 | 0 |  | 11 | 3 | 0 | 0 |  | 11 | 3 | 0 | 0 |

**STRAIN (CRT)**

| **GENOTYPES** | **BLOCK 1** | | | |  | **BLOCK 2** | | | |  | **BLOCK 3** | | | |
| --- | --- | --- | --- | --- | --- | --- | --- | --- | --- | --- | --- | --- | --- | --- |
|  | **H** | **LS** | **L&R** | **RS** |  | **H** | **LS** | **L&R** | **RS** |  | **H** | **LS** | **L&R** | **RS** |
| **1** | 0 | 2 | 7 | 0 |  | 1 | 2 | 4 | 2 |  | 8 | 1 | 0 | 0 |
| **2** | 0 | 4 | 4 | 1 |  | 2 | 4 | 2 | 1 |  | 4 | 0 | 3 | 2 |
| **3** | 1 | 3 | 5 | 0 |  | 2 | 2 | 4 | 1 |  | 2 | 2 | 2 | 3 |
| **4** | 4 | 3 | 2 | 0 |  | 4 | 5 | 0 | 0 |  | 5 | 0 | 2 | 2 |
| **5** | 2 | 3 | 4 | 0 |  | 5 | 1 | 3 | 0 |  | 5 | 2 | 1 | 1 |
| **6** | 3 | 0 | 3 | 0 |  | 3 | 0 | 3 | 0 |  | 3 | 0 | 3 | 0 |
| **7** | 4 | 0 | 4 | 1 |  | 4 | 0 | 4 | 1 |  | 4 | 0 | 4 | 1 |
| **8** | 11 | 3 | 0 | 0 |  | 12 | 2 | 0 | 0 |  | 12 | 2 | 0 | 0 |

**GRAND SUMMARY OF LEAF AND ROOT FINAL SCORE INCIDENCE OF FUSARIUM WILT DISEASE ON OIL PALM GENOTYPES**

| **STRAINS** | **GENOTYPES** | **H** | **LS** | **L&R** | **RS** |
| --- | --- | --- | --- | --- | --- |
| STRAIN (1) | 1 | 20 | 0 | 0 | 7 |
|  | 2 | 24 | 1 | 0 | 2 |
|  | 3 | 23 | 1 | 0 | 3 |
|  | 4 | 22 | 0 | 0 | 5 |
|  | 5 | 24 | 2 | 0 | 1 |
|  | 6 | 21 | 6 | 0 | 0 |
|  | 7 | 24 | 3 | 0 | 0 |
|  | 8 | 35 | 7 | 0 | 0 |
| TOTAL= 193 TOTAL= 20 TOTAL= 0 TOTAL= 18 | | | | | |
| STRAIN (4) | 1 | 2 | 0 | 20 | 4 |
|  | 2 | 4 | 0 | 21 | 2 |
|  | 3 | 3 | 0 | 22 | 2 |
|  | 4 | 4 | 0 | 16 | 7 |
|  | 5 | 3 | 0 | 21 | 3 |
|  | 6 | 9 | 0 | 15 | 3 |
|  | 7 | 4 | 6 | 6 | 9 |
|  | 8 | 37 | 0 | 0 | 0 |
| TOTAL= 66 TOTAL= 6 TOTAL= 121 TOTAL= 30 | | | | | |
| STRAIN (13) | 1 | 16 | 7 | 0 | 3 |
|  | 2 | 15 | 9 | 0 | 3 |
|  | 3 | 15 | 11 | 0 | 1 |
|  | 4 | 20 | 7 | 0 | 0 |
|  | 5 | 24 | 2 | 0 | 1 |
|  | 6 | 24 | 3 | 0 | 0 |
|  | 7 | 24 | 3 | 0 | 0 |
|  | 8 | 33 | 9 | 0 | 0 |
| TOTAL= 171 TOTAL= 51 TOTAL= 0 TOTAL= 8 | | | | | |
| STRAIN (CRT) | 1 | 9 | 5 | 11 | 2 |
|  | 2 | 6 | 8 | 9 | 4 |
|  | 3 | 5 | 7 | 11 | 4 |
|  | 4 | 13 | 8 | 4 | 2 |
|  | 5 | 12 | 6 | 8 | 1 |
|  | 6 | 9 | 0 | 9 | 0 |
|  | 7 | 12 | 0 | 12 | 3 |
|  | 8 | 35 | 7 | 0 | 0 |
| TOTAL= 101 TOTAL= 41 TOTAL= 64 TOTAL= 16 | | | | | |

**GRAND SUMMARY OF LEAF AND ROOT FINAL SCORE INCIDENCE OF FUSARIUM WILT DISEASE ON OIL PALM GENOTYPES**

| **STRAINS** | **H** | **LS** | **L&R** | **RS** |
| --- | --- | --- | --- | --- |
| STRAIN (1) | 193 | 20 | 0 | 18 |
| STRAIN (4) | 66 | 6 | 121 | 30 |
| STRAIN (13) | 171 | 51 | 0 | 8 |
| STRAIN (CRT) | 101 | 41 | 64 | 16 |

**KEY**:

H== HEALTHY

LS== LEAF SYMPTOM ONLY

L&R== LEAF AND ROOT SYMPTOM

RS==== ROOT SYMPTOM ONLY

THE MANIFICIENT MANLLLLLL
