# Supplementary material for: Molecular Identification of Secreted Effector Genes Involved in African Fusarium oxysporum f.sp. elaeidis Strains Pathogenesis During Screening Nigerian Susceptible and Tolerant Oil Palm (Elaeis guineensis Jacq.) Genotypes
Source: Front Cell Infect Microbiol. 2020 Oct 6;10:552394. doi: 10.3389/fcimb.2020.552394 (PMC7573130; doi:10.3389/fcimb.2020.552394)
Supplement: Supplementary file 9 [file Data_Sheet_9.docx]

**Supplementary Material: Table S4: Disease severity (%) on genotypes by *Fusarium* strains**

| ***Fusarium* strains** | **Genotypes** | **Sum of ratings** | **Total rating** | **Disease Severity (%)** |
| --- | --- | --- | --- | --- |
| **Strain (1)** | **1** | 21 | 27 | 15 |
|  | **2** | 6 | 27 | 4 |
|  | **3** | 9 | 27 | 6 |
|  | **4** | 15 | 27 | 11 |
|  | **5** | 5 | 27 | 3 |
|  | **6** | 6 | 27 | 4 |
|  | **7** | 3 | 27 | 2 |
|  | **8** | 7 | 42 | 0 |
| **Strain (4)** | **1** | 112 | 26 | 86 |
|  | **2** | 105 | 27 | 77.7 |
|  | **3** | 116 | 27 | 85.9 |
|  | **4** | 101 | 27 | 74.8 |
|  | **5** | 114 | 27 | 84 |
|  | **6** | 84 | 27 | 62 |
|  | **7** | 63 | 25 | 50 |
|  | **8** | 0 | 37 | 0 |

**Supporting information: Table S4: Disease severity (%) on genotypes by *Fusarium* strains (Contd)**

| ***Fusarium* strains** | **Genotypes** | **Sum of ratings** | **Total rating** | **Disease Severity (%)** |
| --- | --- | --- | --- | --- |
| **Strain (13)** | **1** | 16 | 26 | 12 |
|  | **2** | 18 | 27 | 13 |
|  | **3** | 14 | 27 | 10 |
|  | **4** | 7 | 27 | 5 |
|  | **5** | 5 | 27 | 3.7 |
|  | **6** | 3 | 27 | 2.2 |
|  | **7** | 3 | 27 | 2.2 |
|  | **8** | 42 | 9 | 4.2 |
| **Strain (CRT)** | **1** | 66 | 27 | 48.8 |
|  | **2** | 65 | 27 | 48.1 |
|  | **3** | 74 | 27 | 54.8 |
|  | **4** | 34 | 28 | 24.2 |
|  | **5** | 49 | 27 | 36.29 |
|  | **6** | 45 | 18 | 50 |
|  | **7** | 69 | 27 | 51.1 |
|  | **8** | 7 | 42 | 3.3 |
