# Supplementary material for: Molecular Identification of Secreted Effector Genes Involved in African Fusarium oxysporum f.sp. elaeidis Strains Pathogenesis During Screening Nigerian Susceptible and Tolerant Oil Palm (Elaeis guineensis Jacq.) Genotypes
Source: Front Cell Infect Microbiol. 2020 Oct 6;10:552394. doi: 10.3389/fcimb.2020.552394 (PMC7573130; doi:10.3389/fcimb.2020.552394)
Supplement: Supplementary file 10 [file Data_Sheet_10.docx]

**Supplementary Material: Table S5: Allelic scores count and frequencies obtained from *Fusarium oxysporum* f.sp. *elaeidis* using Inter-simple sequence repeat (ISSR) markers**

| **Marker** | **Allele** | **Count** | **Frequency** |
| --- | --- | --- | --- |
| **ISSR 858** | 0/0/0/0/0/0/0/0 | 4 | 0.2353 |
| **ISSR 858** | 0/0/0/0/1/0/0/0 | 1 | 0.0588 |
| **ISSR 858** | 0/0/0/1/1/0/0/0 | 2 | 0.1176 |
| **ISSR 858** | 0/0/1/1/0/0/0/0 | 1 | 0.0588 |
| **ISSR 858** | 0/0/1/1/1/0/0/0 | 5 | 0.2941 |
| **ISSR 858** | 0/1/1/0/0/0/0/0 | 1 | 0.0588 |
| **ISSR 858** | 0/1/1/1/1/0/0/0 | 1 | 0.0588 |
| **ISSR 858** | 0/1/1/1/1/1/1/0 | 1 | 0.0588 |
| **ISSR 858** | 1/0/0/1/1/0/0/0 | 1 | 0.0588 |
| **ISSR 818** | 0/0/0/0/0/0/0/0 | 6 | 0.3529 |
| **ISSR 818** | 0/0/0/1/0/0/0/0 | 1 | 0.0588 |
| **ISSR 818** | 0/0/1/0/0/0/0/0 | 1 | 0.0588 |
| **ISSR 818** | 0/0/1/0/1/0/0/0 | 1 | 0.0588 |
| **ISSR 818** | 0/0/1/1/0/0/0/0 | 3 | 0.1765 |
| **ISSR 818** | 0/1/1/1/0/0/0/0 | 1 | 0.0588 |
| **ISSR 818** | 0/1/1/1/1/1/0/0 | 1 | 0.0588 |
| **ISSR 818** | 1/0/1/1/0/0/0/0 | 1 | 0.0588 |
| **ISSR 818** | 1/0/1/1/0/1/0/0 | 1 | 0.0588 |
| **ISSR 818** | 1/0/1/1/1/1/0/0 | 1 | 0.0588 |
| **ISSR 836** | 0/0/0/0/0/0/0/0 | 3 | 0.1765 |
| **ISSR 836** | 0/0/0/0/0/0/1/0 | 1 | 0.0588 |
| **ISSR 836** | 0/0/0/0/1/0/1/0 | 2 | 0.1176 |
| **ISSR 836** | 0/0/1/0/1/0/0/0 | 1 | 0.0588 |
| **ISSR 836** | 0/0/1/0/1/0/1/0 | 2 | 0.1176 |
| **ISSR 836** | 0/0/1/0/1/1/1/0 | 1 | 0.0588 |
| **ISSR 836** | 0/0/1/1/0/0/1/0 | 1 | 0.0588 |
| **ISSR 836** | 0/1/1/0/1/0/0/0 | 1 | 0.0588 |
| **ISSR 836** | 0/1/1/0/1/0/1/0 | 1 | 0.0588 |
| **ISSR 836** | 1/1/0/0/1/0/0/0 | 1 | 0.0588 |
| **ISSR 836** | 1/1/1/1/1/0/1/0 | 1 | 0.0588 |
| **ISSR 836** | 1/1/1/1/1/1/1/0 | 2 | 0.1176 |
| **ISSR 890** | 0/0/0/0/0/0/0/0 | 2 | 0.1176 |
| **ISSR 890** | 0/0/0/0/0/0/0/1 | 3 | 0.1765 |

**Supporting information: Table S5 (Contd): Allelic scores count and frequencies obtained from *Fusarium oxysporum* f.sp. *elaeidis* using Inter-simple sequence repeat (ISSR) markers**

| **Marker** | **Allele** | **Count** | **Frequency** |
| --- | --- | --- | --- |
| **ISSR 890** | 0/0/0/1/1/0/0/0 | 1 | 0.0588 |
| **ISSR 890** | 0/0/0/1/1/0/1/0 | 1 | 0.0588 |
| **ISSR 890** | 0/0/1/0/0/0/0/1 | 2 | 0.1176 |
| **ISSR 890** | 0/0/1/1/0/0/0/1 | 1 | 0.0588 |
| **ISSR 890** | 0/0/1/1/1/1/1/1 | 1 | 0.0588 |
| **ISSR 890** | 0/1/0/1/0/0/0/1 | 1 | 0.0588 |
| **ISSR 890** | 0/1/1/0/0/1/1/0 | 1 | 0.0588 |
| **ISSR 890** | 1/0/1/0/1/0/0/1 | 1 | 0.0588 |
| **ISSR 890** | 1/0/1/0/1/0/1/1 | 1 | 0.0588 |
| **ISSR 890** | 1/1/0/1/1/1/1/1 | 1 | 0.0588 |
| **ISSR 890** | 1/1/1/1/1/1/1/1 | 1 | 0.0588 |
| **ISSR 827** | 0/0/0/0/0/0/0/0 | 6 | 0.3529 |
| **ISSR 827** | 0/0/0/0/0/1/0/0 | 1 | 0.0588 |
| **ISSR 827** | 0/0/0/0/0/1/0/1 | 1 | 0.0588 |
| **ISSR 827** | 0/0/0/0/0/1/1/0 | 2 | 0.1176 |
| **ISSR 827** | 0/0/0/0/1/1/0/0 | 1 | 0.0588 |
| **ISSR 827** | 0/0/1/0/0/1/0/0 | 2 | 0.1176 |
| **ISSR 827** | 0/0/1/0/0/1/1/0 | 1 | 0.0588 |
| **ISSR 827** | 0/0/1/1/0/1/1/0 | 1 | 0.0588 |
| **ISSR 827** | 0/1/1/1/0/0/0/0 | 1 | 0.0588 |
| **ISSR 827** | 1/0/1/0/0/1/0/0 | 1 | 0.0588 |
| **ISSR 811** | 0/0/0/0/0/0/0/0 | 1 | 0.0588 |
| **ISSR 811** | 0/0/0/0/0/1/0/0 | 1 | 0.0588 |
| **ISSR 811** | 0/0/0/1/1/0/0/0 | 1 | 0.0588 |
| **ISSR 811** | 0/0/0/1/1/1/0/0 | 1 | 0.0588 |
| **ISSR 811** | 1/0/0/0/0/1/0/0 | 1 | 0.0588 |
| **ISSR 811** | 1/0/0/0/1/1/0/0 | 1 | 0.0588 |
| **ISSR 811** | 1/0/0/1/1/0/0/0 | 1 | 0.0588 |
| **ISSR 811** | 1/0/0/1/1/1/0/0 | 2 | 0.1176 |
| **ISSR 811** | 1/1/0/0/1/1/0/0 | 1 | 0.0588 |
| **ISSR 811** | 1/1/0/1/1/0/0/0 | 2 | 0.1176 |
| **ISSR 811** | 1/1/0/1/1/1/0/0 | 2 | 0.1176 |
| **ISSR 811** | 1/1/1/1/1/0/0/0 | 2 | 0.1176 |
| **ISSR 811** | 1/1/1/1/1/1/0/0 | 1 | 0.0588 |
| **ISSR 901** | 0/0/0/0/0/0/0/0 | 1 | 0.0588 |
| **ISSR 901** | 0/0/0/0/0/1/0/0 | 1 | 0.0588 |
| **ISSR 901** | 0/0/0/1/1/0/0/0 | 1 | 0.0588 |
| **ISSR 901** | 0/0/0/1/1/1/0/0 | 1 | 0.0588 |
| **ISSR 901** | 1/0/0/0/0/1/0/0 | 1 | 0.0588 |
| **ISSR 901** | 1/0/0/0/1/1/0/0 | 1 | 0.0588 |
| **ISSR 901** | 1/0/0/1/1/0/0/0 | 1 | 0.0588 |

**Supporting information: Table S5 (Contd): Allelic scores count and frequencies obtained from *Fusarium oxysporum* f.sp. *elaeidis* using Inter-simple sequence repeat (ISSR) markers**

| **Marker** | **Allele** | **Count** | **Frequency** |
| --- | --- | --- | --- |
| **ISSR 901** | 1/0/0/1/1/1/0/0 | 2 | 0.1176 |
| **ISSR 901** | 1/1/0/0/1/1/0/0 | 1 | 0.0588 |
| **ISSR 901** | 1/1/0/1/1/0/0/0 | 2 | 0.1176 |
| **ISSR 901** | 1/1/0/1/1/1/0/0 | 2 | 0.1176 |
| **ISSR 901** | 1/1/1/1/1/0/0/0 | 2 | 0.1176 |
| **ISSR 901** | 1/1/1/1/1/1/0/0 | 1 | 0.0588 |
| **ISSR 842** | 0/0/0/0/0/0/0/0 | 4 | 0.2353 |
| **ISSR 842** | 0/0/0/0/1/0/0/0 | 1 | 0.0588 |
| **ISSR 842** | 0/0/0/1/1/0/0/0 | 2 | 0.1176 |
| **ISSR 842** | 0/0/1/1/0/0/0/0 | 1 | 0.0588 |
| **ISSR 842** | 0/0/1/1/1/0/0/0 | 5 | 0.2941 |
| **ISSR 842** | 0/1/1/0/0/0/0/0 | 1 | 0.0588 |
| **ISSR 842** | 0/1/1/1/1/0/0/0 | 1 | 0.0588 |
| **ISSR 842** | 0/1/1/1/1/1/1/0 | 1 | 0.0588 |
| **ISSR 842** | 1/0/0/1/1/0/0/0 | 1 | 0.0588 |
| **HB-10** | 0/0/0/0/0/0/0/0 | 2 | 0.1176 |
| **HB-10** | 0/0/0/1/0/0/0/0 | 2 | 0.1176 |
| **HB-10** | 1/0/0/0/0/0/0/0 | 2 | 0.1176 |
| **HB-10** | 1/0/0/1/0/0/0/0 | 3 | 0.1765 |
| **HB-10** | 1/1/0/0/0/0/0/0 | 1 | 0.0588 |
| **HB-10** | 1/1/0/1/0/0/0/0 | 4 | 0.2353 |
| **HB-10** | 1/1/1/1/0/0/0/0 | 3 | 0.1765 |

**Allele frequency, number of alleles, genetic diversity and polymorphic information content of ISSR markers**

| **Marker** | **Major allele frequency** | **No. of obs.** | **Allele No** | **Gene Diversity** | **PIC** |
| --- | --- | --- | --- | --- | --- |
| **ISSR 858** | 0.2941 | 17.0000 | 9.0000 | 0.8235 | 0.8032 |
| **ISSR 818** | 0.3529 | 17.0000 | 10.0000 | 0.8166 | 0.7996 |
| **ISSR 836** | 0.1765 | 17.0000 | 12.0000 | 0.8997 | 0.8912 |
| **ISSR 890** | 0.1765 | 17.0000 | 13.0000 | 0.9066 | 0.8993 |
| **ISSR 827** | 0.3529 | 17.0000 | 10.0000 | 0.8235 | 0.8084 |
| **ISSR 811** | 0.1176 | 17.0000 | 13.0000 | 0.9135 | 0.9069 |
| **ISSR 901** | 0.1176 | 17.0000 | 13.0000 | 0.9135 | 0.9069 |
| **ISSR 842** | 0.2941 | 17.0000 | 9.0000 | 0.8235 | 0.8032 |
| **HB-10** | 0.2353 | 17.0000 | 7.0000 | 0.8374 | 0.8165 |
| **Mean** | 0.2176 | 17.0000 | 11.3000 | 0.8699 | 0.8573 |

PIC= Polymorphic information content

Coordinate positions of *Fusarium* strains alleles

| ***Fusarium* strains** | **Axis 1** | | **Axis 2** | | **Axis 3** | | **Axis 4** | | **Axis 5** | |
| --- | --- | --- | --- | --- | --- | --- | --- | --- | --- | --- |
|  | Coord. | Cos² | Coord. | Cos² | Coord. | Cos² | Coord. | Cos² | Coord. | Cos² |
| 1 | 0.8877 | 986 | 0.0986 | 12 | -0.0192 | 0 | -0.0254 | 1 | 0.0048 | 0 |
| 2 | -0.0815 | 69 | 0.2386 | 594 | 0.1083 | 122 | -0.0005 | 0 | -0.0316 | 10 |
| 3 | -0.0843 | 77 | 0.2387 | 616 | 0.0978 | 103 | 0.0556 | 33 | -0.0788 | 67 |
| 4 | -0.0826 | 129 | -0.0611 | 71 | -0.0091 | 2 | -0.0644 | 79 | 0.0613 | 71 |
| 5 | -0.0681 | 44 | 0.0627 | 38 | -0.1886 | 340 | -0.1554 | 231 | -0.0202 | 4 |
| 6 | -0.0630 | 49 | -0.0934 | 109 | -0.0322 | 13 | -0.1414 | 248 | 0.1337 | 222 |
| 7 | -0.0645 | 36 | 0.1297 | 145 | -0.2398 | 496 | 0.0920 | 73 | 0.1083 | 101 |
| 8 | -0.0656 | 58 | -0.0930 | 117 | -0.1621 | 356 | 0.0099 | 1 | 0.1441 | 282 |
| 9 | -0.0694 | 61 | -0.0385 | 19 | -0.1064 | 143 | -0.1165 | 171 | -0.0941 | 112 |
| 10 | -0.0602 | 40 | -0.0692 | 53 | -0.1286 | 183 | -0.1261 | 176 | -0.1589 | 280 |
| 11 | -0.0763 | 52 | 0.2768 | 680 | 0.0384 | 13 | 0.0275 | 7 | 0.0360 | 11 |
| 12 | 0.0120 | 1 | -0.3321 | 713 | -0.0132 | 1 | 0.0340 | 7 | -0.0304 | 6 |
| 13 | 0.0516 | 13 | -0.3487 | 579 | 0.0474 | 11 | 0.1418 | 96 | -0.1111 | 59 |
| 14 | -0.0504 | 20 | 0.0893 | 64 | -0.1689 | 228 | 0.2587 | 535 | -0.0028 | 0 |
| 15 | -0.0916 | 142 | 0.1038 | 183 | 0.0487 | 40 | 0.0171 | 5 | -0.1349 | 309 |
| 16 | -0.0221 | 4 | -0.0951 | 69 | 0.2847 | 615 | -0.0132 | 1 | 0.0668 | 34 |
| 17 | -0.0604 | 34 | 0.1098 | 112 | 0.3020 | 848 | -0.0425 | 17 | 0.0408 | 15 |
